# OsIDD7 integrates signaling networks for arbuscular mycorrhizal symbiosis

**DOI:** 10.1038/s41467-026-77162-3

**Published:** 2026-08-31

**Authors:** Pei-Jung Chen, Yi-Tsung Tu, Yu-Ting Chang, Man-Chi Ho, Wan-Ning Kuo, Zheng-Lin Guo, Kai-Chieh Chang, Yi-Zheng Lin, Hsuan-Chih Shih, Yi-Sheng Cheng, Shu-Yi Yang

**Affiliations:** 1 Institute of Plant Biology, National Taiwan University, Taipei, Taiwan; 2 Department of Life Science, National Taiwan University, Taipei, Taiwan

**Keywords:** Arbuscular mycorrhiza, Plant molecular biology, Plant signalling

## Abstract

Arbuscular mycorrhizal (AM) symbiosis is a widespread mutualism between plant roots and Glomeromycotina fungi that enables nutrient exchange through arbuscules. Although many transcriptional regulators of AM symbiosis have been identified, the role of the INDETERMINATE DOMAIN (IDD) proteins remain unknown. We show that rice *IDD7* expression is strongly induced in arbuscule-containing cells, and its loss markedly reduces fungal colonization and disrupts arbuscule development. IDD7 is required for induction of genes associated with fatty acid biosynthesis, nutrient transport, and symbiotic signaling during AM symbiosis. IDD7 interacts with the transcription factors SLENDER RICE 1 (SLR1) and PHOSPHATE STARVATION RESPONSE 2 (PHR2) through its conserved TQDFLG domain. Electrophoretic mobility shift assays show that IDD7 binds multiple promoter motifs. Together with SLR1 and PHR2, IDD7 synergistically activates the promoters of *PHOSPHATE TRANSPORTER 11* (*PT11*) and the AM-associated transcription factors *PHR2*, *REQUIRED FOR ARBUSCULAR MYCORRHIZATION 1 (RAM1), WRINKLED 5a (WRI5a)*, and *CYCLOPS*. Moreover, IDD7 is required for *PHR2* expression in arbuscule-containing cells. Here, we show that IDD7 is a central transcriptional regulator in arbuscule-containing cells.

## Abstract (web summary)

Here the authors identify IDD7 as a regulator of arbuscular mycorrhizal symbiosis in rice. They reveal how IDD7 coordinates transcriptional networks that promote beneficial interactions between rice roots and mycorrhizal fungi.

## Introduction

Arbuscular mycorrhizal (AM) symbiosis represents an endosymbiotic relationship between plant roots and fungi belonging to the phylum Glomeromycotina, which dates back 400–450 million years^1^. Host plants supply photosynthesis-derived carbon to AM fungi, while AM fungi provide essential mineral nutrients, such as inorganic phosphate (Pi) and ammonium, to plants^2^. Before contact, plants secrete strigolactones and flavonoids, while AM fungi release mycorrhizal factors (Myc factors) to activate symbiosis signaling. Fungal hyphae form hyphopodia, induce a prepenetration apparatus, develop arbuscules for nutrient exchange, generate lipid-rich vesicles for nutrient storage, and ultimately produce spores^1^.

Previous studies have identified several critical components and transcription factors (TFs) involved in the signaling and molecular regulation of AM accommodation. For example, the recognition of Myc factors activates the common symbiosis signaling pathway (CSSP), which is essential for both AM and root nodule symbiosis^3^. CCaMK, a calcium/calmodulin-dependent kinase, decodes calcium spikes and activates the transcription factor CYCLOPS/IPD3, initiating downstream gene expression^4^. The AP2/ERF domain transcription factor WRI5a in *M. truncatula* and its homolog, CTTC MOTIF-BINDING TRANSCRIPTION FACTOR1 (CBX1) in *L. japonicus,* serve as critical regulators of nutrient exchange, particularly in lipid biosynthesis and transport as well as phosphate transport between AM fungi and their host plants^5,6^. Recent work further demonstrated that LjCBX1 forms a transcriptional complex with RAM1 to coordinately regulate nutrient exchange genes, highlighting the importance of combinatorial transcription factor modules during arbuscule development^7^. PHOSPHATE STARVATION RESPONSE (PHR) transcription factors, which belong to the MYB transcription factor family, play essential roles in managing both the phosphate starvation response (PSR) and AM symbiosis^8–10^. REDUCED ARBUSCULAR MYCORRHIZA1 (RAM1), a GRAS protein, is central to arbuscule development^9,11–14^. In addition to RAM1, several other GRAS proteins, such as REQUIRED FOR ARBUSCULE DEVELOPMENT1 (RAD1), NODULATION SIGNALING PATHWAY2 (NSP2), and DELLA-INTERACTING PROTEIN1 (DIP1), have been implicated in AM symbiosis^4^. Another GRAS protein, DELLA, is essential for arbuscule formation and interacts with CCaMK and CYCLOPS to promote *RAM1* activation^13,15^. However, most GRAS proteins lack DNA-binding ability, suggesting a reliance on partners such as C2H2 zinc finger (ZF) transcription factors from the INDETERMINATE DOMAIN (IDD) family^16^. IDD proteins, which are known for their roles in growth and stress responses^17^, are characterized by a highly conserved ID domain, comprising two canonical C_2_H_2_ and two C_2_HC zinc finger motifs, which facilitate their ability to bind DNA^18^. However, the potential roles of IDD proteins in plant‒microbe interactions remain unexplored.

Here, we show that rice IDD7 is a central transcriptional regulator of AM symbiosis. *IDD7* is strongly induced in arbuscule-containing cells and is required for fungal colonization, arbuscule development, and proper induction of genes associated with fatty acid biosynthesis, nutrient transport, and symbiotic signaling. IDD7 interacts with SLR1 and PHR2 and binds multiple promoter motifs to regulate symbiosis-associated genes. Together with SLR1 and PHR2, IDD7 synergistically activates the promoters of *PT11*, *PHR2*, *RAM1*, *WRI5a*, and *CYCLOPS*. Moreover, IDD7 is required for *PHR2* expression in arbuscule-containing cells. These findings establish IDD7 as a key component of the transcriptional network coordinating arbuscular mycorrhizal symbiosis.

## Results

### OsIDD7 expression in arbuscule-containing cells

**Fig. 1:**
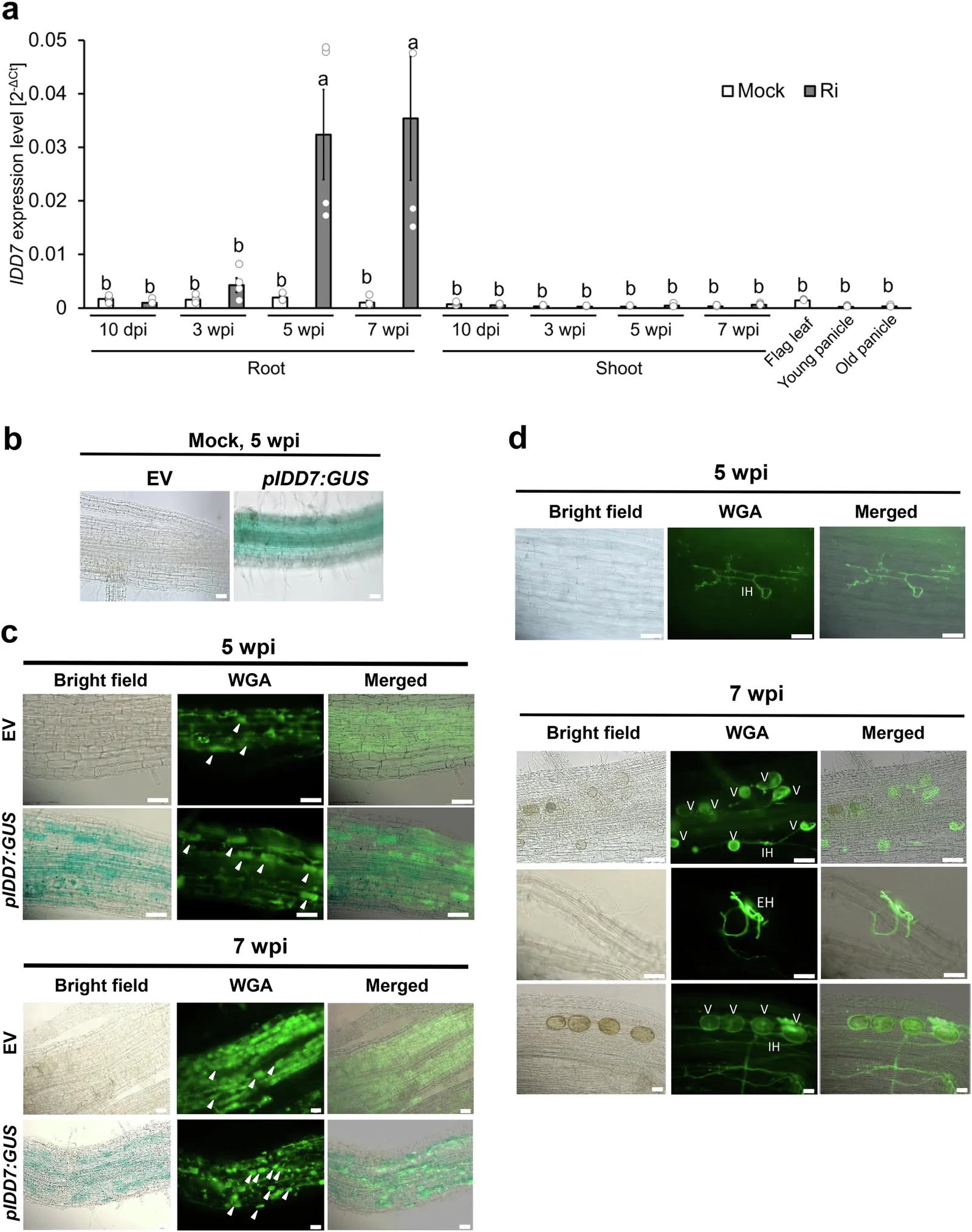
Expression pattern of *IDD7* during AM symbiosis. **a** Gene expression pattern of *IDD7*. Seven-day-old rice seedlings (*O. sativa* ssp. *japonica* cv. Nipponbare) were inoculated without (mock) or with AM fungi (*R. irregularis*, Ri) and grown under 25 μM Pi conditions. The plants were subsequently harvested at 10 days postinoculation (dpi) and 3, 5 and 7 weeks postinoculation (wpi). Young panicles, panicles that were 4–5 cm long; old panicles, panicles that reached the heading stage and emerged completely. Gene expression was analyzed in the roots and normalized to that of *Cyclophilin2* (*CYC2*) (2^−ΔCt^). Data are presented as mean ± SEM. Each biological replicate consisted of root and shoot samples collected from the same plants, with samples pooled from 5, 4, 3, and 2 independently grown plants at 10 dpi, 3, 5, and 7 wpi, respectively. For flag leaves, young panicles, and old panicles, each biological replicate was obtained from one independently grown plant. Sample sizes were *n* = 4 biologically independent pooled samples for roots and shoots at each time point and *n* = 3 biologically independent samples for flag leaves, young panicles, and old panicles. Different letters represent significant differences between the treatments (*P* < 0.05, one-way ANOVA followed by a least significant difference post hoc test). Exact *P* values for the indicated comparisons are provided in the Source Data. **b** Spatial expression pattern of *pIDD7:GUS* at 5 wpi under mock conditions. **c** GUS signals driven by the upstream 2043 bp *IDD7* promoter in roots containing arbuscules. **d** *IDD7* promoter activity in roots containing intraradical hyphae, vesicles, or extraradical hyphae. Empty vector (EV) control (T2 generation) and *pIDD7:GUS* transgenic rice plants (T2 generation) were inoculated without AM fungi (Mock) or with AM fungi (*R. irregularis*, Ri), grown under 25 μM Pi conditions, and harvested at specific time points. Bars = 50 μm. Image overlays of bright field (BF) and wheat germ agglutinin (WGA) signals are shown. White arrowheads, arbuscules; V vesicles, IH intraradical hyphae, EH extraradical hyphae. The results for at least two independent lines were similar.

Among the 15 OsIDDs identified by Zhang et al. ^19^, *IDD7* is a candidate TF with potential involvement in arbuscular mycorrhizal (AM) symbiosis on the basis of its consistent AM-inducible expression in mycorrhizal roots (Supplementary Table 1)^9,20,21^. *IDD7* transcripts were highly expressed in mycorrhizal roots at 5 and 7 weeks postinoculation (wpi) but were expressed at low levels in shoots, flag leaves, and panicles (Fig. 1a). To assess whether additional IDD family members respond to AM symbiosis, we performed qPCR analysis on selected genes. *IDD9* and *IDD14* showed significant induction at later stages of colonization (5–7 and 3–7 wpi, respectively). *IDD12*, although not identified as a DEG in the RNA-seq datasets, displayed induction at 5 wpi, suggesting possible stage-specific or condition-dependent regulation. In contrast, *IDD4.3* and *IDD13.1* did not exhibit significant changes in expression (Supplementary Fig. 1a). *IDD11*, which showed differential expression in one RNA-seq dataset, was not included in the qPCR analysis.

Promoter-GUS assays revealed baseline *IDD7* expression in vascular and cortical tissues under mock conditions (Fig. 1b), whereas AM colonization redirected GUS signals specifically to arbuscule-containing cells at 5 and 7 wpi (Fig. 1c). No signal was detected near other fungal structures (Fig. 1d), suggesting that *IDD7* expression was specifically associated with arbuscule-containing cells. Subcellular localization in *N. benthamiana* leaves confirmed that IDD7-EGFP fusions localized to the nucleus, overlapping with NLS-mCherry signals (Supplementary Fig. 1b).

### OsIDD7 is crucial in AM symbiosis

To assess the role of IDD7 in AM symbiosis, the *idd7* T-DNA knockout mutant and two complementation lines (*idd7*^*IDD7*^), expressing *IDD7* under its native promoter (2043 bp) with a T35S terminator, were analyzed. The *idd7* mutant harbored a T-DNA insertion in the third exon (Supplementary Fig. 2a–d), with minimal growth differences observed among the transgenic lines (Supplementary Fig. 2e and f).

**Fig. 2:**
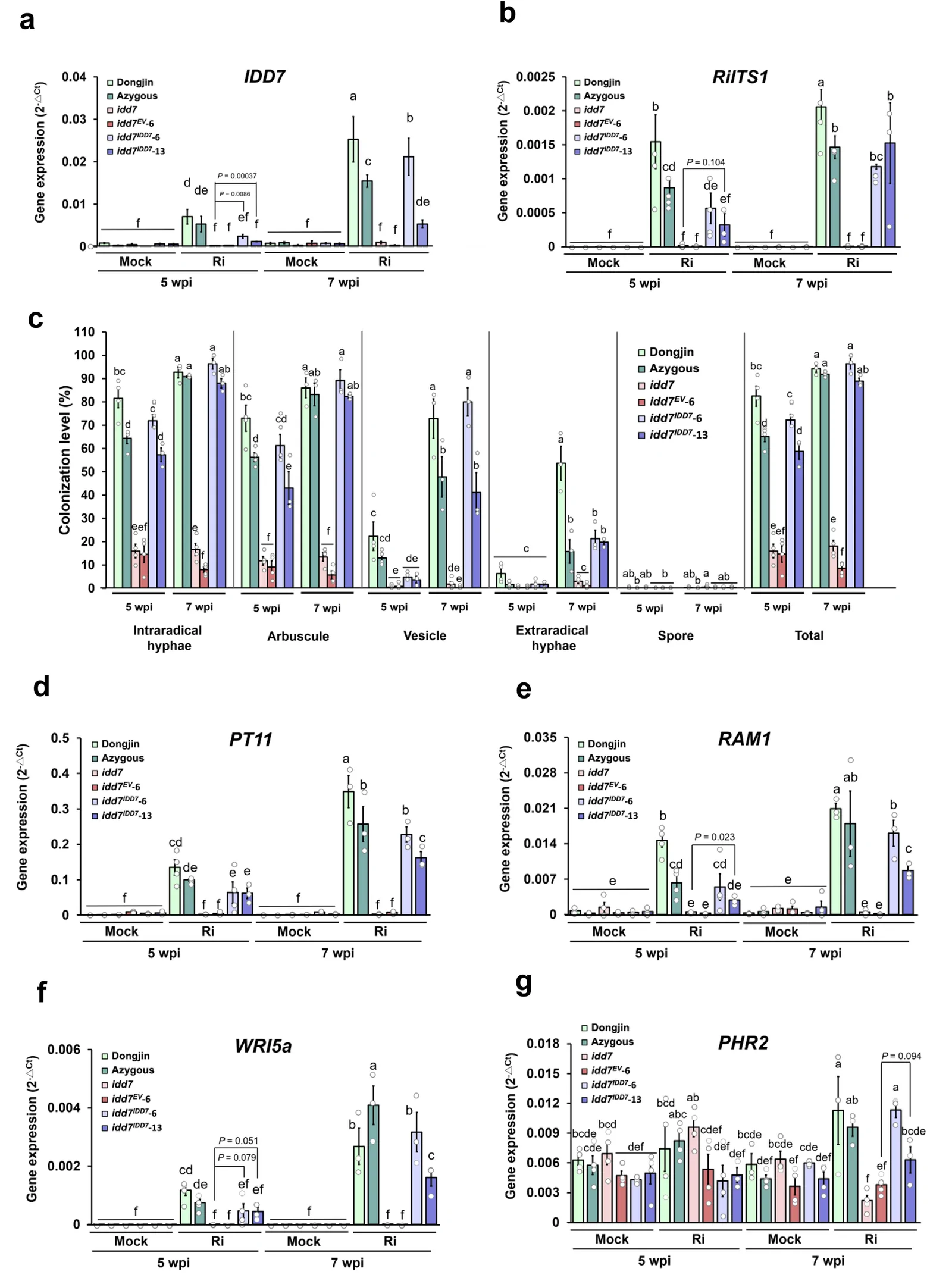
IDD7 is required for AM symbiosis. **a** *IDD7* expression; **b** *RiITS1* expression; **c** fungal colonization level; **d** *PT11* expression; **e** *RAM1* expression; **f** *WRI5a* expression; **g** *PHR2* expression in the roots of different transgenic rice plants. Control plants (Dongjin and Azygous, the segregating wild type of the *idd7* mutant), the *idd7* T-DNA knockout mutant (*idd7*), the *idd7* mutant transformed with EV (*idd7*^*EV*^) (T1 generation) and two complementation lines referred to as the *idd7* mutant transformed with the *pIDD7:IDD7* CDS construct (*idd7*^*IDD7*^) (T4 generation) were inoculated without (Mock) or with the AM fungus *R. irregularis* (Ri), grown under 25 μM Pi conditions and harvested at 5 and 7 weeks postinoculation (wpi). Gene expression in the roots was analyzed by qRT‒PCR and normalized to that of *Cyclophilin2* (*CYC2*) (2^–ΔCt^). Data are presented as mean ± SEM. Each biological replicate consisted of roots pooled from two independently grown plants. For panels **a**–**g**, *n* = 3 biologically independent replicates for 5wpi Mock *idd7*^*IDD7*^*-6*, 5wpi Ri *idd7*^*IDD7*^*-13*, 7 wpi Mock Dongjin, and 7 wpi Ri Dongjin, Azygous, *idd7*^*IDD7*^*-6*, and *idd7*^*IDD7*^*-13*; *n* = 4 biologically independent replicates for all other groups. Different letters represent significant differences (*P* < 0.05, one-way ANOVA followed by a least significant difference post hoc test); exact *P* values for the post hoc pairwise comparisons are provided in the Source Data. For the indicated comparisons with the *idd7* mutant, exact *P* values are shown in the figure and were determined using one-sided Welch’s *t*-tests.

Gene expression analysis confirmed the AM-induced *IDD7* expression in wild-type roots (Fig. 2a). At 5 wpi, *IDD7* expression was significantly higher in *idd7*^*IDD7*^ lines than in *idd7*, and the fungal marker *RiITS1* (*Rhizophagus irregularis internal transcribed spacer 1*) showed increased abundance, consistent with enhanced fungal colonization. By 7 wpi, the *RiITS1* levels of both *idd7*^*IDD7*^ lines were comparable to those of the wild type, although only one line matched the wild-type *IDD7* expression (Fig. 2a, b). Differences between independent complementation lines likely reflect positional effects of transgene insertion. AM colonization—measured by intraradical hyphae, arbuscules, and total colonization—was markedly reduced in *idd7* and *idd7*^*EV*^ at both time points, including lower extraradical hyphae and vesicles at 7 wpi (Fig. 2c). The azygous segregant, derived from the *idd7* heterozygous background, was used as the primary wild-type control. At 5 wpi, overall colonization in the azygous line was lower than in the parental cultivar Dongjin, whereas at 7 wpi the levels were comparable. These defects were restored in *idd7*^*IDD7*^ lines to levels comparable to the azygous control. Arbuscule abundance increased from ~52% to ~85% in *idd7*^*IDD7*^ lines from 5 to 7 wpi but remained at ~10% in *idd7* and *idd7*^*EV*^. Normal symbiotic progression occurred only in the control and complementation lines.

**Fig. 3:**
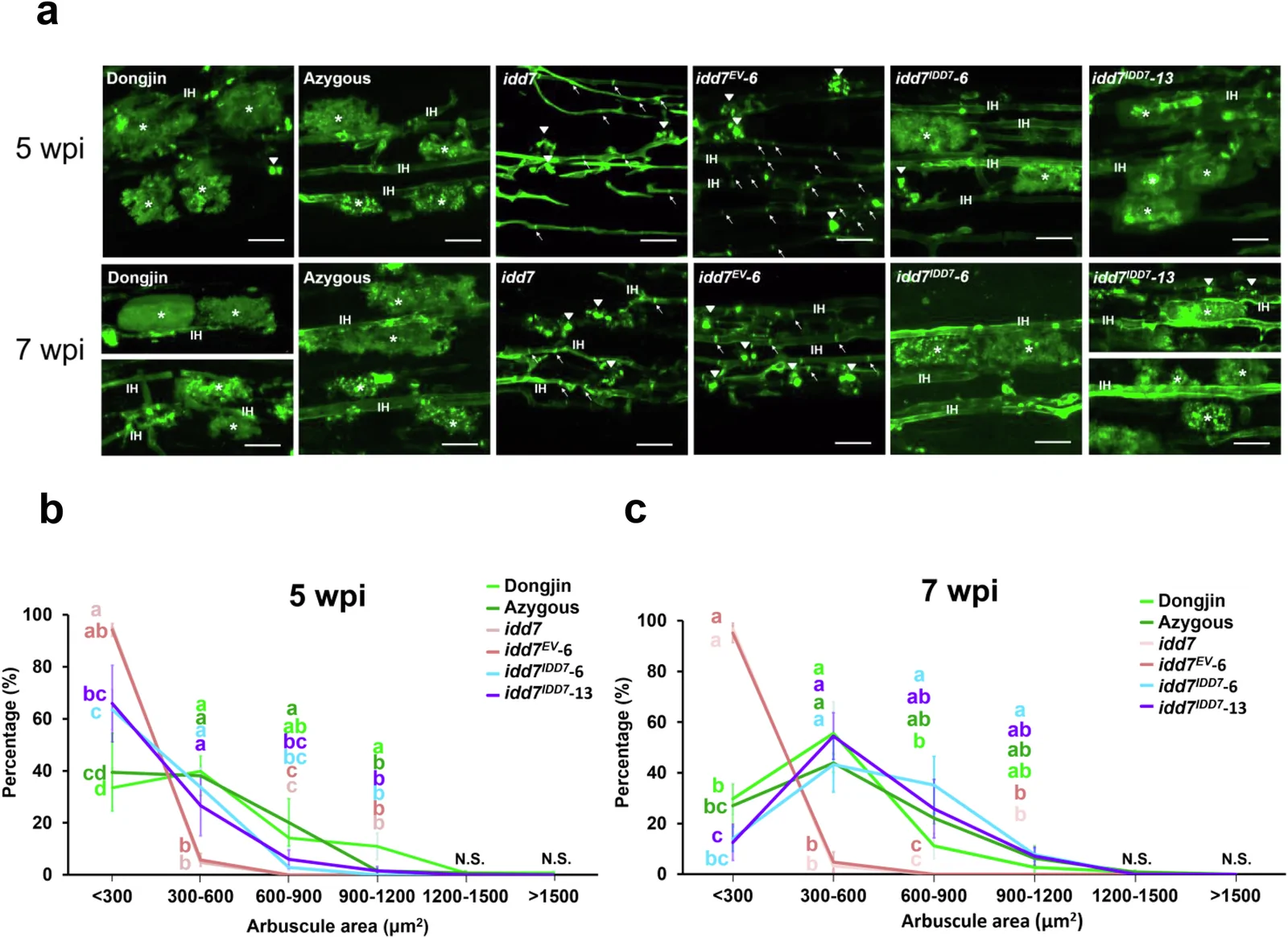
IDD7 is essential for arbuscule development. **a** The fungal structure. Asterisk: arbuscule; arrowhead: degenerative arbuscule; IH intraradical hyphae; arrow: septa. Bars = 20 μm. **b**, **c** The arbuscule area distribution at 5 and 7 wpi. For each biological replicate, the areas of 29–53 arbuscules were measured. Control plants (Dongjin and Azygous, the segregating wild type of the *idd7* mutant), the *idd7* T-DNA knockout mutant (*idd7*), the *idd7* mutant transformed with EV (*idd7*^*EV*^) (T1 generation) and two complementation lines referred to as the *idd7* mutant transformed with the *pIDD7:IDD7* CDS construct (*idd7*^*IDD7*^) (T4 generation) were inoculated without (Mock) or with the AM fungus *R. irregularis* (Ri), grown under 25 μM Pi conditions and harvested at 5 and 7 weeks postinoculation (wpi). Data are presented as mean ± SEM. One biological replicate consisted of two independently grown plants. For panel **b** (5 wpi), *n* = 3 biological replicates for *idd7*^*IDD7*^*-13* and 3 biological replicates for all other groups; for panel **c** (7 wpi), *n* = 4 biological replicates for *idd7* and *idd7*^*EV*^ and 3 biological replicates for all other groups. The 29–53 arbuscules measured per biological replicate represent subsamples from these biological replicates and were not treated as independent biological replicates. Different letters represent significant differences (*P* < 0.05, one-way ANOVA followed by a least significant difference post hoc test). Exact *P* values for the indicated comparisons are provided in the Source Data.

Further analysis revealed abnormal arbuscule development in *idd7* and *idd7*^*EV*^, including degeneration and hyphal septation, indicating impaired symbiosis (Fig. 3a). Arbuscule size was reduced in the mutants (mostly <300 μm²), whereas the size distribution in the *idd7*^*IDD7*^ lines was restored to 300–600 μm² at 5 wpi and reached 300–900 μm² at 7 wpi, which was comparable to that of the wild type (Fig. 3b and c). These results indicate that IDD7 was essential for proper arbuscule development and AM fungal maintenance.

### IDD7 is associated with the regulation of nutrient and signaling genes during AM symbiosis

**Fig. 4:**
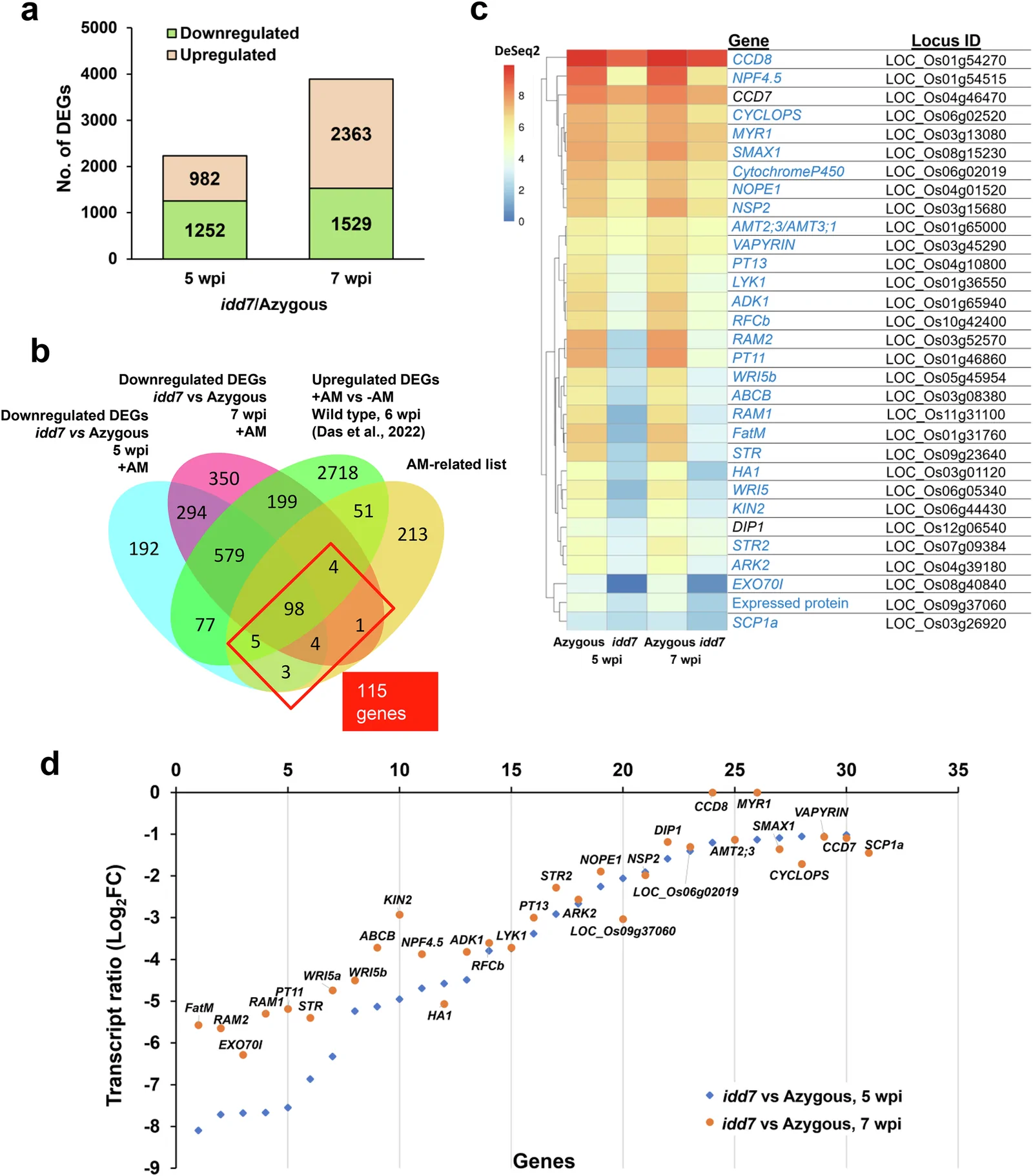
Comparative analysis of the RNA-seq data reveals genes whose expression is associated with IDD7 during AM symbiosis, including a list of AM-relevant genes. **a** Number of up- and downregulated differentially expressed genes (DEGs) in *idd7* vs. Azygous plants, with a |fold change| ≥ 2 and adjusted *p* < 0.05. **b** Venn diagram showing the overlap of downregulated DEGs in ‘*idd7* vs. Azygous’ and upregulated DEGs in wild type ‘+AM vs. –AM’ (Das et al.^20^) and ‘AM-related list’^20,22^. The wild type used in Das et al. ^20^ is Nipponbare. **c** Heatmap representing the transcript level of the DeSeq2 value for a subset of genes that were downregulated in the *idd7* mutant, highlighted in (**b**), and further shortlisted to genes previously reported to be functionally important during AM symbiosis in rice or other plant species^22^. Columns are hierarchically clustered using the average linkage of DeSeq2 values. The genes highlighted in blue are AM-induced genes according to previous RNA-seq analyses^20^. **d** Transcript ratios of *idd7* versus Azygous at 5 and 7 weeks postinoculation (wpi) for the 31 downregulated DEGs listed in (**c**), ranked on the basis of their *idd7* versus Azygous ratios at 5 wpi. FC fold change. Azygous and *idd7* mutants were inoculated with the AM fungus *R. irregularis* (Ri), grown under 25 μM Pi conditions, and harvested at 5 and 7 weeks postinoculation (wpi). RNA-seq was performed using the roots of 3 biological replicates, and each replicate comprised 3 plants.

To investigate the role of IDD7 in regulating downstream genes during AM symbiosis, RNA-seq was performed on the roots of *idd7* mutants and Azygous controls at 5 and 7 weeks postinoculation (wpi). *IDD7* and *RiITS1* expression was significantly reduced in the mutants (Supplementary Fig. 3a and b), which was consistent with the results from the complementation experiment (Fig. 2a, b). PCA revealed distinct transcriptome clustering by genotype and time (Supplementary Fig. 3c). DEGs were identified using a |fold-change| ≥ 2 and adjusted *p* < 0.05, with more DEGs detected at 7 wpi (Fig. 4a). The number of genes with reduced expression exceeded those with increased expression with a |log_2_-fold change| > 5 at both time points (Supplementary Fig. 3d, Supplementary Data 1), suggesting a broad impact for IDD7.

To link IDD7 to AM-related genes, a combined AM gene list from prior studies^20,22^ was used to identify 115 AM-related DEGs that were associated with IDD7-dependent expression changes (Fig. 4b). Among these genes, 107 were AM-induced genes^20^. Because *idd7* mutants exhibit reduced AM colonization, reduced expression of these AM-induced genes may partly reflect reduced fungal colonization rather than direct transcriptional regulation by IDD7. Heatmaps and dot plots of these genes, including known AM regulators in rice (Group I) and orthologs from other species (Group II; Guo et al. ^22^), revealed lower expression at 5 wpi than at 7 wpi, indicating delayed AM progression in *idd7* mutants (Fig. 4c, d). The affected genes were associated with strigolactone biosynthesis (e.g., *CCD7* and *CCD8*), common symbiosis signaling pathways (e.g., *CYCLOPS* and *NSP2*), fatty acid biosynthesis (e.g., *FatM* and *RAM2*), transcriptional regulation (*RAM1* and *WRI5a/b*), nutrient transport (e.g., *PT11/13*, *HA1*, *STR1/2*, *AMT2;3* and *NPF4.5*), symbiosis signaling (e.g., *ARK2*, *LYK1*, *ADK1* and *KIN2*), the N-acetylglucosamine (GlcNAc) transporter *NOPE1* and the repressor of AM symbiosis *SMAX1* (Fig. 4d). GO enrichment of the genes with reduced expression revealed significant terms related to lipid biosynthesis, receptor signaling, and isoprenoid/terpenoid metabolism at both 5 and 7 wpi (Supplementary Fig. 3e). KEGG analysis also revealed the fatty acid and diterpenoid pathways (Supplementary Fig. 3f). qRT‒PCR confirmed that *PT11*, *RAM1*, *WRI5a*, *FatM*, *NPF4.5*, and *KIN2* were significantly reduced in expression in *idd7* mutant and *idd7*^*EV*^ plants but restored in *idd7*^*IDD7*^ complementation lines (Fig. 2d–f and Supplementary Fig. 4a–c). PHR2—known to activate *PT11*, *RAM1*, and *WRI5a*^9^—was also reduced in expression in *idd7* mutants but upregulated in one of the complementation lines at 7 wpi (Fig. 2g). Although *CCaMK* was not a DEG, the expression of both CCaMK and *CYCLOPS* tended to decrease in the mutants but was restored in one of the *IDD7* complementation lines (Supplementary Fig. 4d and e). Together, these results indicate that IDD7 is required for the full induction of genes associated with lipid biosynthesis, nutrient transport, and symbiotic signaling. Whether individual genes are directly regulated by IDD7 requires additional promoter-binding evidence.

### IDD7-VP16 drives key promoter activity

To test whether IDD7 could contribute to the transcriptional regulation of key AM regulatory genes, we selected the promoters of *PHR2*, *PT11*, *RAM1*, and *WRI5a*—genes with well-established roles in AM symbiosis and reduced expression in *idd7*—and performed transactivation assays in *N. benthamiana*. Since IDD proteins often require partners^23^ and PHR2 directly activates these genes via P1BS motifs^9^, IDD7 fused with the VP16 activation domain (IDD7-VP16) was used, with PHR2 as a positive control. IDD7 alone failed to activate any promoter, whereas IDD7-VP16 significantly increased promoter activities (Supplementary Fig. 5b). To evaluate whether IDD7–VP16 broadly activates AM-associated promoters, we also examined the promoters of *ABCB* and *VAPYRIN*, two AM-related genes identified as down-regulated DEGs in the *idd7* mutant at both 5 and 7 wpi in our RNA-seq analysis (Supplementary Data 1). In contrast to the promoters of *PHR2*, *PT11*, *RAM1*, and *WRI5a*, *ABCB* and *VAPYRIN* did not exhibit robust promoter activation by IDD7–VP16 in transient transactivation assays (Supplementary Fig. 5b). Together, these results indicate that IDD7–VP16 activates a subset of AM-associated promoters in this transient-expression system.

### The conserved TQDFLG motif contributes to IDD7 interaction with SLR1 and PHR2

**Fig. 5:**
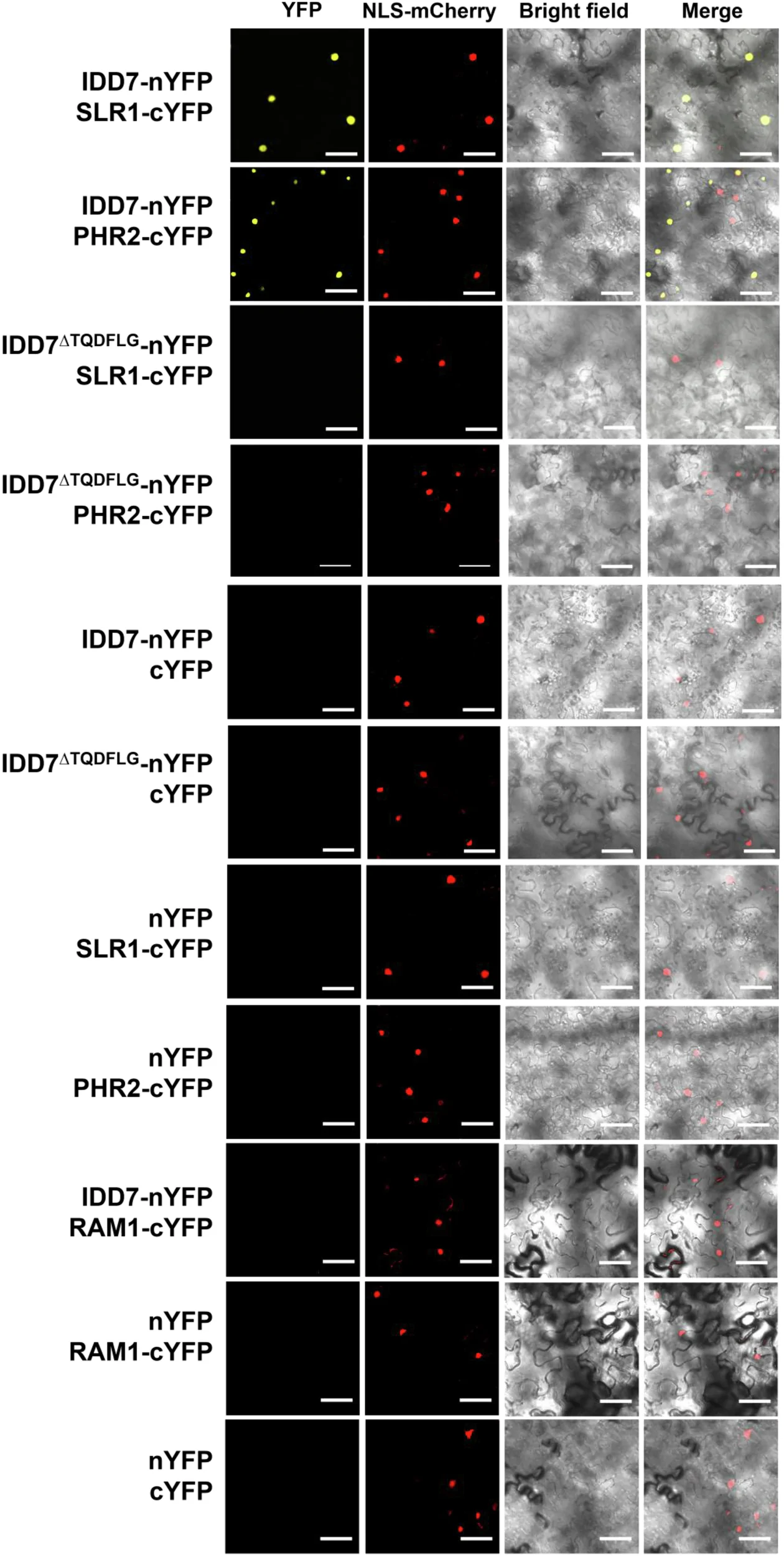
IDD7 may interact with SLR1 and PHR2 through the TQDFLG domain *in planta*. The interaction between IDD7 and IDD7 lacking the TQDFLG domain (IDD7^∆TQDFLG^) with SLR1, PHR2, or RAM1 *in N. benthamiana* leaves was demonstrated using a BiFC assay. The full-length CDSs without stop codons of *IDD7* and *IDD7* lacking the TQDFLG domain were fused to nYFP. The full-length CDSs without the stop codons of *SLR1*, *PHR2*, and *RAM1* were fused to cYFP. *N. benthamiana* leaves were coinfiltrated with *A. tumefaciens* strain *EHA105* containing the corresponding constructs. Combinations of nonfused nYFP or cYFP served as negative controls, while NLS-mCherry was utilized as a nuclear marker. Scale bars = 50 μm. Representative BiFC images are shown from three independent experiments with similar results.

Previous studies have shown that IDD transcription factors can interact with DELLA proteins in various species^23–25^. Since IDD7 and PHR2 both activate the *RAM1*, *WRI5a*, and *PT11* promoters (Supplementary Fig. 5), their potential interactions were also tested. BiFC assays revealed YFP signals in the nucleus when IDD7 was coexpressed with PHR2 or SLR1 but not with RAM1. No YFP signals were observed in combinations containing nonfused nYFP or cYFP (Fig. 5 and Supplementary Fig. 6a). These results showed that PHR2 and SLR1 interacted with IDD7 in the nucleus *in planta*. In addition, Co-immunoprecipitation (co-IP) assays in *N. benthamiana* further supported the physical association between IDD7 and SLR1 as well as between IDD7 and PHR2 in planta (Supplementary Fig. 6b). Both interactions were further confirmed in rice protoplasts using BiFC assays (Supplementary Fig. 6c), supporting its relevance in a homologous system. Although PHR2 and SLR1 interacted with each other, their interaction was noticeably weaker than that of either protein with IDD7 (Supplementary Fig. 6d). Moreover, the BiFC signal between PHR2 and SLR1 decreased in the presence of IDD7. However, SLR1 fusion proteins accumulated at lower levels under triple co-expression conditions. Thus, the reduced BiFC signal should be interpreted cautiously but suggests that IDD7 may affect the PHR2–SLR1 association (Supplementary Fig. 6d). Firefly luciferase complementation imaging (LCI) assays further validated these interactions (Supplementary Fig. 6e–g).

To identify key interaction domains, IDD7 truncations were tested. IDD7 contains an N-terminal NLS, four zinc fingers, and an ID domain, along with two conserved C-terminal domains—MSATALLQKAA and TQDFLG—based on its homology to AtIDD3^23^ (Supplementary Fig. 6h). YFP signals appeared when the C-terminal regions IDD7-C (287 a.a.) and IDD7-C2 (165 a.a.) were coexpressed with PHR2 or SLR1, suggesting that the TQDFLG domain might be essential (Supplementary Fig. 6i). Deletion of this domain (IDD7^ΔTQDFLG^) markedly reduced the interaction signals in the BiFC assay, indicating that the conserved C-terminal TQDFLG motif is important for the association of IDD7 with PHR2 and SLR1 (Fig. 5; Supplementary Fig. 6a and j). Immunoblot analysis confirmed the expression of all BiFC fusion proteins. Although most proteins accumulated at comparable levels, the SLR1-cYFP and IDD7^ΔTQDFLG^-nYFP signals appeared moderately reduced relative to the corresponding WT IDD7–SLR1 combination (Supplementary Fig. 6j). Therefore, BiFC fluorescence was interpreted only as relative signal intensity because it can be affected by protein accumulation and transient-expression variability. Consistently, in the LCI assay, deletion of this domain abolished the interaction with SLR1 and substantially reduced, but did not completely eliminate, the interaction with PHR2 (Supplementary Fig. 6e–g). These results support an important role for the conserved C-terminal TQDFLG motif in mediating IDD7 interactions with PHR2 and SLR1.

Finally, *SLR1* is constitutively expressed in mycorrhizal roots at the stage when *IDD7* is also highly expressed. However, *SLR1* expression was unaffected in both the mock and mycorrhizal roots of *idd7* mutants and complementation lines, indicating that IDD7 did not regulate *SLR1* expression (Supplementary Fig. 4f).

### IDD7 acts together with SLR1 and PHR2 to enhance downstream gene expression

**Fig. 6:**
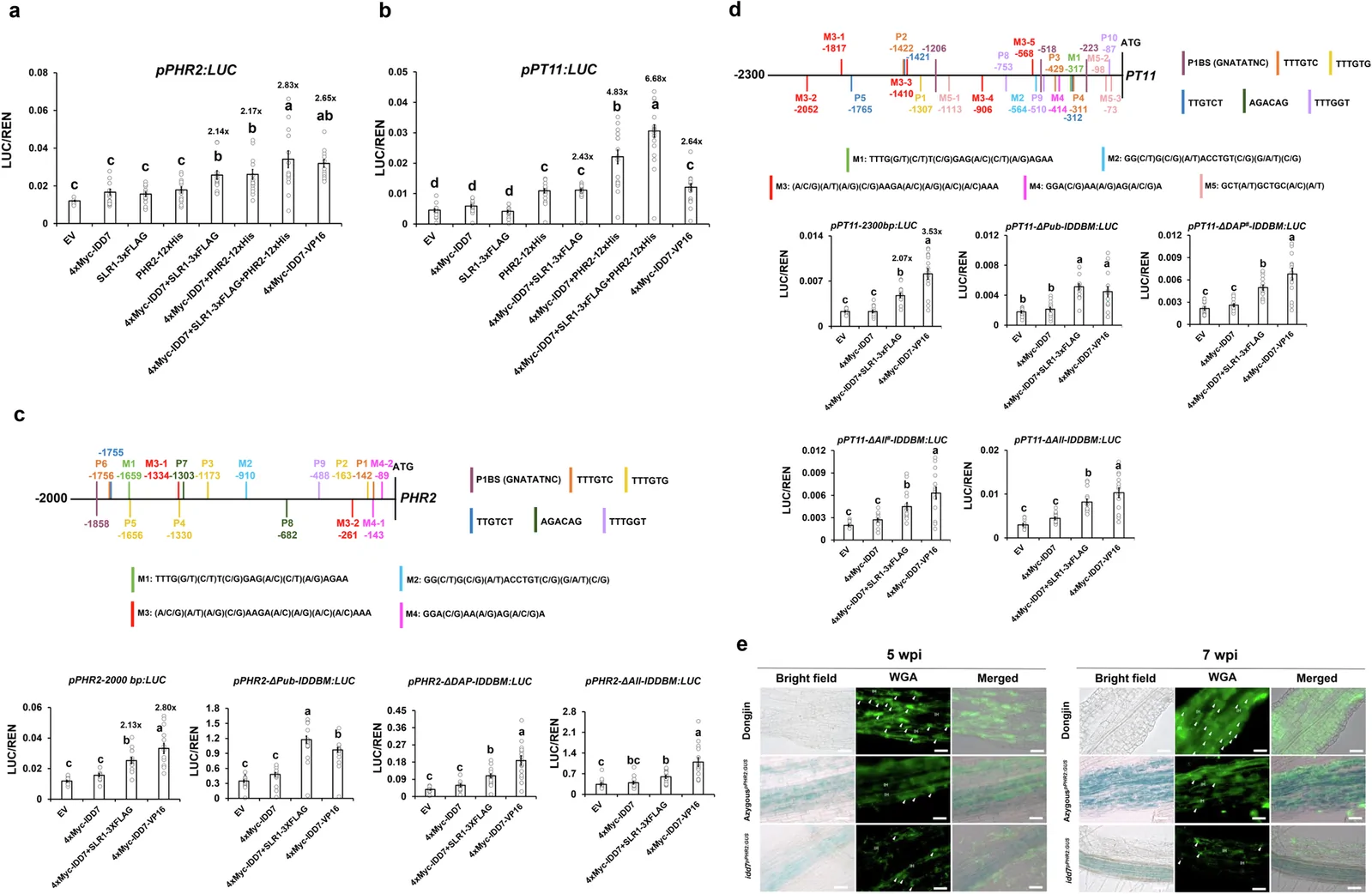
IDD7 activates *PT11* and *PHR2* through conserved IDD-binding motifs and is required for *PHR2* expression in arbuscule-containing cells. Promoter activities of *PHR2* and *PT11* were assessed using transient expression in *Nicotiana benthamiana*. **a**, **b** Reporter constructs contained the native *PHR2* (2000 bp) or *PT11* (3700 bp) promoters. **c** Sequential deletions of EMSA probe sequences containing published IDD-binding motifs (*ΔPub-IDDBM*), DAP-seq-identified motifs (*ΔDAP-IDDBM*), or all IDD-binding motifs (*ΔAll-IDDBM*) were introduced into the 2000-bp *PHR2* promoter, generating fragments of 1787, 1856, and 1706 bp, respectively. **d** Corresponding deletion series were generated for the *PT11* promoter, producing *ΔPub-IDDBM* (2109 bp), *ΔDAP*^*#*^*-IDDBM* (2132 bp; deleting DAP–seq motifs identified based on a 10-bp mismatch/gap tolerance, M1, M2, M3-1, M4, M5-1/2/3), *ΔAll*^*#*^*-IDDBM* (1990 bp; deleting all published and DAP-seq motifs identified based on 10-bp tolerance), and *ΔAll-IDDBM* (1908 bp; deleting all motifs above plus DAP-seq sites identified based on a 6-bp tolerance). EV denotes empty vector. Schematics (**c**, **d**) indicate the positions of published IDD-binding motifs (labeled by “P”), DAP–seq motifs (M1–M5), and P1BS elements. Relative fold activation compared with the EV control is indicated above the corresponding bars. Data are presented as mean ± SEM (*n* = 14 leaf disks from 14 independently infiltrated leaves per treatment, with one or two leaves per plant used for a given treatment). Different letters represent significant differences among the treatments (*P* < 0.05, one-way ANOVA followed by a least significant difference post hoc test); exact *P* values for the post hoc pairwise comparisons are provided in the Source Data. **e** GUS activity driven by the 2000-bp *PHR2* promoter in roots of Azygous and *idd7* plants. Bars = 50 μm. Bright-field and WGA overlays highlight arbuscules (arrowheads), and intraradical hyphae (IH). Similar patterns were observed in at least two independent lines.

To assess whether IDD7 acts together with SLR1 and PHR2 to regulate promoter activity, transactivation assays were conducted in *N. benthamiana* leaves using the *PT11*, *PHR2*, *RAM1*, *WRI5a, CYCLOPS*, and *CCaMK* promoters (Fig. 6a, b; Supplementary Fig. 7a–e). Like IDD7, SLR1 alone had little or no effect on most promoters, except for *CYCLOPS*. PHR2 alone activated *PT11*, *RAM1*, *WRI5a*, and *CYCLOPS* but repressed *CCaMK*, and did not activate its own promoter, even when a P1BS motif was present (Fig. 6c). IDD7 coexpression with SLR1 activated *PT11*, *PHR2*, *RAM1*, and *WRI5a*. Coexpression of IDD7 and PHR2 further increased promoter activity for several targets, including *PT11*, *RAM1*, and *CYCLOPS*, consistent with cooperative transcriptional regulation (Supplementary Fig. 7a, b, and d). This enhancement was not caused by increased PHR2 protein accumulation (Supplementary Fig. 7f) and was abolished when the P1BS motifs were deleted from the *PT11* promoter (*pPT11-ΔP1BS*) or mutated in the *RAM1* promoter (*pRAM1-mP1BS*) (Supplementary Fig. 7a and b). For the *pRAM1-mP1BS:LUC* and *pPT11-ΔP1BS:LUC* constructs, promoter activation required the simultaneous presence of both IDD7 and PHR2, whereas expression of either factor alone was insufficient. These observations are consistent with cooperative regulation in this transient assay, although the underlying mechanism remains unclear. For the *WRI5a* and *PHR2* promoters, different combinations of IDD7, SLR1, and PHR2 produced comparable activation, suggesting promoter-dependent differences in the effects of these combinations (Fig. 6a and Supplementary Fig. 7c). Notably, the highest activation of *PT11*, *PHR2*, *RAM1*, *WRI5a*, and *CYCLOPS* promoters was observed upon coexpression of IDD7, SLR1, and PHR2, indicating that these three factors together produced the strongest activity in this transient system (Fig. 6a, b; Supplementary Fig. 7b–d). Only IDD7-VP16 activated the *CCaMK* promoter, indicating that, under these experimental conditions, IDD7 requires additional cofactors for transcriptional activation rather than DNA binding (Supplementary Fig. 7e). The differential *pCCaMK:LUC* activities observed under various factor combinations further indicate that *CCaMK* promoter activity is subject to context-dependent regulation by these factors.

Further analysis intersected 115 AM-related DEGs with reduced expression in *idd7* vs. Azygous (Fig. 4b) with DEGs that were reduced in expression in the *osphr1/2-1/3* triple mutant (Shi et al. ^21^; Supplementary Fig. 7h and i), identifying 69 shared genes. These genes included nutrient transporters (e.g., *PT11*, *NPF4.5*, *AMT2;3*, *STR*) and TFs (*WRI5a*, *RAM1*, *CYCLOPS*), Thus, IDD7- and PHR2-dependent datasets overlap for a subset of AM-responsive genes. However, this overlap does not distinguish between direct coregulation, parallel regulation, or indirect effects, and therefore does not by itself demonstrate functional interaction between IDD7 and PHR2.

Interestingly, deletion of the TQDFLG domain resulted in autonomous IDD7 activity: unlike full-length IDD7, IDD7^ΔTQDFLG^ alone activated the *PT11* promoter. Its coexpression with SLR1 or PHR2 was similar to that of full-length IDD7 (Supplementary Fig. 7g). Together with the interaction assays, these results suggest that TQDFLG contributes to partner interaction while constraining the transcriptional activity of IDD7 in the absence of a coregulator (Supplementary Fig. 7g).

### IDD7 binds conserved motifs in the promoters of *PHR2* and *PT11*

Previous studies have identified potential binding sites for IDDs, including several reported IDD-binding motifs (Supplementary Fig. 8a)^23,26–34^. Motif analysis revealed a conserved but degenerate core sequence, most commonly centered on TTTGTG/C. Several related motif variants were identified within the 2-kb *PHR2* promoter (Supplementary Data 4). These candidate sites were designated P1–P9, and 24-bp EMSA probes containing each motif and its flanking sequence were designed (Supplementary Fig. 8a and Supplementary Data 4).

Electrophoretic mobility shift assays (EMSAs) revealed that MBP-IDD7, but not MBP alone, shifted all the tested probes, with variability in shift intensity across probes (Supplementary Fig. 8b). Competition with unlabeled WT probes abolished the shifts, whereas most mutated competitors exhibited substantially reduced or no competition, and most mutated probes themselves showed no detectable binding. P5 and P9 were exceptions, supporting the sequence specificity of IDD7 binding. Thus, IDD7 binds multiple related motif variants, including TTTGTG/C-containing elements, within the *PHR2* promoter (Supplementary Fig. 8b). Similarly, the 2.3-kb *PT11* promoter harbored multiple motif variants consistent with the IDD7-binding consensus (Supplementary Fig. 8c and Supplementary Data 4). EMSAs revealed that MBP-IDD7 bound most probes, although the quantity of shifted probe varied and was not strictly correlated with core motif sequence (Supplementary Fig. 8d). Mutated probes generally showed reduced or abolished binding and competition, confirming the sequence specificity of IDD7 binding, with only a few exceptions. Notably, residual binding was observed for the P10 mutant, likely reflecting the degenerate nature of IDD7-binding motifs and the contribution of flanking sequences. Sequence logo analysis indicated that, besides the AGACAG and TTTGGT core motifs, adjacent bases may also influence IDD7 binding, particularly an upstream A in AGACAG-containing probes and an upstream G in TTTGGT-containing probes (Supplementary Fig. 8e and f). Together, these results indicate that IDD7 recognizes a set of degenerate but related motifs within the *PT11* promoter.

### DAP-seq reveals IDD7-binding M1–M5 motifs

To identify additional IDD7-binding motifs, DNA affinity purification sequencing (DAP-seq) was performed, revealing more than 13,000 target loci shared between two replicates (Supplementary Fig. 9a and Supplementary Data 2). A total of 28.6% of these target loci were in promoter regions, with IDD7 binding enriched near transcription start sites (Supplementary Fig. 9b and c), although many peaks also appeared in exons (35.8%). Comparison of the DAP-seq dataset with 379 known AM-related genes^20,22^ and the *idd7* mutant-DEGs with reduced expression revealed 115 overlapping genes, representing potential IDD7 targets that require in vivo validation (Fig. 3b and Supplementary Fig. 9d). Among these genes, 13 were known AM genes (e.g., *PT11*, *NPF4.5*, *RAM1*, and *CCD8*), and IDD7 showed strong binding to the promoters of 10 (Supplementary Fig. 9f). Integrative Genomic Viewer (IGV) showed strong IDD7 binding at the *PT11* and *RAM1* promoters (Supplementary Fig. 9e). Motif enrichment analysis revealed M3, M4, and M5 as the top three motifs in the promoters of 27 AM-related IDD7 target genes, whereas M1 and M2 were most enriched in the promoters of 10 known AM genes (Supplementary Fig. 9g).

IDD7 coexpressed with SLR1 activated the promoter activity of *PHR2* (Fig. 6a). Although *PHR2* was not identified as an IDD7 target by DAP-seq, its promoter contained DAP-seq–identified motifs (M1–M4) with some mismatches (Supplementary Fig. 9h and Supplementary Data 5). M1 was identified using a four-base mismatch/gap tolerance, whereas M2–M4 were identified using a 6–10-base tolerance. EMSAs confirmed that IDD7 bound to these motifs, with mobility shifts generally reduced by excess unlabeled WT competitors. Mutations altered the mobility-shift patterns, although several mutant probes retained detectable shifts (Supplementary Fig. 9i), indicating that IDD7 could recognize these motifs on the *PHR2* promoter. Similarly, the *PT11* promoter contained motifs M1–M5 (Supplementary Fig. 9j and Supplementary Data 5). M3-2 to M3-5 were identified using a six-base mismatch/gap tolerance, whereas the other sites were identified using a 10-base tolerance. IDD7 bound to all probes except M3-3, and the mobility shifts were generally reduced by excess unlabeled WT competitors. For several probes, the corresponding mutant competitors were less effective, although this distinction was less evident for some weakly shifted probes. Mutations also altered the mobility-shift patterns, although several mutant probes retained detectable shifts. Notably, compared with the original probes, the M2, M4, and M5-2 mutations resulted in stronger mobility-shift signals (Supplementary Fig. 9k). Overall, these results support the conclusion that IDD7 recognizes representatives of all five motif classes in the *PT11* promoter. Although EMSAs demonstrate sequence-specific binding in vitro, these results do not establish in vivo occupancy, which will require future chromatin-based analyses.

AlphaFold3-based structural modeling and atomic interaction profiling^35^ predicted that IDD7 recognizes DNA through a bimodal mechanism engaging both grooves (Supplementary Fig. 9l). Arg179 in the ZF4 domain recognizes G-rich motifs by inserting into the major groove and forming specific hydrogen bonds with guanine bases. In contrast, Arg77 in the ZF1 domain penetrates the minor groove of AT-rich regions, sensing DNA shape and electrostatics. Additional residues stabilize the complex via non-specific interactions with the phosphate backbone.

### Essential motifs for IDD7-driven activation

To assess the contribution of IDD-binding motifs to promoter activation, we generated *PHR2* and *PT11* promoter variants lacking EMSA probe regions containing published motifs (*ΔPub-IDDBM*), DAP-seq-derived motifs (*pPHR2-ΔDAP-IDDBM*, in which IDD motifs identified based on a 4–10 bp mismatch/gap tolerance were deleted, and *pPT11-ΔDAP*^*#*^*-IDDBM*, in which motifs identified based on a 10 bp tolerance were deleted), or both motif classes (*pPHR2-ΔAll-IDDBM* and *pPT11-ΔAll*^*#*^*-IDDBM*) (Fig. 6c, d; Supplementary Figs. 8 and 9). This deletion strategy was used because mutation of individual motifs did not completely abolish IDD7 binding in EMSAs, suggesting residual binding or redundancy among nearby sequences. The *PHR2* variants showed different basal activities, likely because the deletions altered the remaining cis-regulatory elements (Fig. 6c). IDD7 with SLR1 activated the ΔPub-IDDBM and ΔDAP-IDDBM variants of both promoters, whereas the ΔAll-IDDBM promoter retained significant but reduced activation by IDD7 with SLR1 (Fig. 6c, d). These results indicate that the combined published and DAP-seq-derived regions contribute substantially to IDD7–SLR1-dependent activation of the *PHR2* promoter but are not solely responsible for this activity.

Because IDD7 with SLR1 still activated the *pPT11-ΔAll*^*#*^*-IDDBM* promoter, we generated a further deletion, *pPT11-ΔAll-IDDBM*, lacking EMSA probe regions containing all published and M1–M5-like motifs. IDD7 with SLR1 still activated this promoter, suggesting the presence of additional IDD7-binding or regulatory sequences. M6 and M7 were the fourth- and fifth-most enriched motifs among 27 AM-related candidate targets (Supplementary Fig. 9g), and IDD7 bound both corresponding *PT11* probes (Supplementary Fig. 9g, j and k). Because M6 and M7 were identified using a permissive sequence-matching threshold and lack a clearly defined short core motif suitable for targeted mutagenesis, only WT competitor assays were performed for these regions. Therefore, these assays demonstrate binding to the tested promoter fragments rather than specific recognition of the predicted M6 or M7 motifs. Future experiments will determine whether deletion of M6 and M7 abolishes IDD7–SLR1-dependent activation.

Surprisingly, IDD7–VP16 activated all deletion variants, indicating that transcriptional activation in this heterologous transient-expression system does not strictly depend on the predicted IDD-binding motifs. Instead, activation may involve endogenous *N. benthamiana* transcription factors, additional cis-regulatory elements within the promoter fragments, or other indirect mechanisms. Therefore, these assays demonstrate that IDD7 has transcriptional regulatory activity in a transient plant expression system but should not be interpreted as definitive evidence of motif-dependent direct regulation or in vivo promoter occupancy in rice roots.

### IDD7 drives PHR2 in arbuscule-containing cells

To assess the role of IDD7 in regulating *PHR2* expression, we examined GUS activity driven by a 2-kb *PHR2* promoter in Azygous and *idd7* mutant plants (Supplementary Fig. 2g). In Azygous roots, promoter–GUS activity was confined to arbuscule-containing cortical cells at 5 and 7 wpi (Fig. 6e). By contrast, in the *idd7* mutant, *PHR2* expression was restricted to the stele and completely absent from cortical cells, irrespective of arbuscule formation. These findings indicate that IDD7 is essential for *PHR2* expression in arbuscule-containing cells.

### *IDD7* expression depends on functional CSSP signaling but is not directly regulated by the CCaMK–CYCLOPS complex

To assess whether *IDD7* functions downstream of CSSP, phenotypic analyses were performed on *ccamk-1*, *cyclops-1*, and *cyclops-3* mutants^36^. These mutants showed reduced *RiITS1* expression and colonization (Supplementary Fig. 10a–d), with significantly lower *IDD7* transcript levels in AM-colonized roots, indicating that *IDD7* expression depends on intact CSSP signaling (Supplementary Fig. 10e and f). However, this dependence may reflect reduced colonization and/or impaired upstream signaling rather than direct transcriptional regulation by the CCaMK–CYCLOPS complex.

In addition, transactivation assays in *N. benthamiana* revealed that the coexpression of OsCYCLOPS and gain-of-function CCaMK^T263D^ (OsCCaMK^298-T263D^-NLS, abbreviated as CCaMK^T263D^) did not activate the *OsIDD7* or *OsRAM1* promoters (Supplementary Fig. 10g), whereas the *SlNF-YC3* promoter was activated under the same experimental conditions, confirming the activity of the effector constructs (Supplementary Fig. 10g)^37^.

## Discussion

*IDD7* was specifically activated in arbuscule-containing cortical cells at 5–7 wpi (Fig. 1c, d), consistent with a role in arbuscule development. This is supported by recent single-nucleus RNA-seq tracking in dicots, which reveals enrichment of IDD transcripts, such as *SlNUC* (*Solyc09g074780.3*), in colonized inner cortex cell clusters during active arbuscule development^38^. The *idd7* mutants showed reduced fungal colonization and stunted arbuscules (Figs. 2 and 3), similar to those of *Osram1* mutants but with stronger defects^9^. The reduced expression of *RAM1*, *WRI5a*, *FatM*, *RAM2*, and *STR1/STR2* (Fig. 4) and the enrichment of lipid biosynthesis genes according to GO/KEGG analyses (Supplementary Fig. 3e, f) indicated that IDD7 coordinated arbuscule maturation through lipid metabolism.

IDD7 functions as a higher-order regulator coordinating multiple transcriptional pathways during AM symbiosis. While *PHR2* represents a direct downstream component supported in planta, previous studies have shown that *phr2* mutants exhibit reduced colonization and smaller arbuscules^9^, supporting its role in arbuscule development. However, the *idd7* mutant displays broader and more severe defects, including arbuscule degeneration and hyphal septation (Fig. 3), indicating that IDD7 regulates additional pathways required for symbiotic maintenance. Our data support a model in which IDD7 regulates a subset of key regulators, including *PHR2*, *PT11*, and *RAM1* (Fig. 6a, b; Supplementary Figs. 7b and 9e), with *PHR2* regulation confirmed in planta (Fig. 6e) and additional targets supported by transient transactivation assays and DNA-binding analyses (Fig. 6b; Supplementary Figs. 7–9). The combined disruption of these pathways likely underlies the strong symbiotic defects observed in *idd7*.

IDD7 interacted with SLR1 to activate *PT11*, *RAM1*, *WRI5a*, and *PHR2* (Fig. 6a, b; Supplementary Fig. 7b and c), resembling the IDD–DELLA activation module in Arabidopsis^23^. MtDELLA1, expressed in vascular and endodermal tissues, promotes arbuscule formation by relocating to cortical cells^15,39^. Whether OsSLR1 exhibits a similar pattern remains to be determined. IDD7 also interacts with PHR2 and together additively activate the *PT11*, *RAM1*, and *CYCLOPS* promoters (Fig. 6b and Supplementary Fig. 7b and d), consistent with cooperative regulation. As GRAS proteins are generally not known to bind DNA directly, the activation of the *CYCLOPS* promoter by SLR1 alone likely occurs through interaction with endogenous transcription factors or other components of the transcriptional machinery. The requirement for both IDD7 and PHR2 to activate *pPT11-ΔP1BS:LUC* and *pRAM1-mP1BS:LUC* further supports cooperative regulation, although the underlying mechanism remains to be determined (Supplementary Fig. 7a and b). This is consistent with previous findings that PHR2 can cooperate with AM-induced transcription factors to regulate target gene expression^20^. Cotarget analysis revealed 69 AM-related genes that were shared between IDD7- and PHR2-dependent gene sets, including transporters (*PT11*, *NPF4.5*, *AMT2;3*, *STR*) and TFs (*WRI5a*, *RAM1*, *CYCLOPS*) (Supplementary Fig. 7h and i). However, this overlap does not distinguish between direct coregulation, parallel regulation, or indirect effects, and therefore does not by itself demonstrate a shared molecular mechanism. While PHR1/2/3 targets are enriched in redox, transport, and signaling functions^21^, IDD7 targets are associated with lipid/isoprenoid biosynthesis and receptor signaling (Supplementary Fig. 3e and f), suggesting complementary but distinct regulatory roles. *EXO70I*, *VAPYRIN*, *KIN2*, *ARK2*, and *DIP1* are genes with reduced expression in the *idd7* mutant but not in *phr2*-dependent datasets (Supplementary Fig. 7h and i), suggesting IDD7 has a distinct role in peri-arbuscular membrane development. *PHR2* is expressed in arbuscule-containing cells^9^ and was reduced in *idd7* and *idd7*^*EV*^ roots (Fig. 2g). Together with activation of the *PHR2* promoter by IDD7–SLR1 and IDD7–PHR2, the loss of cortical *PHR2* promoter activity in *idd7* supports a regulatory relationship in which IDD7 acts upstream of and also interacts with PHR2 (Fig. 6a, e). Similar relationships have been described for MtRAM1–MtRAD1^12^ and LjRAM1–LjWRI5a/b/c^7^.

Co-expression of IDD7, SLR1, and PHR2 resulted in the strongest activation of the *PT11* promoter (Fig. 6b), although other transcription factors, such as NF-YC3, are also likely to contribute to its regulation^40^. How these TFs coordinate to regulate *PT11* in arbuscule-containing cells remains to be explored. Notably, the rice *PT11* promoter cannot be activated in a dicot background^41^, while RAM1 and WRIs readily induce *PT4* in dicots^7^. These findings suggest that AM-specific phosphate transporter promoters in monocots and dicots rely on different transcription factor networks. Co-expression of IDD7, SLR1, and PHR2 also resulted in the strongest activation of the *PHR2, RAM1, WRI5a*, and *CYCLOPS* promoters (Fig. 6a, b and Supplementary Fig. 7b–d), supporting cooperative transcriptional regulation by these factors. However, these transient assays were performed in a heterologous *N. benthamiana* system and therefore likely represent only part of the regulatory mechanism operating in arbuscule-containing rice cells. Additional transcription factors, chromatin context, and AM-specific signaling are likely to contribute to the full induction of these genes during AM symbiosis. The conserved TQDFLG domain in IDD7 functioned as both a protein–protein interaction site and an autoinhibitory element (Fig. 5; Supplementary Figs. 6 and 7g), reminiscent of the Meis2 Hth domain^42^. The TQDFLG domain may therefore function as a regulatory switch that enables full transcriptional activation in the presence of cofactors during AM symbiosis.

The promoter deletion analyses indicate that the predicted IDD-binding motifs contribute to IDD7–SLR1-mediated activation of the *PT11* and *PHR2* promoters (Fig. 6c, d). However, the ability of IDD7–VP16 to activate deletion constructs suggests that activation in this transient-expression system does not strictly depend on these predicted motifs and may involve endogenous transcription factors, additional cis-elements, or other indirect mechanisms. Therefore, these transient assays should be interpreted as evidence of transcriptional regulatory activity rather than definitive evidence of motif-dependent regulation in rice. Previous studies identified 64 possible 3-bp target sequences as potential binding sites for C2H2-type zinc fingers, illustrating the sequence diversity recognized by this class of DNA-binding proteins^43^. In addition to base-specific contacts, phosphate groups and linker regions also contribute to zinc finger–DNA recognition^44^. Consistent with these features, structural modeling predicted that IDD7 combines Arg179-mediated recognition of G-rich sequences with Arg77-mediated recognition of AT-rich flanks (Supplementary Fig. 9l). This bimodal recognition mechanism may enable IDD7 to bind multiple related degenerate promoter motifs, including those present in the *PT11* and *PHR2* promoters.

*IDD7* expression depends on CCaMK and CYCLOPS signaling, as its induction is lost in *cyclops* and *ccamk* mutants (Supplementary Fig. 10). However, OsCYCLOPS and gain-of-function CCaMK did not activate the *IDD7* promoter, suggesting that its dependence on CCaMK–CYCLOPS signaling is indirect. Conversely, reduced *CCaMK* and *CYCLOPS* expression in *idd7* roots (Supplementary Fig. 4d and e) and altered promoter activities in transient assays (Supplementary Fig. 7d and e) suggest possible feedback regulation, although its relevance in rice roots remains unclear. Previous studies have confirmed that CYCLOPS and CCaMK are essential for arbuscule development in cortical cells^1,45,46^, supporting a model in which IDD7 contributes to the broader transcriptional network associated with CCaMK–CYCLOPS signaling during symbiosis.

**Fig. 7:**
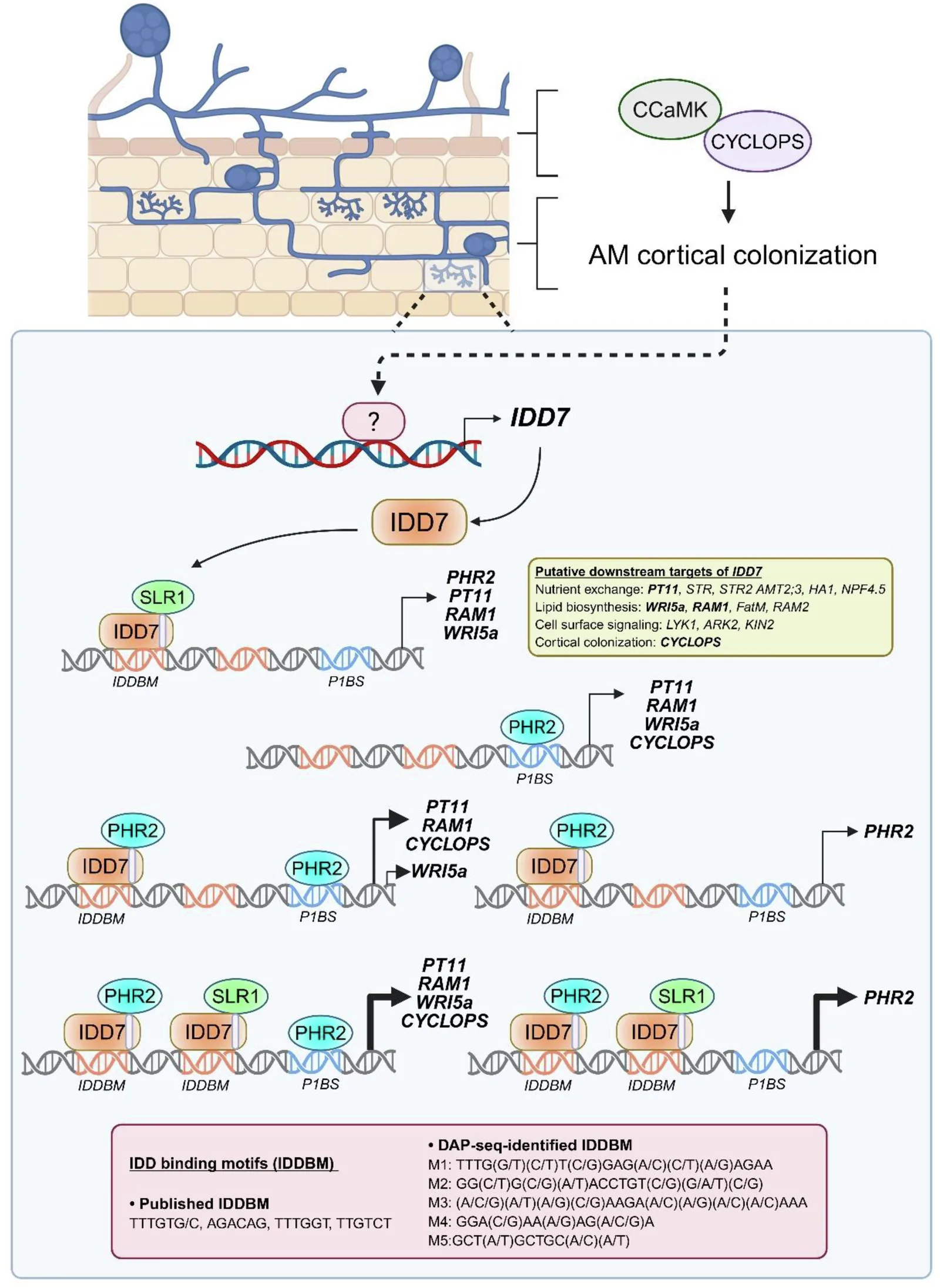
IDD7 coordinates transcriptional activation of AM symbiotic genes through interactions with SLR1 and PHR2. CCaMK and CYCLOPS signaling is essential for cortical colonization^36^. In arbuscule-containing cells, *IDD7* expression is likely activated by an unknown factor. IDD7 interacts with SLR1 and PHR2 via its conserved TQDFLG domain. PHR2 alone activates the *PT11*, *RAM1*, *WRI5a*, and *CYCLOPS* promoters but represses *CCaMK* and does not regulate its own promoter. Coexpression of IDD7 with SLR1 activated the promoters of *PT11*, *PHR2*, *RAM1*, and *WRI5a*. The combined expression of IDD7 and PHR2 further increased the promoter activity of *PT11*, *RAM1*, and *CYCLOPS*, indicating cooperative activation via P1BS and IDD-binding sites. The strongest activation of *PT11*, *PHR2*, *RAM1*, *WRI5a*, and *CYCLOPS* occurred with the coexpression of IDD7, SLR1, and PHR2, highlighting the role of IDD7 in enhancing their activities through binding to different activators. EMSAs confirmed that IDD7 binding to published and DAP-seq-identified motifs is required for promoter activation. RNA-seq analysis identified genes showing IDD7-dependent expression changes during AM symbiosis. Among these, *PT11*, *RAM1*, and *PHR2* represent candidate direct targets supported by IDD7 binding and transactivation assays, whereas other AM-related genes may be indirectly affected, potentially reflecting reduced colonization in *idd7* mutants. Created in BioRender. Yang, S. (2026) https://BioRender.com/otbqcoi.

In conclusion, CCaMK–CYCLOPS signaling is required for *IDD7* induction during AM symbiosis^36^, although the transcription factors that directly activate *IDD7* in arbuscule-containing cells remain unknown. Through its ability to bind multiple promoter motifs in vitro and regulate transcription in transient assays, IDD7 acts together with SLR1 and PHR2 to regulate the promoters of *PT11, PHR2, RAM1, WRI5a,* and *CYCLOPS*. Whereas *PHR2* regulation is supported in planta, regulation of the other candidate targets is supported by biochemical and transient expression assays. Consistent with this model, IDD7 interacts with both SLR1 and PHR2 across multiple systems, including BiFC assays in rice protoplasts and co-immunoprecipitation assays in *N. benthamiana*. Thus, IDD7 acts as a central integrator, coordinating transcriptional pathways required for proper arbuscule development and AM symbiosis (Fig. 7), although further validation of these interactions in rice root tissues and genome-wide identification of direct targets will refine this regulatory framework.

## Methods

### Plant growth conditions

Seeds of the rice (*Oryza sativa* cv. Nipponbare and Dongjin) and transgenic lines were sterilized with 70% ethanol for 4 min and 30 s, followed by 3% sodium hypochlorite for 30 min with gentle shaking and washing with sterile water. For the inoculation experiments, the seeds were germinated on half-strength Murashige and Skoog (1/2 MS medium (2.15 g MS basal salts/L, 10 g sucrose/L, pH 5.7–5.8 per liter)) with 0.8% agar in the dark for 5 days and then exposed to light for the next two days. The germination conditions included a 12-h/12-h day/night cycle at 30 °C/28 °C. To perform genotyping PCR before planting, leaves were collected to extract genomic DNA with a UniversAll Tissue Extraction/PCR Kit (Yeastern Biotech Co., Ltd., New Taipei City, Taiwan). The primers used for PCR genotyping are listed in Supplementary Table 2.

The seedlings were planted in autoclaved-sterilized sand without (mock) or with 200 spores of *Rhizophagus irregularis* (Ri) (IRBV’95 Mycorise ASP; Premier Tech Biotechnologies, Rivière-du-Loup, Quebec, Canada). The growth conditions included a 12-h/12-h day/night cycle at 30 °C/28 °C. Each plant was irrigated with 10 mL of distilled water during the first week, and starting from the second week, each plant was fertilized twice a week with half-strength Hoagland solution containing 25 μM KH_2_PO_4_.

### Plant materials

The T-DNA insertion *idd7* (3A-04543R) homozygous mutant in the genetic background of *Oryza sativa* ssp. Japonica cv. Dongjin was purchased from Postech, Korea^47^. For the *IDD7* complementation line (*idd7*^*IDD7*^), the constructs (*pIDD7*:IDD7 CDS and pB2GW7,0 empty vector (EV) with the IDD7 promoter) were transformed into the *idd7* mutant. The complementation lines in the T4 generation and the empty vector control in the T1 generation were used in the complementation assay. *ccamk-1* (NE1115), *cyclops-1* (NG0782), and *cyclops-3* (NC2713) mutant seeds were kindly provided by Prof. Uta Paszkowski and Dr. Jeongmin Choi from the University of Cambridge^36^. For the *IDD7* promoter: GUS (β-glucuronidase) lines, the construct (*pIDD7:GUS* and pHGWFS7,0 empty vector (EV)) was transformed into wild-type rice cv. Kittake. The *pIDD7:GUS* in the T2 generation and EV in the T2 generation were used. For the *PHR2* promoter: GUS lines, the construct (*pPHR2:GUS*) was transformed into Azygous and *idd7* mutants. The *pPHR2:GUS* in the T1 generation was used. The *IDD7* promoter: GUS lines were generated by the laboratory of Dr. Su-May Yu from the Institute of Molecular Biology, Academia Sinica. Other transgenic rice plants were generated by the Transgenic Plant Core Facility, ABRC, Academia Sinica.

### Gene construction

If the constructs were generated by LR recombination into GATEWAY destination vectors, Gateway™ LR Clonase II Enzyme Mix (Invitrogen, Carlsbad, CA, USA) was used.

For subcellular localization, the IDD7 coding sequence (1476 bp), with or without a stop codon, was cloned and inserted into the entry vector pCR™8/GW/TOPO™ and recombined into the destination vectors pH7WGF2.0 and pH7FWG2.0 to generate enhanced green fluorescent protein (EGFP) fusions at the N- and C-termini, respectively. The vector pCAMBIA1302 was used as a GFP control.

For the promoter::GUS constructs, a 2043-bp or 2000-bp fragment upstream of the *IDD7* or *PHR2* start codon was cloned and inserted into pHGWFS7.0 or pBGWFS7.0, respectively. For complementation, this promoter replaced the 35S promoter in pB2GW7.0 through the SpeI and SacI sites, and the *IDD7* CDS (1476 bp) in pCR8 was recombined into the pB2GW7.0-pIDD7 EV to yield the pB2GW7.0-pIDD7:IDD7 CDS.

For the dual-luciferase reporter (DLR) assays, the promoters of *PT11* (3700, 2300, 900, and 270 bp), *RAM1* (3000 bp), *WRI5a* (2500 bp), *PHR2* (2000 bp), *CYCLOPS* (2500 bp), *CCaMK* (2000 bp), *ABCB* (2000 bp), and *VAPYRIN* (2000 bp) amplified from Nipponbare genomic DNA were cloned and inserted into pGreenII 0800-LUC as reporters. EMSA probe sequences containing the motifs described below were deleted from each construct. The following deletion constructs were directly synthesized into pGreenII 0800-LUC: *pPHR2-ΔPub-IDDBM* (1787 bp; deleting promoter regions corresponding to EMSA probes (hereafter referred to as “EMSA probe regions”) that encompass published IDD-binding motifs P1–P9 in Supplementary Fig. 8a), *pPHR2-ΔDAP-IDDBM* (1856 bp; deleting EMSA probe regions encompassing DAP-seq-identified motifs identified based on a 4–10 bp mismatch/gap tolerance: M1, M2, M3-1, M3-2, M4-1, M4-2 in Supplementary Fig. 9h), *pPHR2-ΔAll-IDDBM* (1706 bp; deleting all EMSA probe regions described above), *pPT11-ΔPub-IDDBM* (2109 bp; deleting EMSA probe regions encompassing published motifs P1–P5 and P8–P10 in Supplementary Fig. 8c), *pPT11-ΔDAP*^*#*^*-IDDBM* (2132 bp; deleting EMSA probe regions encompassing DAP-seq motifs identified based on a 10-bp tolerance: M1, M2, M3-1, M4, M5-1/2/3 in Supplementary Fig. 9j), *pPT11-ΔAll*^*#*^*-IDDBM* (1990 bp; deleting all EMSA probe regions described above), *pPT11-ΔAll-IDDBM* (1908 bp; deleting all EMSA probe regions described above plus additional EMSA probe regions encompassing DAP-seq motifs M3-2, M3-3, M3-4, and M3-5 identified based on a 6-bp tolerance in Supplementary Fig. 9j). These deleted regions correspond to EMSA probes, and deletion was performed because mutation of individual motifs did not fully abolish IDD7 binding in EMSA assays. *pPT11-ΔP1BS* (3660 bp) and *pRAM1-mP1BS* (3000 bp), in which two P1BS motifs (GNATATNC) in the *RAM1* promoter were mutated to mP1BS (TNCGCGNA), were directly synthesized into pGreenII 0800-LUC.

For effector constructs, all CDSs (e.g., CYCLOPS [2,262 bp]) were cloned into pCR™8/GW/TOPO™ and transferred to the pMDC32 vector. IDD7–VP16 was generated by fusing the *IDD7* CDS (1476 bp, without a stop codon) to the VP16 activation domain amplified from pBI221-35S-GAL4DB-VP16. The 4×Myc–IDD7 and 4×Myc–IDD7–VP16 were generated in pGWB518 via LR recombination. PHR2–3×FLAG (1278 bp) and SLR1–3×FLAG (1875 bp) were cloned and inserted into pMpGWB111 and transferred to pENTR™/D-TOPO™. PHR2–12×His (1,373 bp) was constructed by cloning the *PHR2* CDS (1278 bp) into pMAL-c5v, followed by frame correction by overlapping PCR and reinsertion into pMDC32 using the PacI and AscI sites. Gain-of-function CCaMK298–T263D–NLS construct was generated using NEBuilder® HiFi DNA Assembly (New England Biolabs, Ipswich, MA, USA) following the design in previous publications^37,48^. *4xMyc-IDD7*^*ΔTQDFLG*^ were generated by ligating two CDS fragments using NEBuilder® HiFi DNA Assembly.

For BiFC assays, CDSs without stop codons of *IDD7* (1476 bp), *IDD7-C* (858 bp), *IDD7-C2* (492 bp), *IDD7*^*ΔTQDFLG*^ (1458 bp), *PHR2* (1278 bp), *SLR1* (1875 bp), and *RAM1* (2316 bp) were cloned and inserted into pENTR™/D-TOPO™ and subcloned and inserted into pUBC-cYFP or pUBC-nYFP.

For luciferase complementation imaging (LCI) assays, CDSs of *IDD7*, *IDD7*^*ΔTQDFLG*^, *PHR2*, *SLR1*, and *RAM1* were cloned and inserted into pCAMBIA1300-NLuc or -CLuc under the CaMV 35S promoter by restriction–ligation cloning.

For EMSA, the *IDD7* CDS (1476 bp, without a stop codon) was cloned and inserted into pMAL-c5v for expression as an N-terminal maltose-binding protein (MBP) and C-terminal 12×His fusion under lac operator control.

For the co-IP assay, EYFP was amplified from pEarleyGate 101 and cloned into the pCR™8/GW/TOPO entry vector. The inserts were subsequently transferred into the destination vector pEarleyGate 100 using LR Clonase II enzyme mix. The pCR8-IDD7 (without stop codon) and pCR8-SLR1 (without stop codon) constructs were then recombined into pEarleyGate 101 using LR recombination. The primers used for construct generation are listed in Supplementary Data 3.

### Rice protoplast preparation and transformation

The sterilized Nipponbare seeds were germinated under 1/2 MS (with B5 vitamin) with 0.8% (w/v) agar. The seedlings were grown at 30 °C in the dark for 10 days. A bundle of rice plants, including shoots and roots, was cut into ~0.5-mm strips. The strips were incubated in 30 mL of enzyme solution (1.5% [w/v] Cellulase RS [Yakult], 0.75% [w/v] macerozyme R10 [Yakult], 10 mM MES, pH 5.6, 0.6 M mannitol, 10 mM CaCl_2_, and 0.1% [w/v] BSA) for 4 h in the dark with gentle shaking and filtered through 70-mm nylon mesh to release protoplasts. The protoplasts were collected by centrifugation at 200×*g*, washed once with W5 solution (154 mM NaCl, 125 mM CaCl_2_, 5 mM KCl, and 2 mM MES), and resuspended with MMg solution (0.4 M mannitol, 15 mM MgCl_2_, and 4 mM MES, pH 5.7). For the BiFC assay, 5 μg of the nYFP construct, 5 μg of the cYFP construct, and 2 μg of NLS-mCherry construct were mixed with 100 mL of protoplasts. Then, 110 mL of freshly prepared polyethylene glycol solution (40% [v/v] PEG4000, 0.2 M mannitol, and 100 mM CaCl_2_) was added and mixed gently, and the mixture was incubated at room temperature for 15 min. After incubation, 440 mL of W5 solution was added, and protoplasts were collected by 200×*g* centrifugation. The supernatant was removed, and the protoplasts were resuspended gently in 0.2 mL of WI solution (20 mM KCl, 0.5 M mannitol, and 4 mM MES, pH 5.7) and incubated in the dark for 16 h.

### Transient expression in *N. benthamiana*

Constructs were transformed into *Agrobacterium tumefaciens* strain EHA105 for BiFC and into GV3101 for LCI. The leaves of 3- to 4-week-old *Nicotiana benthamiana* plants were infiltrated with the corresponding strains. For BiFC, the leaf disks were imaged using an LSM 780 confocal microscope (Carl Zeiss AG, Oberkochen, Germany). The excitation/emission wavelengths were 514/516–560 nm for YFP, 488/493–577 nm for GFP, and 594/591–674 nm for mCherry. The images were processed using ZEN Black software v7.0 (Carl Zeiss AG, Oberkochen, Germany). Empty pUBC-cYFP and pUBC-nYFP served as negative controls for BiFC. The GFP vector and NLS-mCherry vector were provided by Dr. Ya-Yun Wang (Department of Life Science, NTU) and Dr. Ke-Qiang Wu (Institute of Plant Biology, NTU), respectively. BiFC images were acquired using fixed microscope settings, with YFP images captured at a fixed gain (645–650) and brightness uniformly adjusted to 70% in PowerPoint. YFP fluorescence was quantified as a relative BiFC signal rather than an absolute measure of interaction strength. Because the NLS-mCherry marker was not encoded on the same plasmid as the BiFC constructs, it was used to identify successfully transformed nuclei but not for direct normalization of YFP intensity. After confirming nuclear localization via mCherry fluorescence, YFP intensity was measured using ImageJ. JS (v0.6.0) following background fluorescence subtraction, and only nuclei with detectable mCherry fluorescence were included in the analysis^49^. LCI assays followed a published protocol^50^. NLuc and CLuc constructs were coexpressed in *N. benthamiana* leaves for 48 h. D-luciferin (100 mM stock, Abcam, Cambridge, UK) was diluted to 1 mM and infiltrated into leaves. For qualitative imaging, leaves were dark-adapted for 7 min and imaged using a UVP ChemStudio Plus (Analytik Jena US, Upland, CA, USA). For quantitative assays, 6-mm leaf disks were incubated with 1 mM luciferin for 7 min in a 96-well white plate, and luminescence was measured immediately using an Infinite® 200 PRO reader (Tecan, Männedorf, Switzerland)^51^.

### Transactivation assay using the DLR system

The effector (pMDC32) and reporter (pGreenII 0800-LUC) constructs were transformed into *A. tumefaciens* strains GV3101 and GV3101, respectively, containing the pSoup vector^52^. The leaves of 3- to 4-week-old *N. benthamiana* plants were infiltrated with a solution composed of agrobacteria carrying the respective constructs. The luciferase activity was assayed using the Dual-Luciferase Reporter® Assay System (DLR) (Promega, Madison, WI, USA). The relative luciferase activity (LUC/REN) was calculated as the ratio of firefly luciferase activity (LUC) to Renilla luciferase activity (REN). To analyze 4xMyc-IDD7, 4xMyc-IDD7-VP16, SLR1-3xFLAG, PHR2-12xHis, nLUC/cLUC fusion proteins, and cYFP/nYFP fusion protein levels in *N. benthamiana*, 24 leaf disks (1 cm diameter) were collected 4 days post-infiltration and frozen in liquid nitrogen. Samples were ground using a BAXIT tissue grinder, and total proteins were extracted with 200 µl buffer (100 mM Tris–HCl, pH 7.8, 4 M urea, 5% SDS, 15% glycerol, protease inhibitors). After heating (95 °C, 5 min) and centrifugation (13,500×*g*, 5 min), supernatants were analyzed by SDS–PAGE and immunoblotting. Proteins were transferred to PVDF membranes, blocked in PBST with 5% milk, and incubated overnight at 4 °C with primary antibodies: anti-Myc (M4439-100UL), anti-FLAG (F1804-50UG) (both 1:1000; Sigma-Aldrich, St. Louis, MO, USA), anti-His (1:5000; HIT001M, BIOMAN, New Taipei City, Taiwan), anti-nYFP (1:2000; AS11 1776, Agrisera, Vännäs, Sweden), anti-cYFP (1:10000; AS11 1775, Agrisera, Vännäs, Sweden), anti-n/c-LUC (1:4000; L0159, Sigma-Aldrich, USA), and anti-actin (1:4000; AS132640, Agrisera, Vännäs, Sweden). After PBST washes, membranes were incubated for 1 h with HRP-conjugated anti-mouse (1:4000; E-AB-1001, Elabscience, Wuhan, China) or anti-rabbit (1:10,000; ab6721, Abcam, Cambridge, UK) antibodies, and signals were detected using Opti-ECL (BIOMAN) on an iBright CL750 system (Invitrogen, Carlsbad, CA, USA).

### Co-IP assay

The indicated constructs were transformed into *Agrobacterium* GV3101 and transiently co-expressed in tobacco leaves by Agrobacterium-mediated infiltration. After 2 days, tobacco leaves were harvested and ground in liquid nitrogen. The IDD7-PHR2 protein pairs were extracted using a Tris-based protein extraction buffer (50 mM Tris–HCl, pH 7.4, 150 mM NaCl, 10% glycerol, 0.5% NP-40, 1 mM DTT, 1 mM PMSF, and 1× protease inhibitor cocktail (Roche, Basel, Switzerland), while 50 µM MG132 was added to the same buffer for the extraction of the IDD7–SLR1 protein pairs. Protein extracts were incubated with GFP-Trap beads (ChromoTek, Munich, Germany) at 4 °C for 4 h. The beads were washed twice with Tris wash buffer (50 mM Tris–HCl, pH 7.4, 150 mM NaCl, 10% glycerol, and 0.1% NP-40). The immunoprecipitated proteins and input protein extracts were heated at 95 °C for 10 min in SDS sample buffer (25 mM Tris–HCl, pH 6.8, 2% SDS, 5% glycerol, 12.5 mM EDTA, 0.5% β-mercaptoethanol, and 0.1% (w/v) bromophenol blue). Proteins were separated by SDS–PAGE and detected using anti-GFP (1:10,000; ab290-50UL, Abcam, Cambridge, UK), anti-FLAG (1:4000; F1804-50UG, Sigma-Aldrich, St. Louis, MO, USA), anti-MYC (1:2500; M4439-100UL, Sigma-Aldrich, St. Louis, MO, USA), and anti-His (1:5000; HIT001M, Bioman, New Taipei City, Taiwan) antibodies. After washing with TBST buffer (20 mM Tris, pH 7.6, 150 mM NaCl, 0.1% Tween 20), the membranes were incubated with HRP-conjugated secondary antibodies, including anti-mouse (1:10,000; E-AB-1001, Elabscience, Wuhan, China) or Clean-Blot IP Detection Reagent (1:1000; 21230, Thermo Fisher, MA, USA). Signals were visualized using the iBright™ imaging system (Invitrogen).

### AM fungal colonization quantification

Trypan blue staining of the collected root sample was performed to quantify mycorrhizal colonization under a microscope with a modified procedure as described previously^53^. The root samples were observed under a Primo Star light microscope (Carl Zeiss AG, Oberkochen, Germany).

### Promoter–GUS assay and WGA staining

Spatial expression of the *IDD7* promoter was examined by GUS staining followed by wheat germ agglutinin (WGA)–Alexa Fluor 488 staining (Invitrogen, Carlsbad, CA, USA), following a published protocol^54^. For the promoter‒GUS assay, the root samples were imaged under bright field (BF) on an Olympus BX51 fluorescence microscope (Olympus Corporation, Tokyo, Japan), allowing visualization of both GUS signals and WGA-derived green fluorescence marking fungal structures (excitation/emission 485/495–540 nm). To assess arbuscule development and cross-sectional area, roots were stained with WGA and imaged using confocal microscopy (TCS SP8; Leica Camera AG, Wetzlar, Germany, and LSM780; Carl Zeiss AG, Oberkochen, Germany) at 488/495–540 nm. Images of WGA–AF488–labeled arbuscules were acquired, and the arbuscule area was quantified using LAS X and ImageJ. JS (v0.6.0).

### RNA extraction, cDNA synthesis, qRT–PCR

The RNA extraction, cDNA synthesis, and RT-qPCR were performed following a previously described protocol^55^. The primers used for qRT‒PCR or RT‒PCR are listed in Supplementary Table 3.

### High-throughput RNA-seq analysis

Mycorrhizal roots of the Azygous and *idd7* mutants were harvested at 5 and 7 wpi and frozen in liquid nitrogen immediately. RNA was extracted using previously described methods, and the same RNA batch was used for qRT‒PCR and transcriptome analysis. Quality control, library preparation, differentially expressed gene (DEG) analysis, and GO and KEGG enrichment analyses were performed by BIOTOOLS Co., Ltd (New Taipei City, Taiwan). RNA quality and integrity were assessed using a Qsep100 DNA/RNA Analyzer (BiOptic Inc., New Taipei City, Taiwan), and quantification was performed using a SimpliNanoÊ—Biochrom Spectrophotometer (Biochrom, MA, USA). Samples with >1 µg RNA (OD260/280 = 1.69–2.15; RIN ≥ 6.7) were used for library construction. After purification and adapter ligation, libraries were sequenced on an Illumina NovaSeq 6000 platform (Illumina, Inc., San Diego, USA) to generate 150 bp paired-end reads at a depth of ~6 Gb per sample.

Raw sequencing reads in FASTQ format were subjected to adapter trimming and quality assessment using FastQC and MultiQC, after which RNA sequences were filtered with Trimmomatic (version 0.38) to ensure data integrity. The filtered reads were subsequently mapped to the *Oryza sativa* reference genome IRGSP1.0 (https://www.ncbi.nlm.nih.gov/datasets/genome/GCF_001433935.1/) by HISAT2 (version 2.1.0)^56,57^. Mapped sequences were counted by featureCounts (version 2.0.0)^58^. The relation log expression (RLE) gene expression normalization approach was adopted, and DESeq2 (version 1.26.0) was used for standardized differential gene analysis^59,60^. The DEG threshold was a |fold-change| ≥ 2 and adjusted *p*-value < 0.05, which was obtained by Benjamini and Hochberg’s approach to control the false discovery rate (FDR)^61^.

Gene Ontology (GO) and KEGG pathway^62,63^ enrichment analyses based on the obtained DEGs were performed by clusterProfiler (version 3.14.3)^64^. Web-based conversion of MSU (The Rice Genome Annotation Project, http://rice.plantbiology.msu.edu) to RAP (The Rice Annotation Project, https://rapdb.dna.affrc.go.jp) was performed before Venn diagram analysis using ID convertor (https://rapdb.dna.affrc.go.jp/converter/). A Venn diagram was produced by Bioinformatics & Evolutionary Genomics (https://bioinformatics.psb.ugent.be/webtools/Venn/). The heatmap was constructed using the pheatmap R package (1.0.12) and is presented on the cloud platform provided by BIOTOOLS Co., Ltd (New Taipei City, Taiwan).

### DAP-Seq experiments and data analysis

Rice (*Oryza sativa* ssp. japonica cv. Nipponbare) plants inoculated with *Rhizophagus irregularis* were grown under a 12-h light/dark cycle at 30/28 °C. Roots were harvested at 7 weeks postinoculation with >90% colonization. Genomic DNA was extracted using a CTAB-based protocol, treated with RNase A, fragmented to ~250 bp using a Covaris ultrasonicator, purified with AMPure XP beads, and used to construct libraries with the NEBNext® Ultra II DNA Library Prep Kit (New England Biolabs, Ipswich, MA, USA). HaloTag–IDD7 and HaloTag–GFP (control) were expressed using the TNT® SP6 Wheat Germ Cell-Free System (Promega, Madison, WI, USA), purified with MagneTM Halo Tag® Beads (Promega, USA), and incubated with the DNA library for 1 h. Bound DNA was eluted, amplified, and sequenced on an Illumina NovaSeq platform (150-bp paired-end, ~6 Gb per sample). Reads were trimmed with fastp (v0.23.2)^65^, assessed with FastQC^66^, mapped using BOWTIE2^67^, and normalized and visualized with DEEPTOOLS^68^, and peaks were identified by MACS2^69^. Significant binding sites were identified using a *q*-value cutoff of 0.05. IDD7-enriched peaks relative to GFP were obtained from two technical replicates (Rep A and Rep B), each comprising three biological replicates derived from root genomic DNA isolated from two plants per sample. Peak overlap and annotation were performed using Bedtools^70^, and motif discovery was performed by MEME^71^. Illumina sequencing and data analysis were performed by Genomics, BioSci & Tech Co. (New Taipei City, Taiwan).

### Protein extraction and purification

For EMSAs, the target CDS was cloned into pMAL-c5v to generate an N-terminal MBP and C-terminal 12×His fusion, expressed in *E. coli* BL21. Cultures were grown in LB with carbenicillin (100 µg ml−¹) at 37 °C to OD₆₀₀ > 0.4, induced with 0.1 mM isopropyl-b-D-thiogalactopyranoside (IPTG), and incubated at 16 °C for 20 h. Cells were lysed by sonication (Misonix, Farmingdale, NY, USA) in buffer (50 mM Na₂HPO₄, 150 mM NaCl, 10% glycerol; pH ~ 7), and the supernatant was clarified and filtered (Merck KGaA, Darmstadt, Germany). Proteins were purified using amylose resin (Cytiva, Marlborough, MA, USA) on an ÄKTAprime plus system (GE Healthcare, Chicago, USA), eluted with 20 mM maltose, further purified by heparin chromatography (Bio-link, Shanghai, China), and desalted with HiTrap resin (GE Healthcare, Chicago, USA).

### Electrophoretic mobility shift assay

Probes located on the *PHR2* and *PT11* promoters were synthesized and modified by FAM (fluorescein) at the 5’-end. Probes containing putative IDD binding sites from publications (TTTGTG/C, AGACAG, TTTGGT, and TTGTCT)^26,27,72^ were flanked by 9 bp on both the 5’ and 3’ ends. To generate the mutated probes, T was swapped with G, and C with A, among the six nucleotides designated as the putative IDD binding site. Probes containing IDD7 binding sites identified through DAP-seq and MEME analysis were flanked by 3–7 bps on both the 5’ and 3’ ends to reach a total length of 24 bp. To generate the mutated probes, T was swapped with G, and C with A, among the nucleotides that are conserved from the MEME analysis. The sequences of the labeled probe, competitor (unlabeled probe), and mutated probe are listed in Supplementary Data 4 and 5. The solution containing the forward and reverse sequences of the probe was incubated at 95 °C to prevent self-annealing. The mixture, consisting of 2 μg of purified protein and 40 ng of probe, was incubated in a binding system (3 μl of lysis buffer, with 0.5× TBE added to 15 μl) at room temperature for 30 min. The mixture was then resolved by electrophoresis on a 10% native PAGE gel.

### In silico promoter analysis

The promoter sequences starting from ATG were obtained from the Phytozome 13 database (https://phytozome-next.jgi.doe.gov/). The promoter sequences were analyzed with Plant Promoter Analysis Navigator 4.0 (PlantPAN 4.0; http://plantpan.itps.ncku.edu.tw/plantpan4/index.html)^73^ to locate P1BS, P1BS-like, and published IDD binding motifs. To locate the DAP–seq-identified IDD-binding motifs (M1–M5), promoter sequences were analyzed using SnapGene to identify similar DNA sequences, allowing one mismatch or gap/deletion every 6–10 bases. To identify the M1 motif on the *PHR2* promoter and the M6/M7 motifs on the *PT11* promoter, one mismatch or gap/deletion was allowed for every four bases.

### Statistical analysis

The number of biological replicates and the number of plants for each biological replicate are specified in the figure legends. Data were analyzed using Microsoft Excel 365 and are presented as the mean ± standard error of the mean (s.e.m.). The statistical significance of the differences between the control and experimental groups was assessed using one-sided or two-sided Welch’s *t*-tests, as specified in the corresponding figure legends, performed using Microsoft Excel 365 or through one-way ANOVA followed by a least significant difference (LSD, *p* < 0.05) post hoc test conducted using R Studio software (v4.4.1) and the agricolae package (v1.3-7). No adjustment for multiple comparisons was applied to the LSD post hoc tests. Exact *p*-values are provided in the figures or Source Data, as appropriate, and the statistical tests used for individual analyses are specified in the corresponding figure legends.

### Reporting summary

Further information on research design is available in the Nature Portfolio Reporting Summary linked to this article.

## Supplementary information

Supplementary Information

Description of Additional Supplementary Files

Supplementary Data 1

Supplementary Data 2

Supplementary Data 3

Supplementary Data 4

Supplementary Data 5

Reporting Summary

Transparent Peer Review file

## Data Availability

The RNA-seq and DAP-seq datasets generated during this study have been deposited in the NCBI Gene Expression Omnibus (GEO) under accession numbers GSE326810 and GSE327762, respectively. All other data supporting the findings of this study are available within the paper and its Supplementary Information. The source data underlying the figures in this study have been deposited in Figshare under DOI 10.6084/m9.figshare.32514456^74^.
