# Supplementary material for: OsIDD7 integrates signaling networks for arbuscular mycorrhizal symbiosis
Source: Nat Commun. 2026 Aug 31;17:10378. doi: 10.1038/s41467-026-77162-3 (PMC13627655; doi:10.1038/s41467-026-77162-3)
Supplement: Supplementary file 1 [file 41467_2026_77162_MOESM1_ESM.pdf]

Supplementary Fig.1

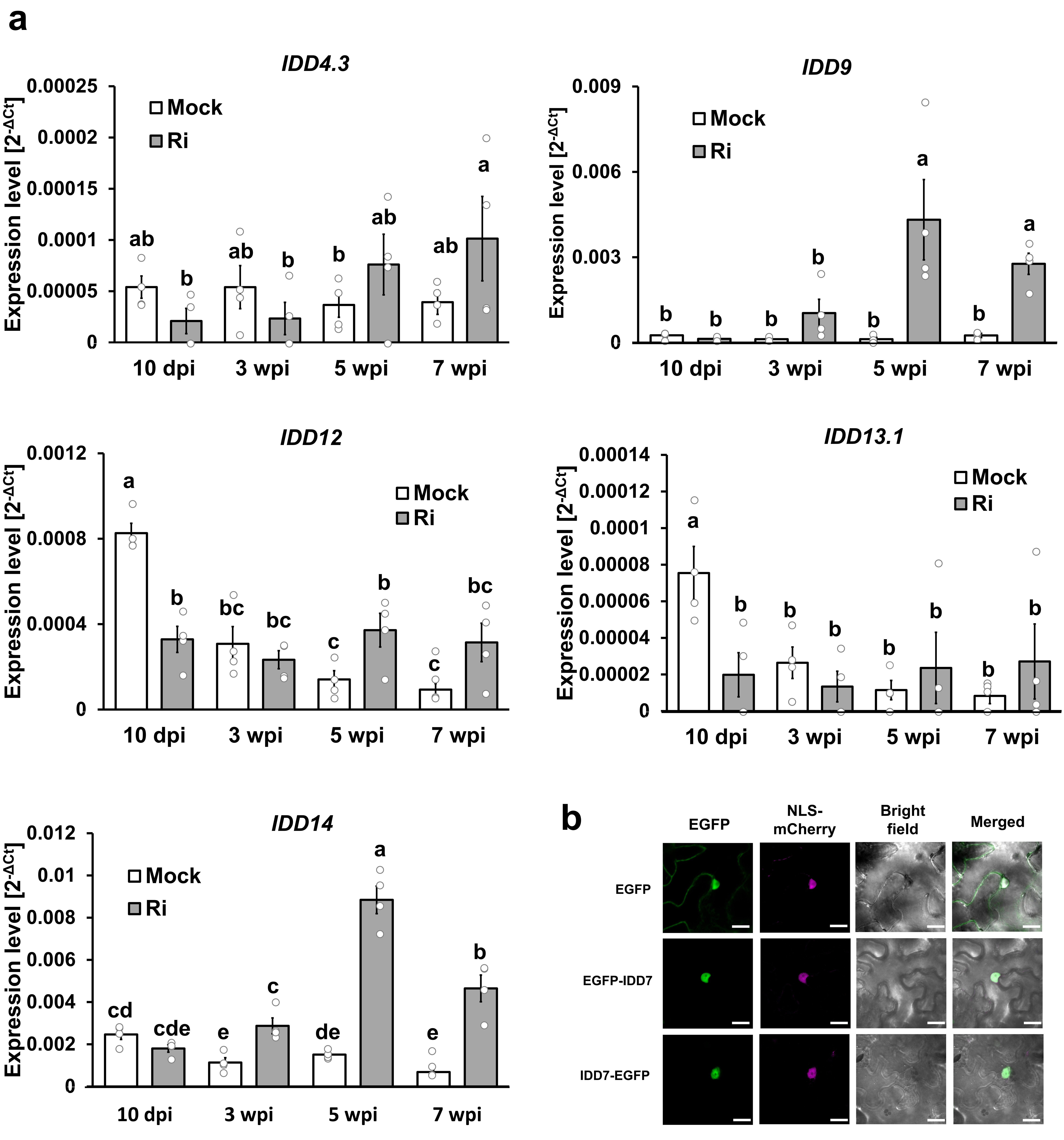

Supplementary Figure 1. Expression pattern of rice *IDDs* and subcellular localization of IDD7. a Gene expression pattern of *IDD4.3, 9, 12, 13.1* and *14*. Seven-day-old rice seedlings (*O. sativa* ssp. *japonica* cv. Nipponbare) were inoculated without (mock) or with AM fungi (*R. irregularis*, Ri) and grown under 25 μM Pi condition. Plants were harvested at 10 days post-inoculation (dpi) and 3, 5 and 7 weeks post inoculation (wpi). Gene expression was analyzed in the roots and normalized to *CYCLOPHILIN2* (*CYC2*) ($2^{-\Delta Ct}$). Data are presented as mean ± SEM. Each biological replicate consisted of root and shoot samples collected from the same plants, with samples pooled from 5, 4, 3, and 2 independently grown plants at 10 dpi, 3, 5, and 7 wpi, respectively. Sample sizes were n = 4 biologically independent pooled samples for roots and shoots at each time point. Different letters represent significant differences between the treatments ($P < 0.05$, one-way ANOVA followed by a least significant differences post hoc test). Exact P values for the indicated comparisons are provided in the Source Data. **b** Subcellular localization of EGFP-IDD7 and IDD7-EGFP protein in the leaves of *N. benthamiana.* Scale bars =20 μm. NLS-mCherry was used as a nuclear marker. Representative images are shown from two independent experiments with similar results.

Supplementary Fig.2

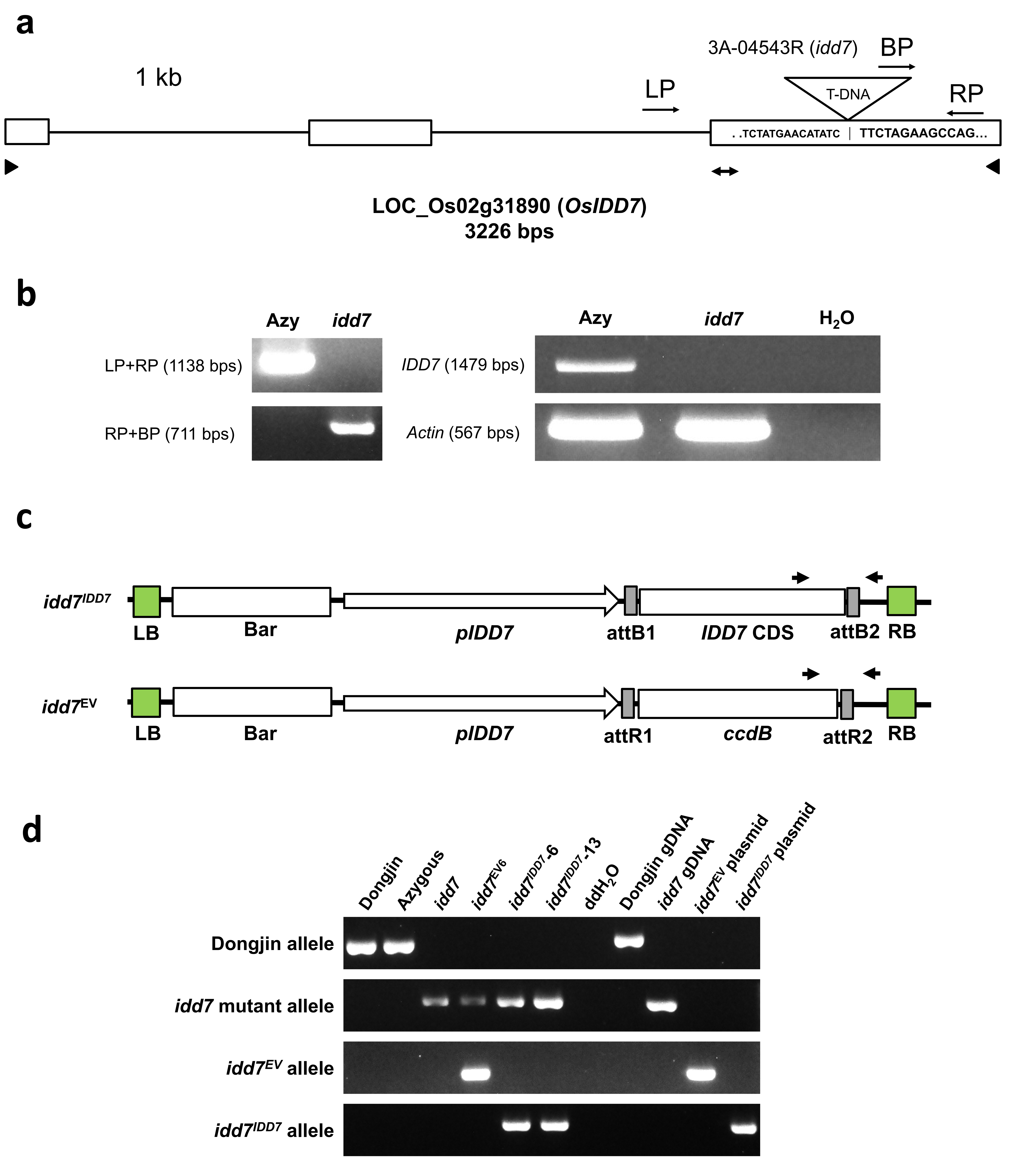

Supplementary Fig.2 (continued)

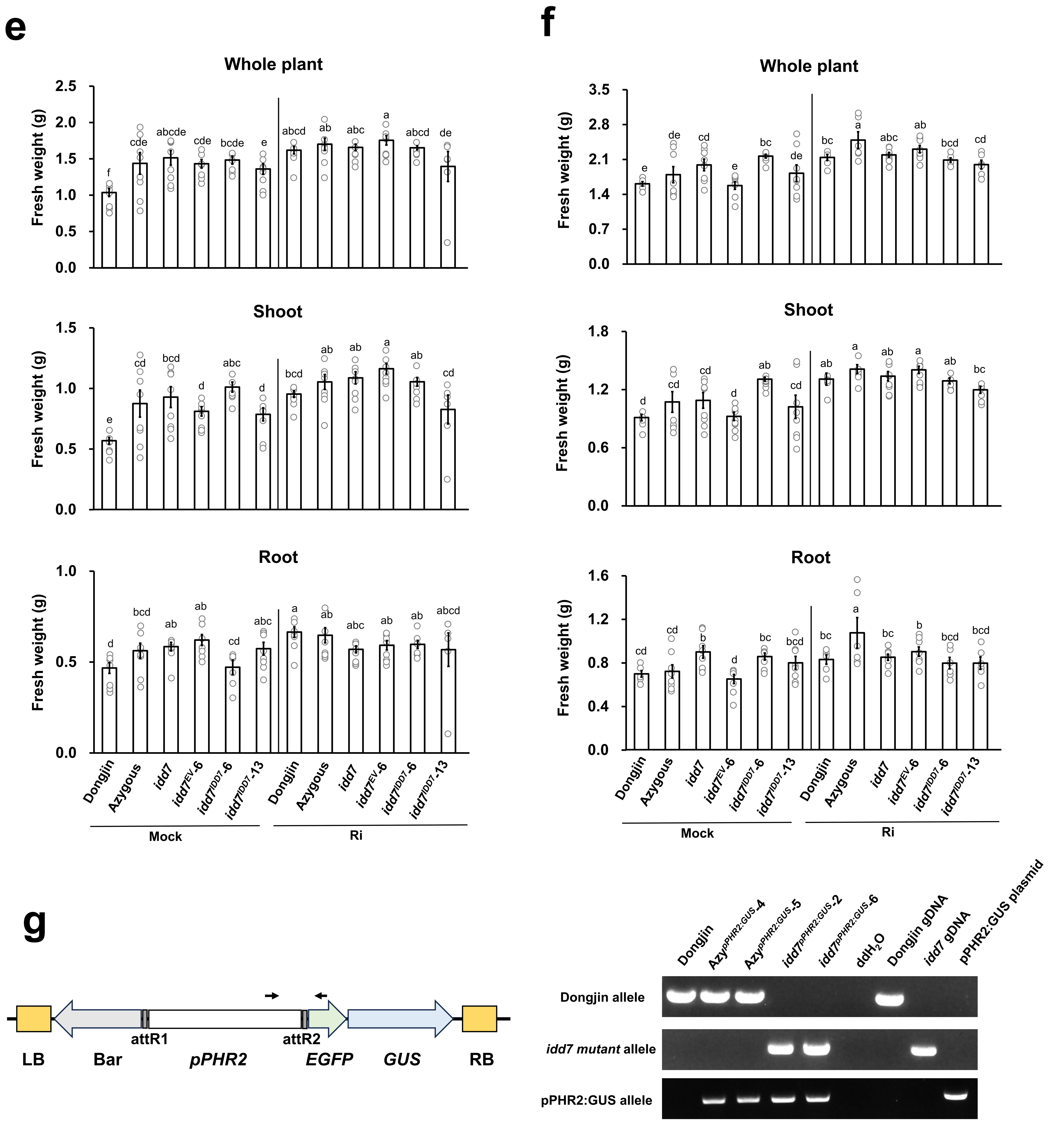

Supplementary Figure 2. Analysis of the *idd7* knockout mutant and $idd7^{IDD7}$ complementation lines, and genotyping PCR of *pPHR2:GUS* lines. a The gene structure of *IDD7*. The white rectangles and black lines represent exons and introns, respectively. The white triangle represents the T-DNA insertion site. The primers for genotyping PCR, RT–PCR and qRT–PCR are indicated with arrows, arrowheads and bidirectional arrows, respectively. **b** The results of genotyping PCR (left panel) and RT–PCR (right panel) of the *idd7* mutant (*idd7*). Azy (Azygous): segregated wild type from the *idd7* heterozygous mutant. **c** Schematic representations of construct structures for $idd7^{EV}$ and $idd7^{IDD7}$ complementation lines. LB and RB, the left and right borders, respectively; attB1, attB2, attB4, attR1 and attR2, the att sites for LR-recombination in cloning; Bar and ccdB, Basta resistant gene and DNA gyrase poison as selection marker during rice and *E. coli* transformation, respectively. White arrow, promoter sequence; black arrow, genotyping PCR primers. **d** PCR genotyping of the *idd7* mutant (*idd7*), *IDD7* complementation lines ($idd7^{IDD7}$) and their corresponding empty vector-transformed plants ($idd7^{EV}$). $idd7^{EV}$ plasmid: pB2GW7,0 *pIDD7* EV; $idd7^{IDD7}$ plasmid: pB2GW7,0 *pIDD7:IDD7* CDS. **e** Fresh weight of whole plants, shoots and roots at 5 weeks postinoculation (wpi) and **f** 7 wpi. Control plants (Dongjin and Azygous), the *idd7* T-DNA knockout mutant (*idd7*), the *idd7* mutant transformed with EV ($idd7^{EV}$) (T1 generation) and two complementation lines referred to as the *idd7* mutant transformed with the *pIDD7:IDD7* CDS construct ($idd7^{IDD7}$) (T4 generation) were inoculated without (Mock) or with the AM fungus *R. irregularis* (Ri), grown under 25 μM Pi conditions and harvested at 5 and 7 wpi. Data are presented as mean ± SEM. Each biological replicate consisted of roots pooled from one independently grown plants. For panel e, n = 6 biologically independent replicates for Mock $idd7^{IDD7}$*-6* and Ri $idd7^{IDD7}$*-13*; n = 8 biologically independent replicates for all other groups. For panel f, n = 6 biologically independent replicates for Mock Dongjin, Ri Dongjin, Ri Azygous, Ri $idd7^{IDD7}$*-6*, and Ri $idd7^{IDD7}$*-13*; n = 8 biologically independent replicates for all other groups. Different letters represent significant differences ($P < 0.05$, one-way ANOVA followed by a least significant difference post hoc test); exact P values for the post hoc pairwise comparisons are provided in the Source Data. **g** Schematic representations of construct structures and PCR genotyping for $Azy^{pPHR2:GUS}$ and $idd7^{pPHR2:GUS}$ lines. Black arrow, genotyping PCR primers. Representative genotyping PCR and RT-PCR results from two independent experiments are shown; similar results were obtained in both experiments.

Supplementary Fig.3

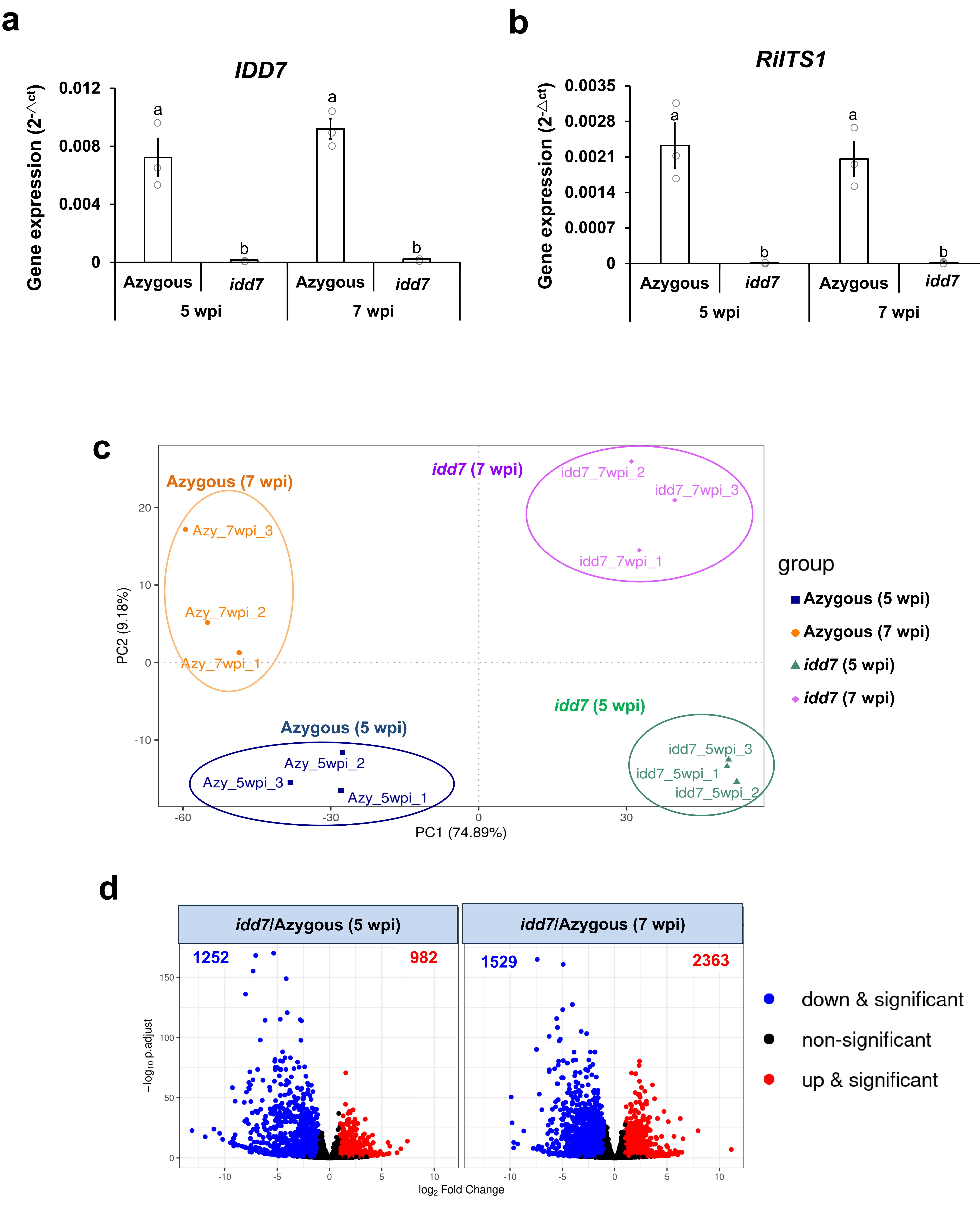

Supplementary Fig.3 (continued)

e

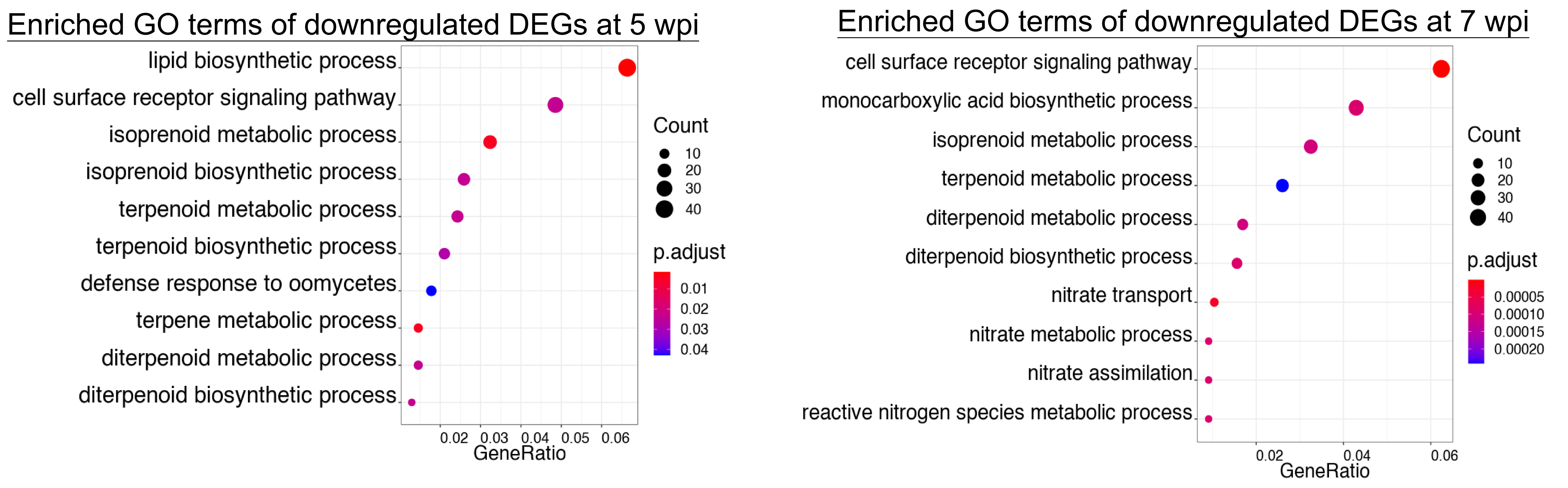

f

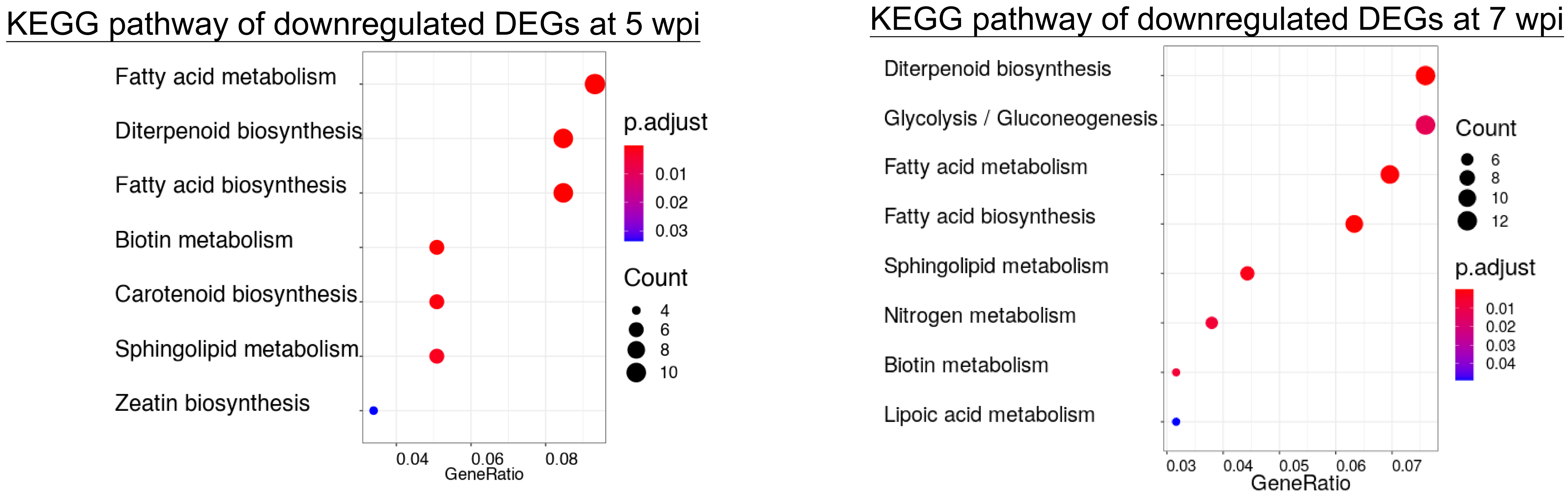

Supplementary Figure 3. Transcriptome profiling reveals IDD7-dependent regulation of nutrient, lipid, and signaling pathways. a *IDD7* expression, **b** *RiITS1* expression, **c** Principal component analysis (PCA) scatter plot based on the DESeq2 normalized counts representing the RNA-seq profiles of the indicated samples. **d** Volcano plot showing the distribution of DEGs. **e** The top 10 most significantly enriched Gene Ontology (GO) terms in biological processes of downregulated DEGs (*idd7* vs. Azygous) ranked by gene count at 5 weeks postinoculation (wpi) and 7 wpi. **f** KEGG pathway enrichment analysis of the downregulated DEGs (*idd7* vs. Azygous) at 5 wpi and 7 wpi. Azygous and *idd7* mutants were inoculated with the AM fungus *R. irregularis* (Ri), grown under 25 μM Pi conditions and harvested at 5 and 7 wpi. Gene expression in the roots was analyzed by qRT–PCR and normalized to that of *Cyclophilin2* (*CYC2*) ($2^{-\Delta Ct}$). Data are presented as mean ± SEM. Each biological replicate consisted of roots pooled from three independently grown plants. For panel a-b and RNA-seq, n = 3 biologically independent replicates. Different letters represent significant differences ($P < 0.05$, one-way ANOVA followed by a least significant difference post hoc test); exact P values for the post hoc pairwise comparisons are provided in the Source Data. The gene ratio on the x-axis represents the percentage of significant genes over the total number of genes in the GO terms or KEGG pathways.

Supplementary Fig.4

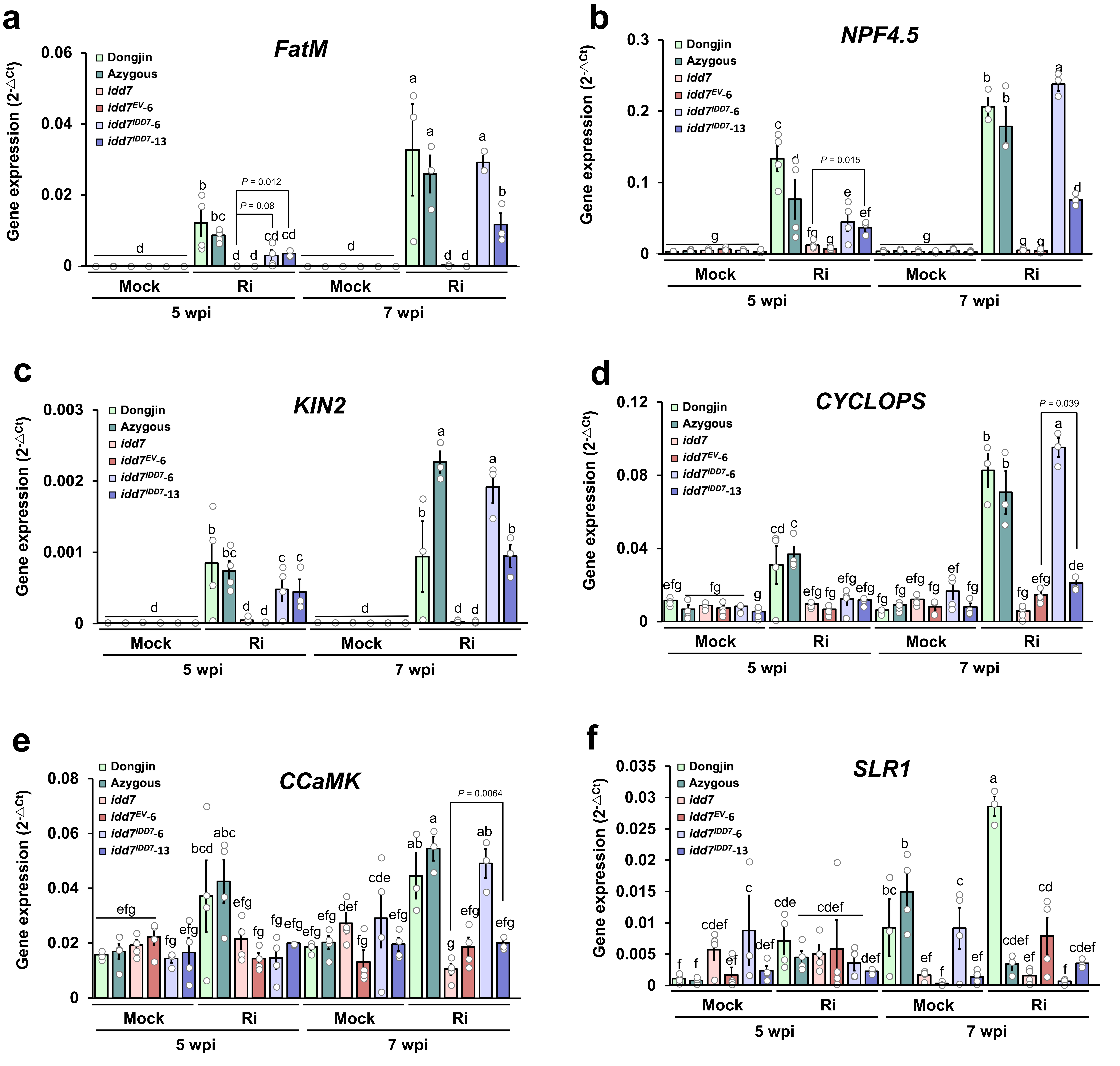

Supplementary Figure 4. Expression of AM-related genes in *idd7* knockout mutant and *idd7*^{IDD7} complementation lines. **a** *FatM* expression; **b** *NPF4.5* expression; **c** *KIN2* expression; **d** *CYCLOPS* expression; **e** *CCaMK* expression; **f** *SLR1* expression in the roots of different transgenic rice plants. Control plants (Dongjin and Azygous, the segregating wild type of the *idd7* mutant), the *idd7* T-DNA knockout mutant (*idd7*), the *idd7* mutant transformed with EV (*idd7*^{EV}) (T1 generation) and two complementation lines referred to as the *idd7* mutant transformed with the *pIDD7:IDD7* CDS construct (*idd7*^{IDD7}) (T4 generation) were inoculated without (Mock) or with the AM fungus *R. irregularis* (Ri), grown under 25 μM Pi conditions and harvested at 5 and 7 weeks postinoculation (wpi). Gene expression in the roots was analyzed by qRT–PCR and normalized to that of *Cyclophilin2* (*CYC2*) ($2^{-\Delta Ct}$). Data are presented as mean ± SEM. Each biological replicate consisted of roots pooled from two independently grown plants. For panels a–f, n = 3 biologically independent replicates for 5wpi Mock *idd7*^{IDD7}*-6*, 5wpi Ri *idd7*^{IDD7}*-13,* 7wpi Mock Dongjin, and 7wpi Ri Dongjin, Azygous, *idd7*^{IDD7}*-6*, and *idd7*^{IDD7}*-13*; n = 4 biologically independent replicates for all other groups. Different letters represent significant differences (P < 0.05, one-way ANOVA followed by a least significant difference post hoc test); exact P values for the post hoc pairwise comparisons are provided in the Source Data. For the indicated comparisons with the *idd7* mutant, exact P values are shown in the figure and were determined using one-sided Welch's t-tests.

Supplementary Fig.5

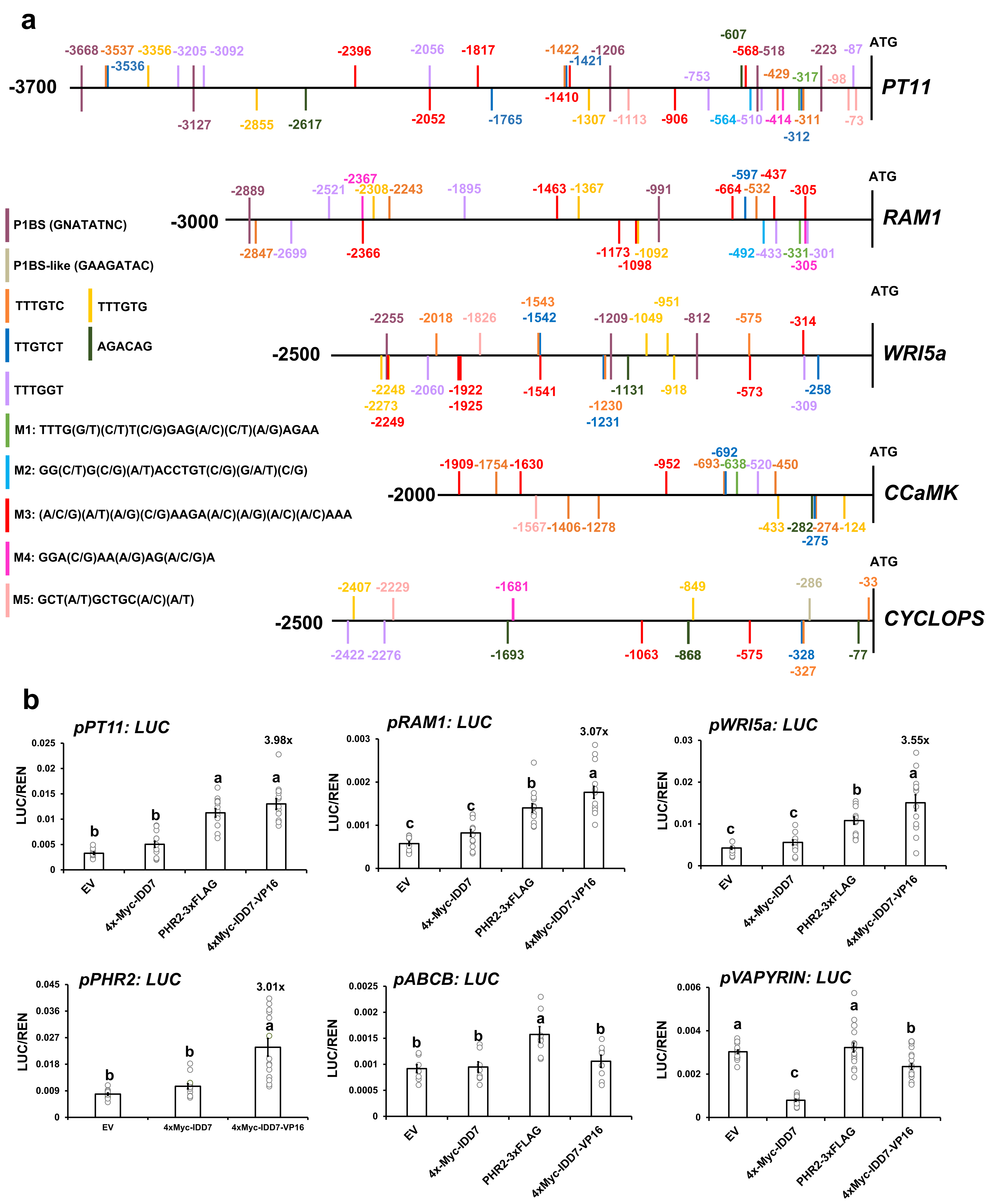

Supplementary Figure 5. Transactivation assays of candidate target promoters by IDD7-VP16. a Schematic representations showing the locations of published IDD binding sites (TTTGTC/G, TTGTCT, TTTGTG, AGACAG, and TTTGGT), DAP-seq-identified motifs (DAP-IDDBM, M1–M5), and P1BS/P1BS-like motifs on the promoters. **b** Dual-luciferase reporter (DLR) assay in *N. benthamiana* leaves transiently coexpressing the reporter construct containing the following *promoter*: LUC (firefly luciferase) and p35S (Renilla luciferase) with effector constructs, including EV (empty vector), 4xMyc-IDD7, PHR2-3xFLAG, and 4xMyc-IDD7-VP16. Relative fold activation compared with the EV control is indicated above the corresponding bars. Data are presented as mean ± SEM (n = 8 independently infiltrated leaf discs for the *ABCB* promoter, n = 21 for the *VAPYRIN* promoter, and n = 14 for all other groups). Each leaf disc represents an independently infiltrated leaf, with one or two leaves per plant used for a given treatment. Different letters represent significant differences among the treatments ($P < 0.05$, one-way ANOVA followed by a least significant difference post hoc test); exact P values for the post hoc pairwise comparisons are provided in the Source Data.

Supplementary Fig.6

a

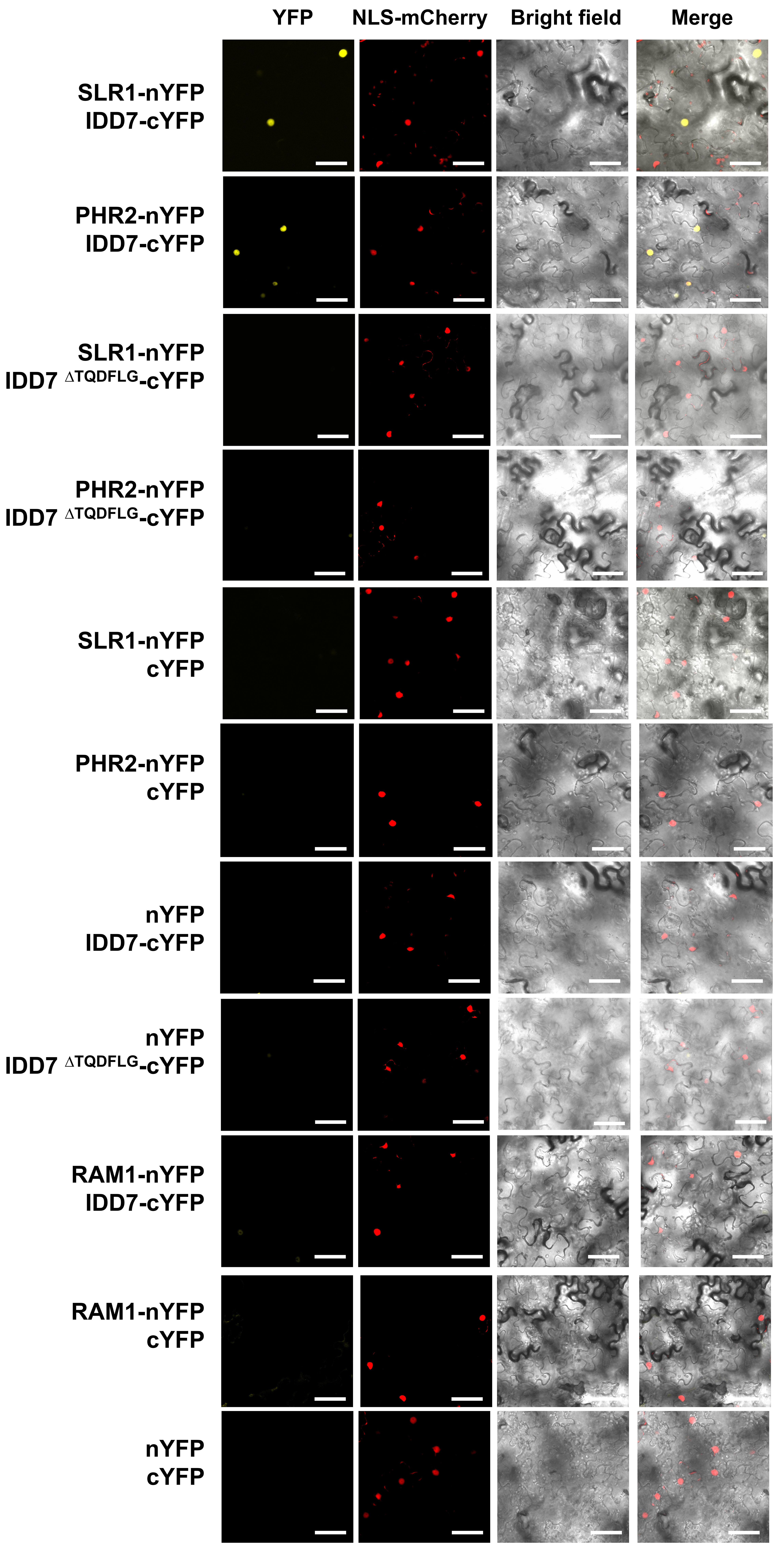

Supplementary Fig.6 (continued)

b

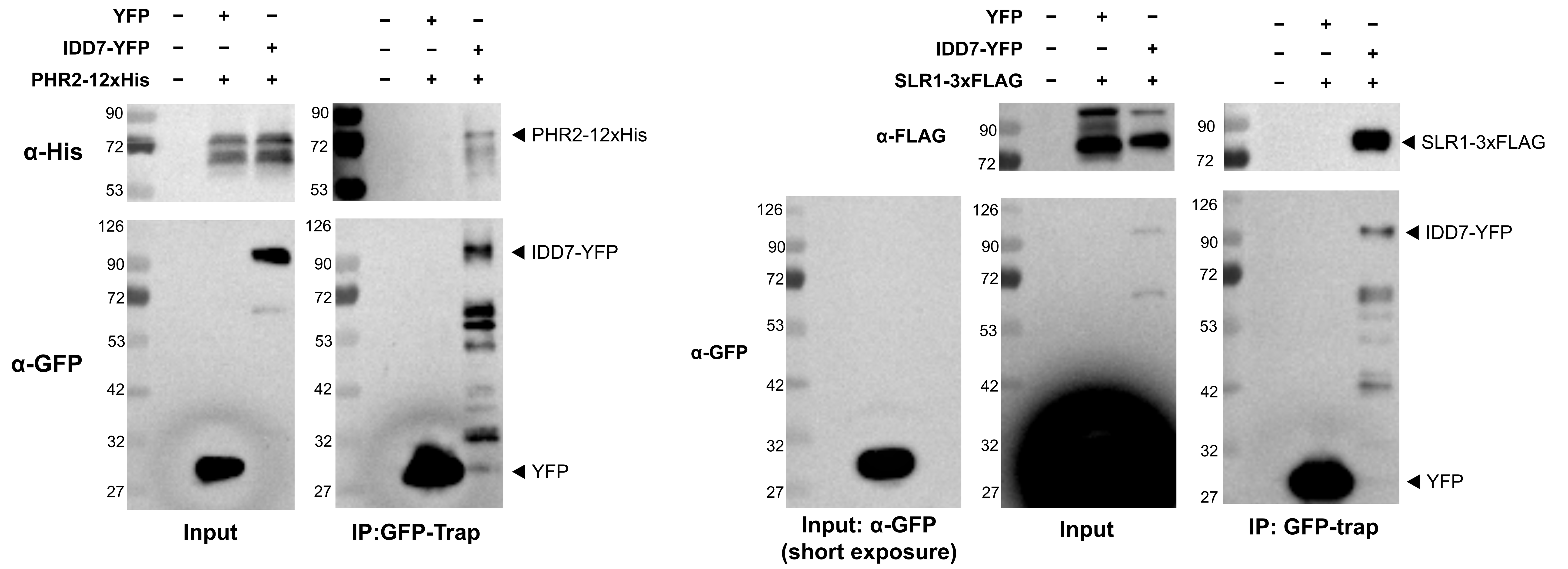

c

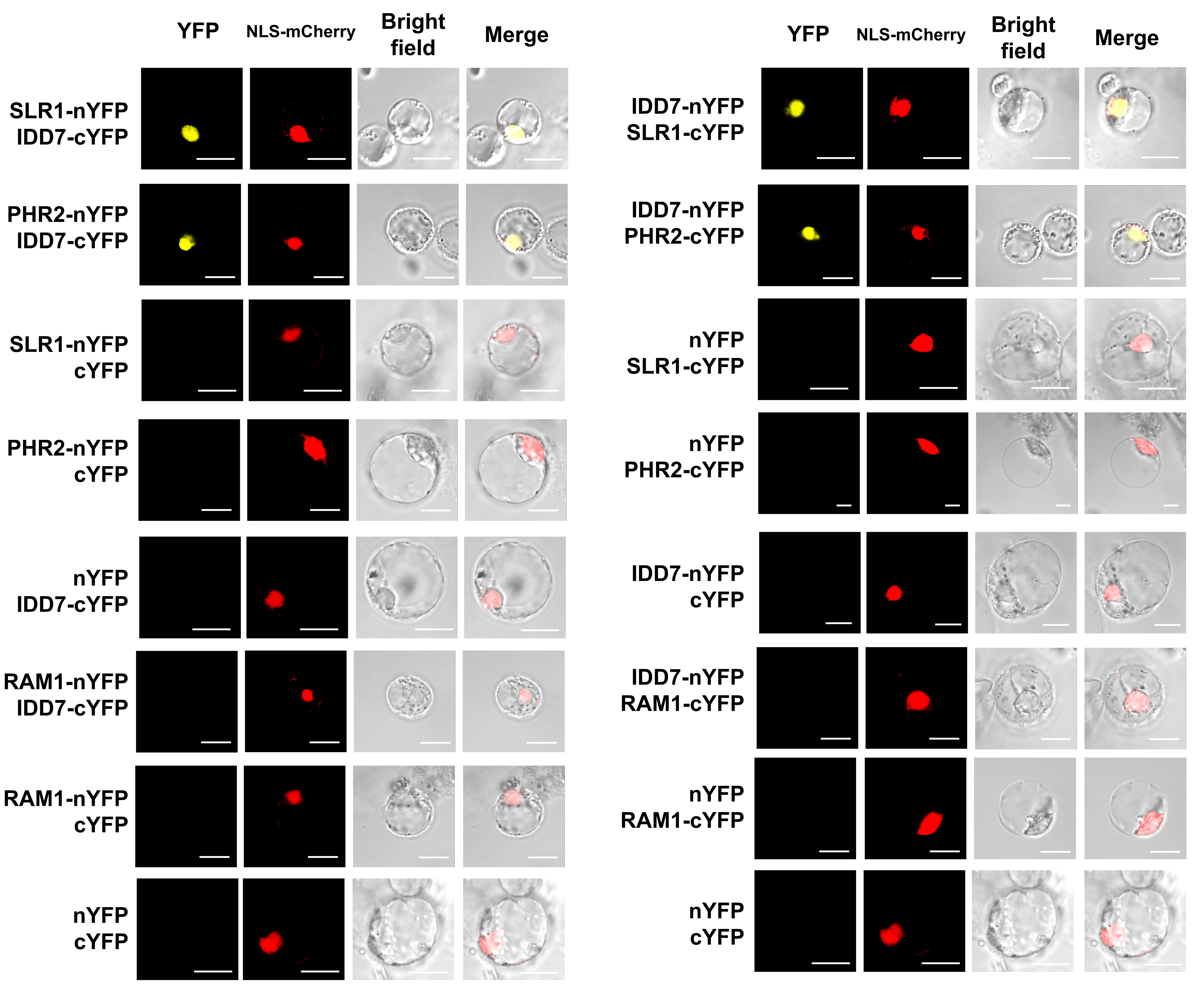

Supplementary Fig.6 (continued)

d

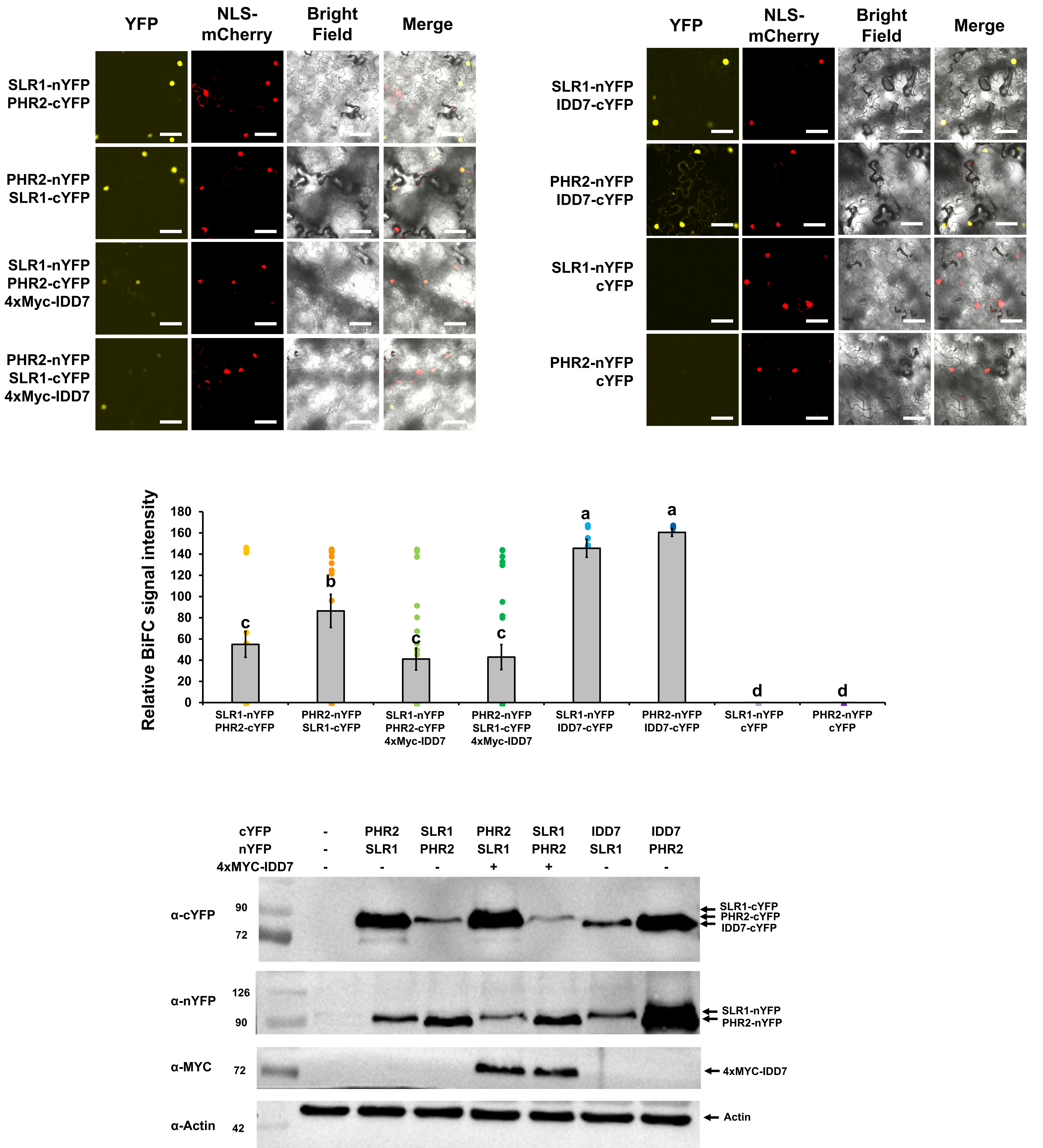

Supplementary Fig.6 (continued)

e

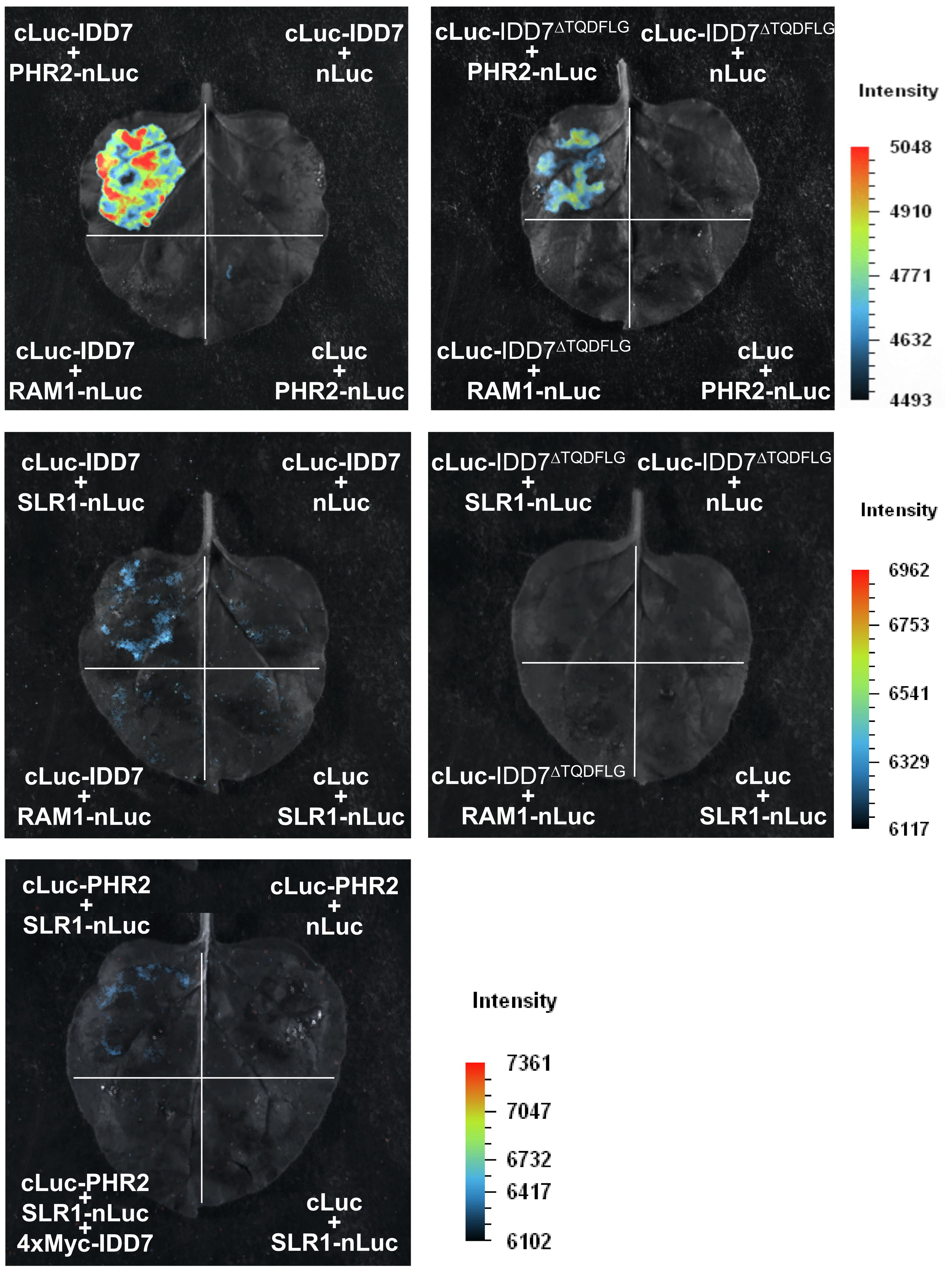

g

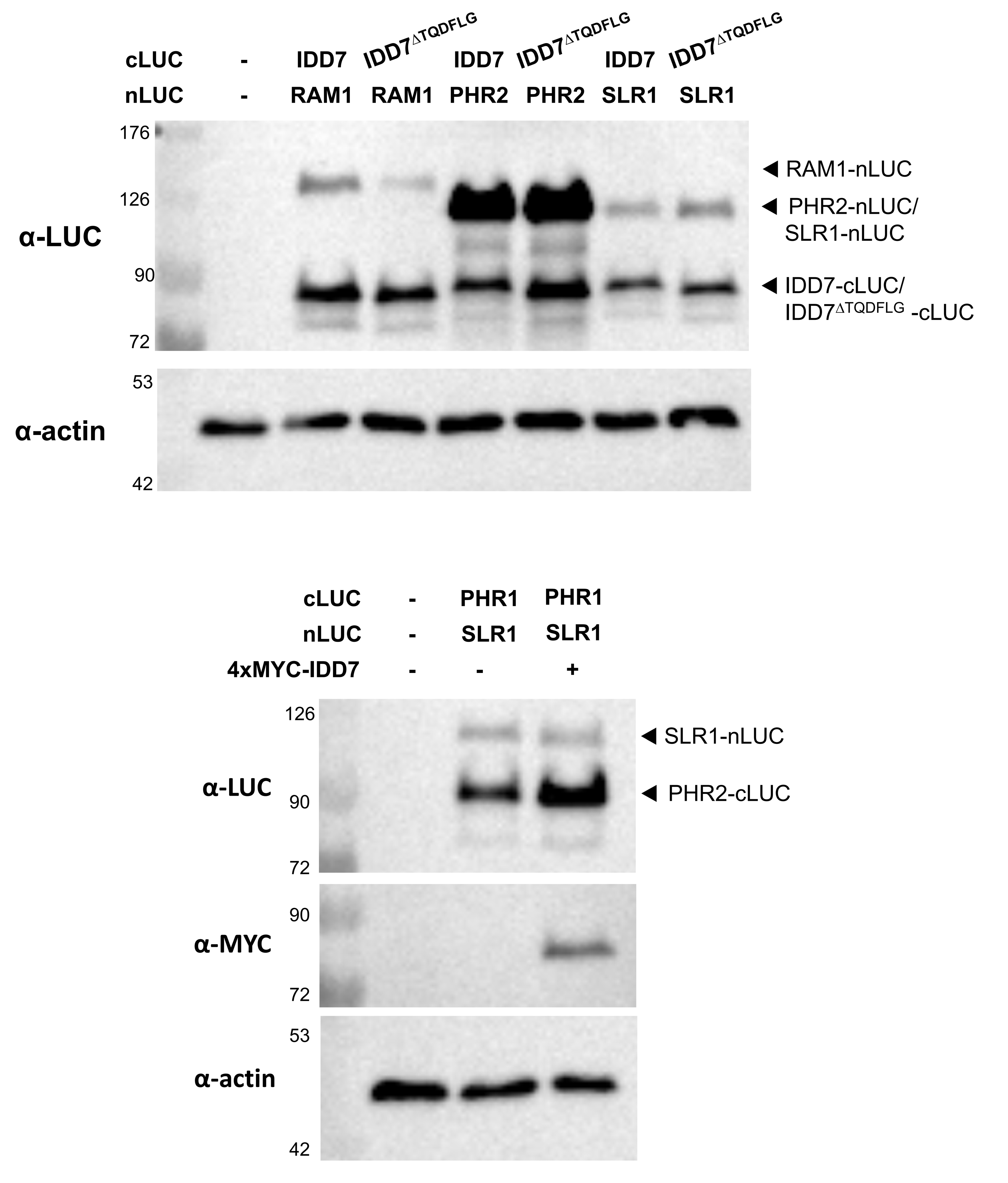

f

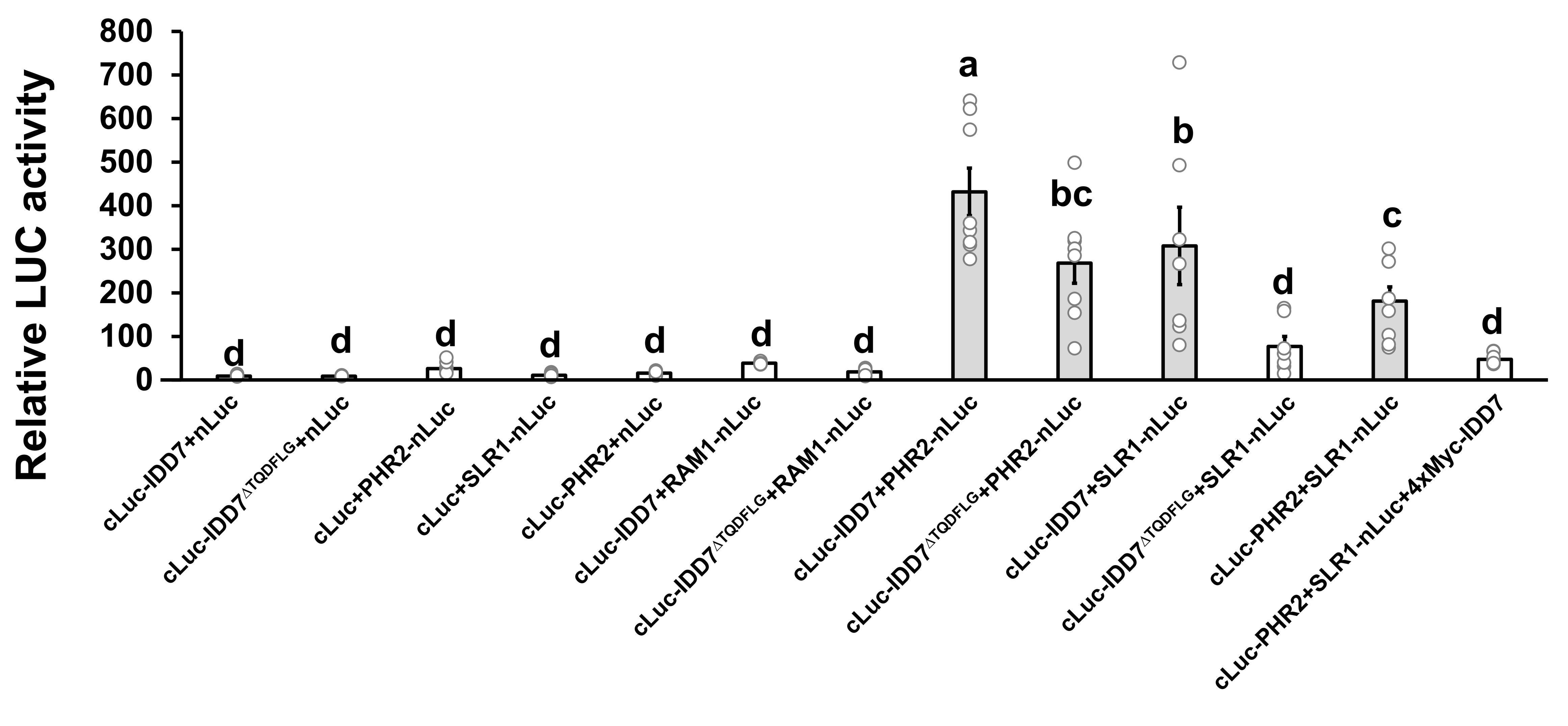

Supplementary Fig.6 (continued)

h

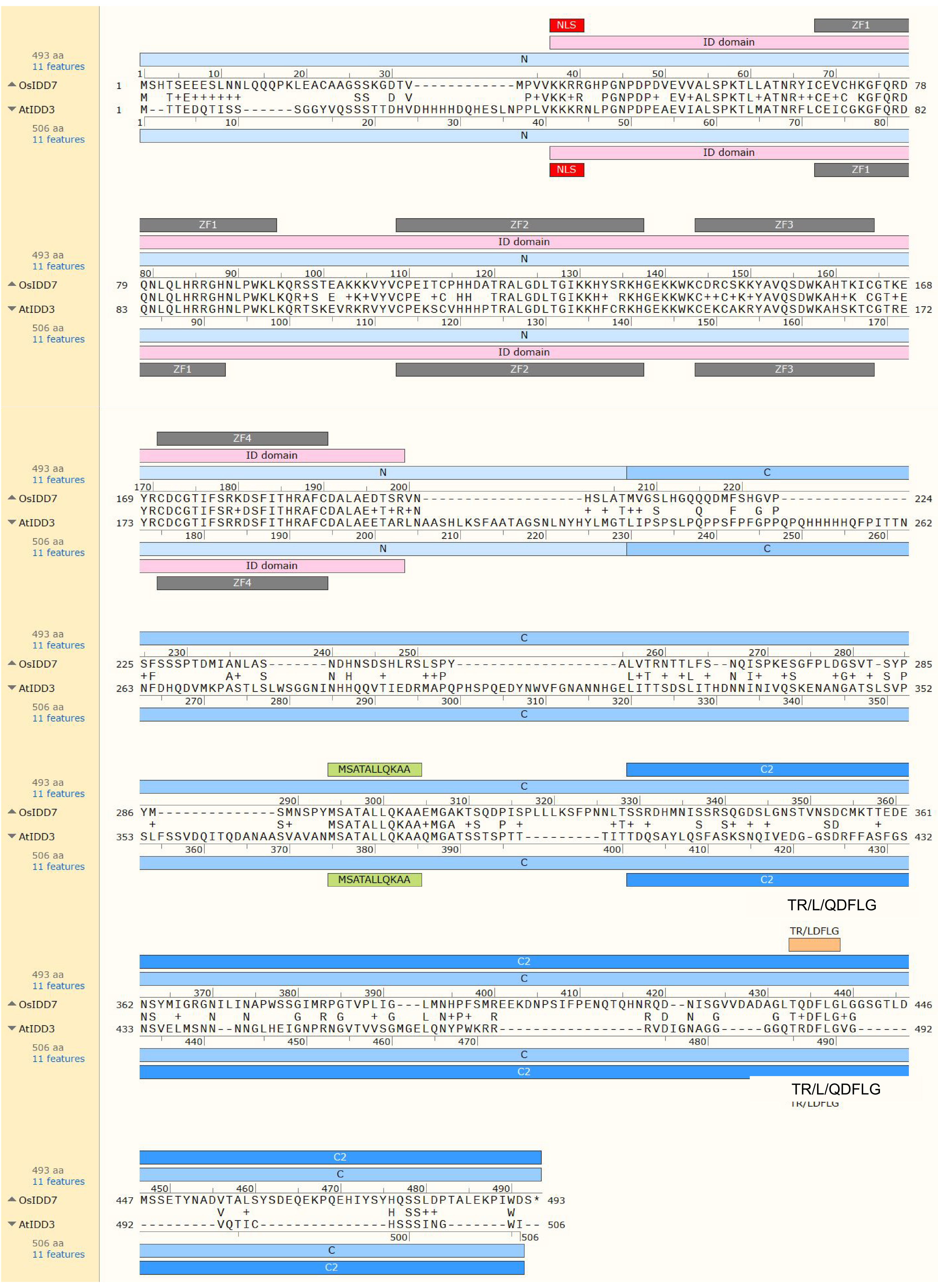

i

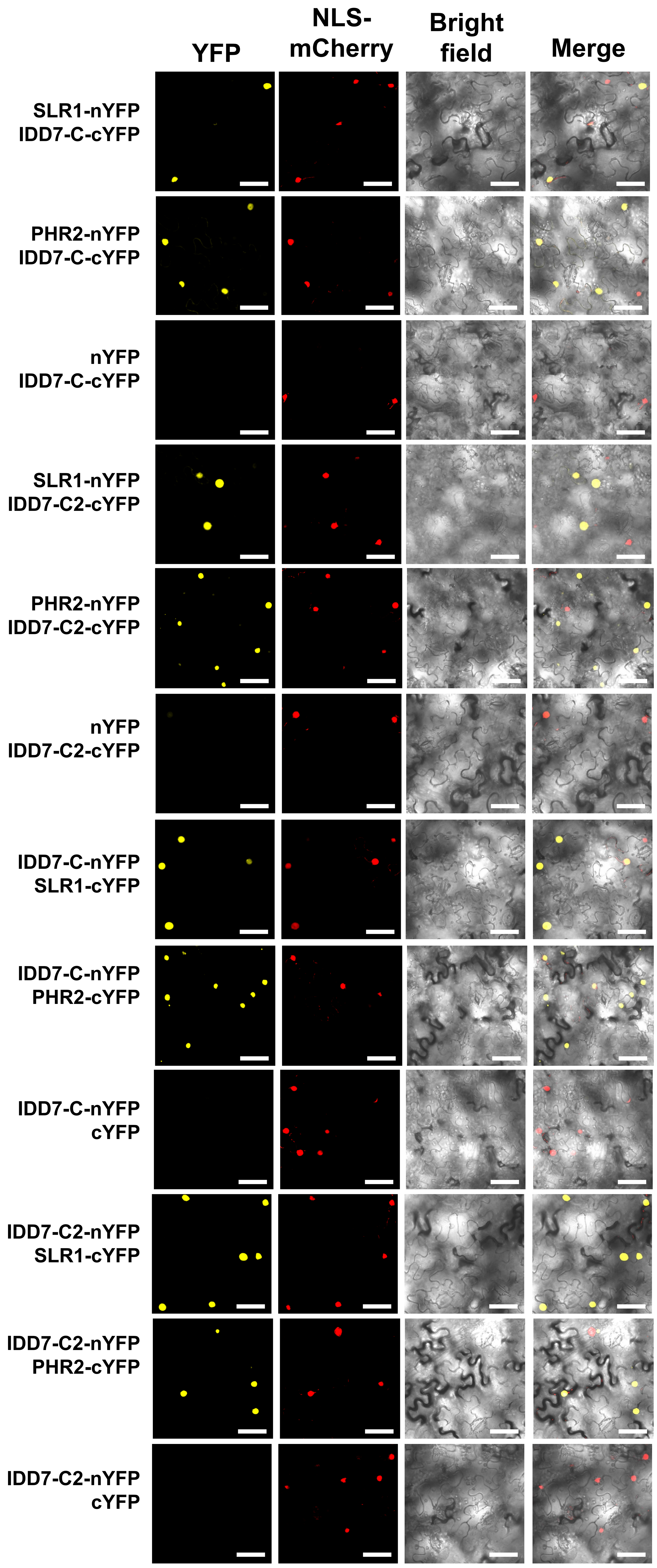

Supplementary Fig.6 (continued)

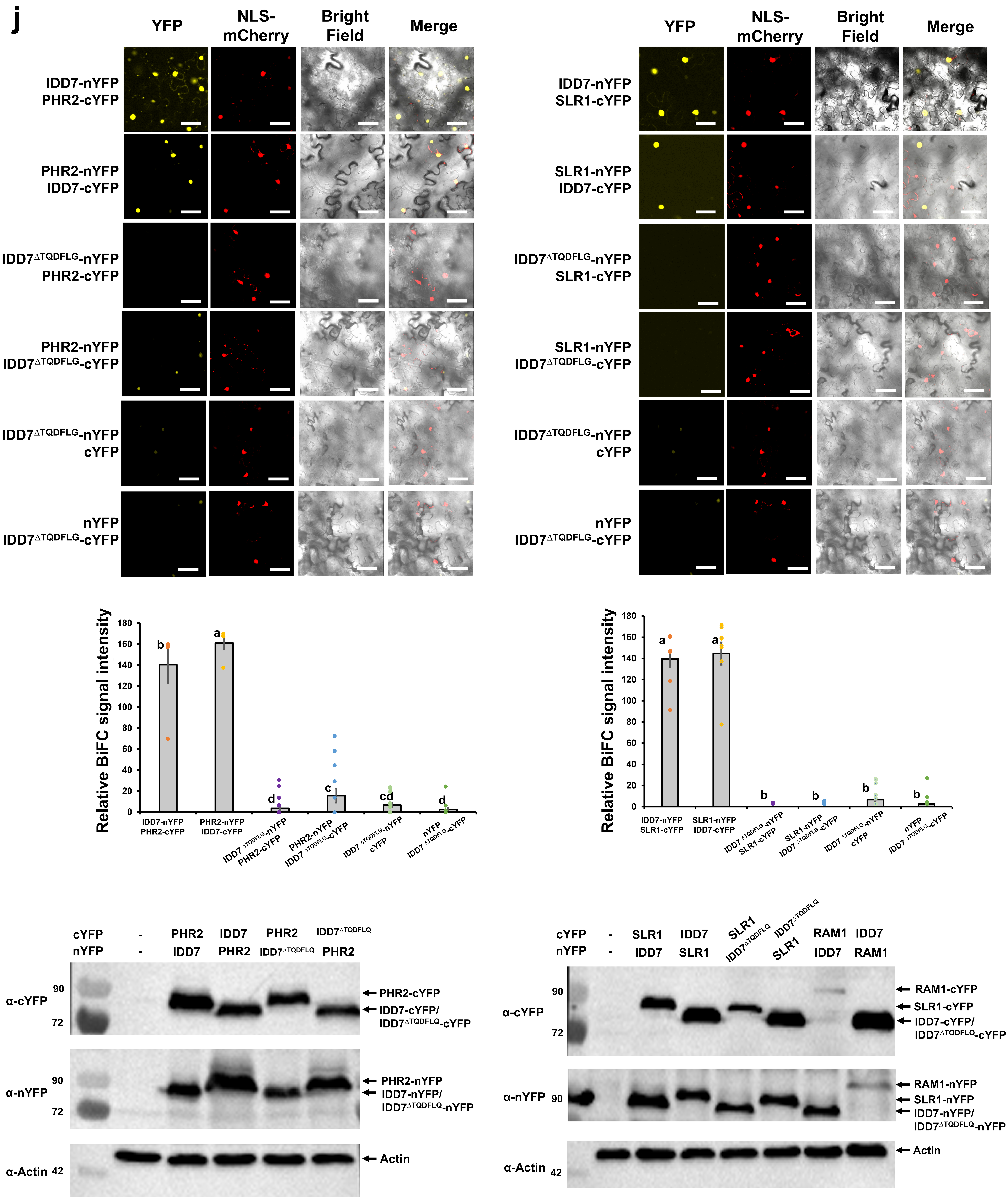

Supplementary Figure 6. IDD7 may interact with SLR1 and PHR2 through the TQDFLG domain *in planta*. a Analysis of the interaction between IDD7 or IDD7 lacking the TQDFLG domain (IDD7$^{\Delta TQDFLG}$) and SLR1, PHR2 or RAM1 using a BiFC assay in *N. benthamiana* leaves. **b** Co-immunoprecipitation (co-IP) assays were performed in *Nicotiana benthamiana* leaves transiently expressing the indicated constructs. Total protein extracts were immunoprecipitated with GFP-Trap beads, and co-immunoprecipitated proteins were detected using anti-His or anti-FLAG antibodies. Immunoprecipitated YFP fusion proteins were detected using anti-GFP antibody. Left, interaction between IDD7-YFP and PHR2-12×His. Right, interaction between IDD7-YFP and SLR1-3xFLAG. A short exposure of the input anti-GFP blot is shown to visualize YFP accumulation. Input samples are shown on the left. Data are representative of three independent biological experiments. **c** Analysis of the interaction between IDD7 and SLR1, PHR2 or RAM1 using a BiFC assay in rice protoplast. Scale bars = 10 μm. **d** Analysis of the interaction between PHR2 and SLR1 in the absence or presence of 4xMyc-IDD7 using a BiFC assay in *N. benthamiana* leaves. Relative YFP fluorescence signals (with brightness uniformly adjusted to 70% for visualization) are shown. YFP fluorescence intensity was quantified in nuclei exhibiting mCherry fluorescence under identical imaging settings. Relative BiFC signal intensity was quantified after background subtraction using ImageJ.JS (v0.6.0). Data are presented as mean ± SEM. Each replicate represents one independently quantified region. Sample sizes were n = 8 replicates for PHR2-nYFP/IDD7-cYFP and PHR2-nYFP/EV; n = 10 replicates for SLR1-nYFP/IDD7-cYFP and SLR1-nYFP/EV; n = 19 replicates for PHR2-nYFP/SLR1-cYFP; n = 23 replicates for SLR1-nYFP/PHR2-cYFP/IDD7; n = 24 replicates for PHR2-nYFP/SLR1-cYFP/IDD7; and n = 25 replicates for SLR1-nYFP/PHR2-cYFP. Immunoblot of the BiFC assay in which actin served as a loading control. **e** Interaction of IDD7 or IDD7$^{\Delta TQDFLG}$ with PHR2, IDD7 or IDD7$^{\Delta TQDFLG}$ with SLR1, and PHR2 with SLR1 in the presence or absence of IDD7 using a LCI assay in *N. benthamiana* leaves. The CDSs of *IDD7*, *IDD7$^{\Delta TQDFLG}$*, *PHR2*, *SLR1* and *RAM1* were fused with nLuc or cLuc. **f** Quantification of relative luciferase activity in leaves. Data are presented as mean ± SEM (n = 7 independently infiltrated leaf discs for cLuc-IDD7/SLR1-nLuc and cLuc-IDD7$^{\Delta TQDFLG}$/SLR1-nLuc, and n = 8 for all other groups). Each leaf disc represents an independently infiltrated leaf, with one or two leaves per plant used for a given treatment. **g** Immunoblot of the LCI assay in which actin served as a loading control. **h** Protein alignment between OsIDD7 and AtIDD3. Amino acid sequence alignment between OsIDD7 and AtIDD3. NLS: nuclear localization sequence (red box); ZF1 to 4: zinc finger 1, 2, 3, 4 (gray box); ID domain (pink box); MSATALLQKAA motif (green box); and TR/L/QDFLG domain (orange box). N: N-terminal domain; C: C-terminal domain; C2: C2-terminal domain (blue box). The full-length protein sequences were aligned using global alignment (the Needleman–Wunsch algorithm) in SnapGene. **i** The interaction between the C- or C2-terminus of IDD7 and SLR1 or PHR2 *in N. benthamiana* leaves was demonstrated using a BiFC assay. Full-length CDSs without stop codons of IDD7, C-terminus and C2-terminus without the stop codon of *IDD7*, *SLR1*, *PHR2*, and *RAM1* were fused with cYFP or nYFP. Combinations of nonfused nYFP or cYFP served as negative controls, while NLS-mCherry was utilized as a nuclear marker. Scale bars = 50 μm. **j** Representative BiFC images of IDD7$^{\Delta TQDFLG}$ in combination with PHR2 or SLR1 showing relative YFP fluorescence signals (brightness uniformly adjusted to 70% for visualization). Data are presented as mean ± SEM. Each replicate represents one independently quantified region. IDD7$^{\Delta TQDFLG}$ is abbreviated as mIDD7. Sample sizes were n = 5 replicates for IDD7-nYFP/PHR2-cYFP and PHR2-nYFP/IDD7-cYFP; n = 8 for SLR1-nYFP/IDD7-cYFP; n = 9 for IDD7-nYFP/SLR1-cYFP; n = 13 for EV-nYFP/mIDD7-cYFP; n = 14 for PHR2-nYFP/mIDD7-cYFP, mIDD7-nYFP/EV-cYFP, and mIDD7-nYFP/SLR1-cYFP; n = 17 for SLR1-nYFP/mIDD7-cYFP; and n = 23 for mIDD7-nYFP/PHR2-cYFP. Immunoblot of the BiFC assay in which actin served as a loading control. Different letters represent significant differences among the treatments ($P < 0.05$, one-way ANOVA followed by a least significant difference post hoc test); exact P values for the post hoc pairwise comparisons are provided in the Source Data. Representative BiFC, immunoblot, and co-IP results are shown; the experiments were independently repeated twice with similar results.

Supplementary Fig.7

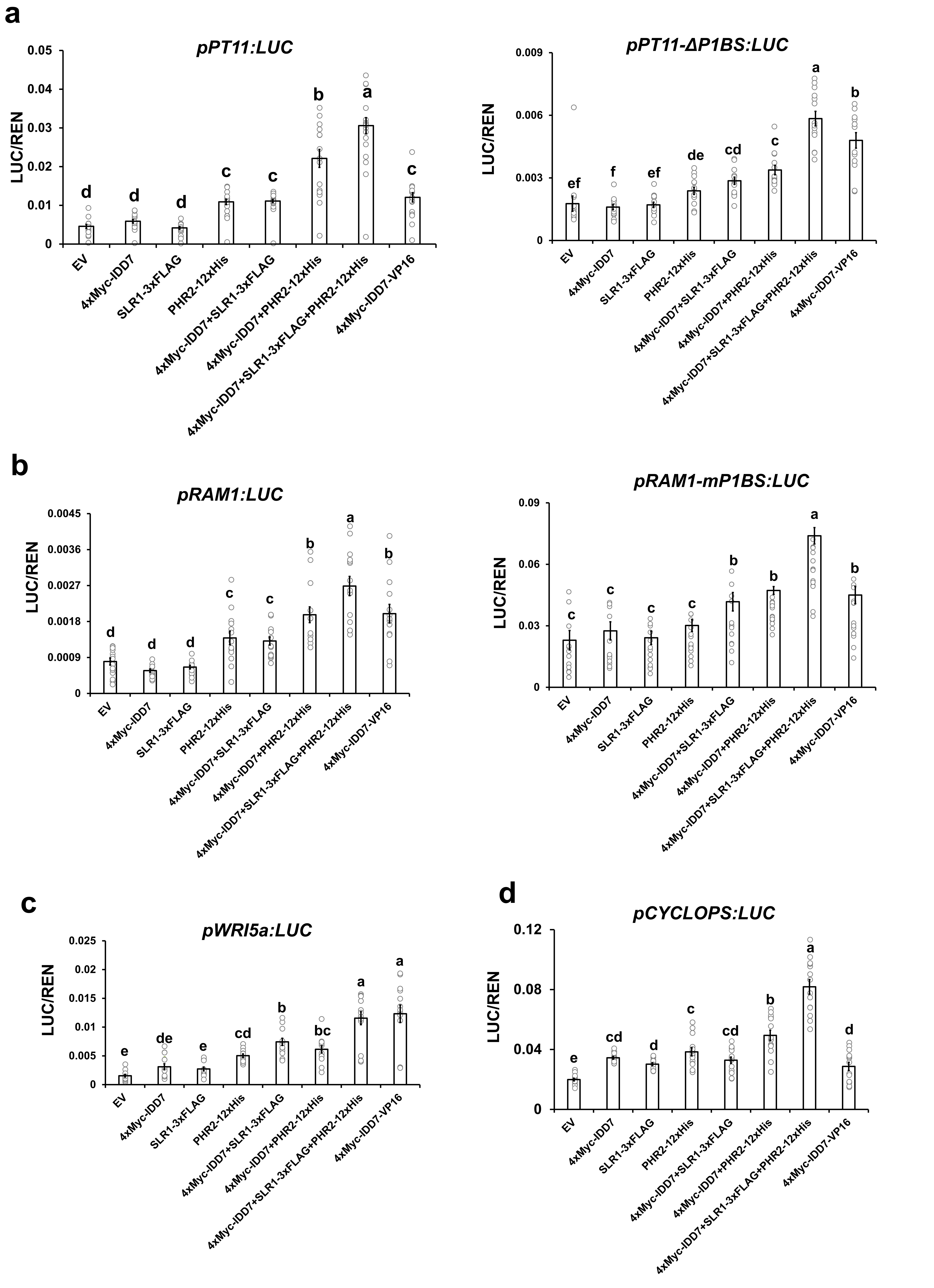

Supplementary Fig.7 (continued)

e

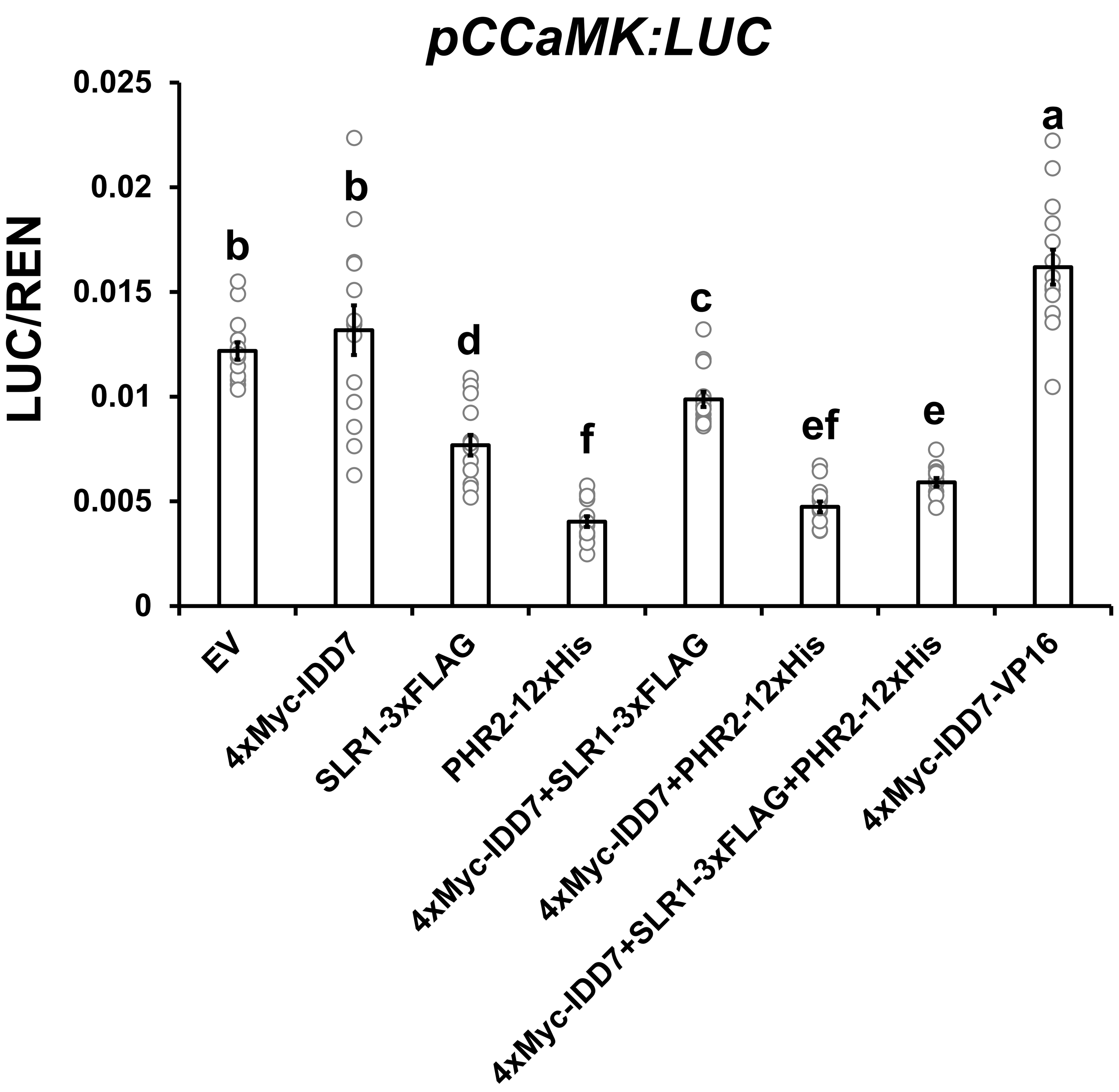

f

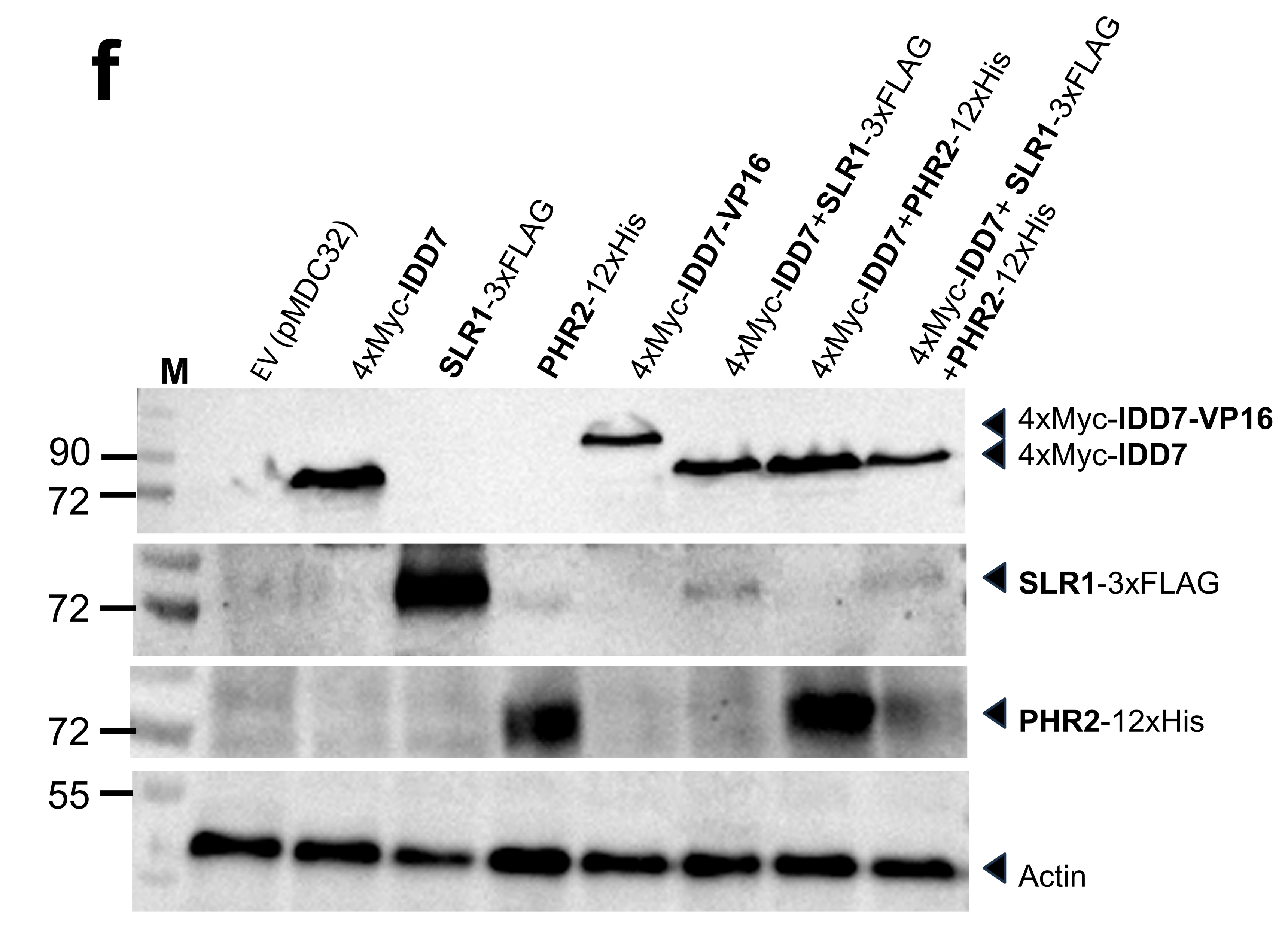

g

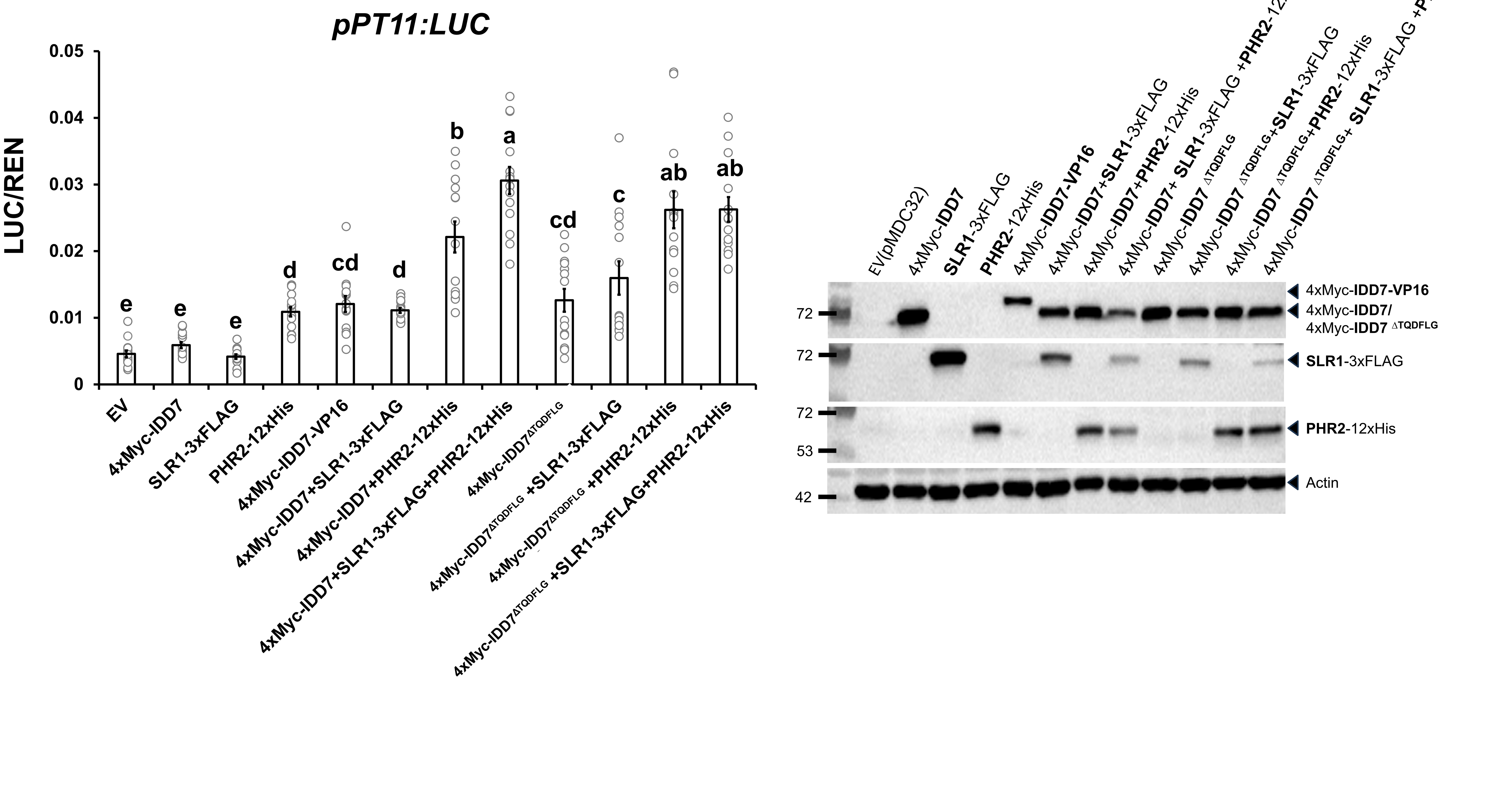

Supplementary Fig.7 (continued)

h

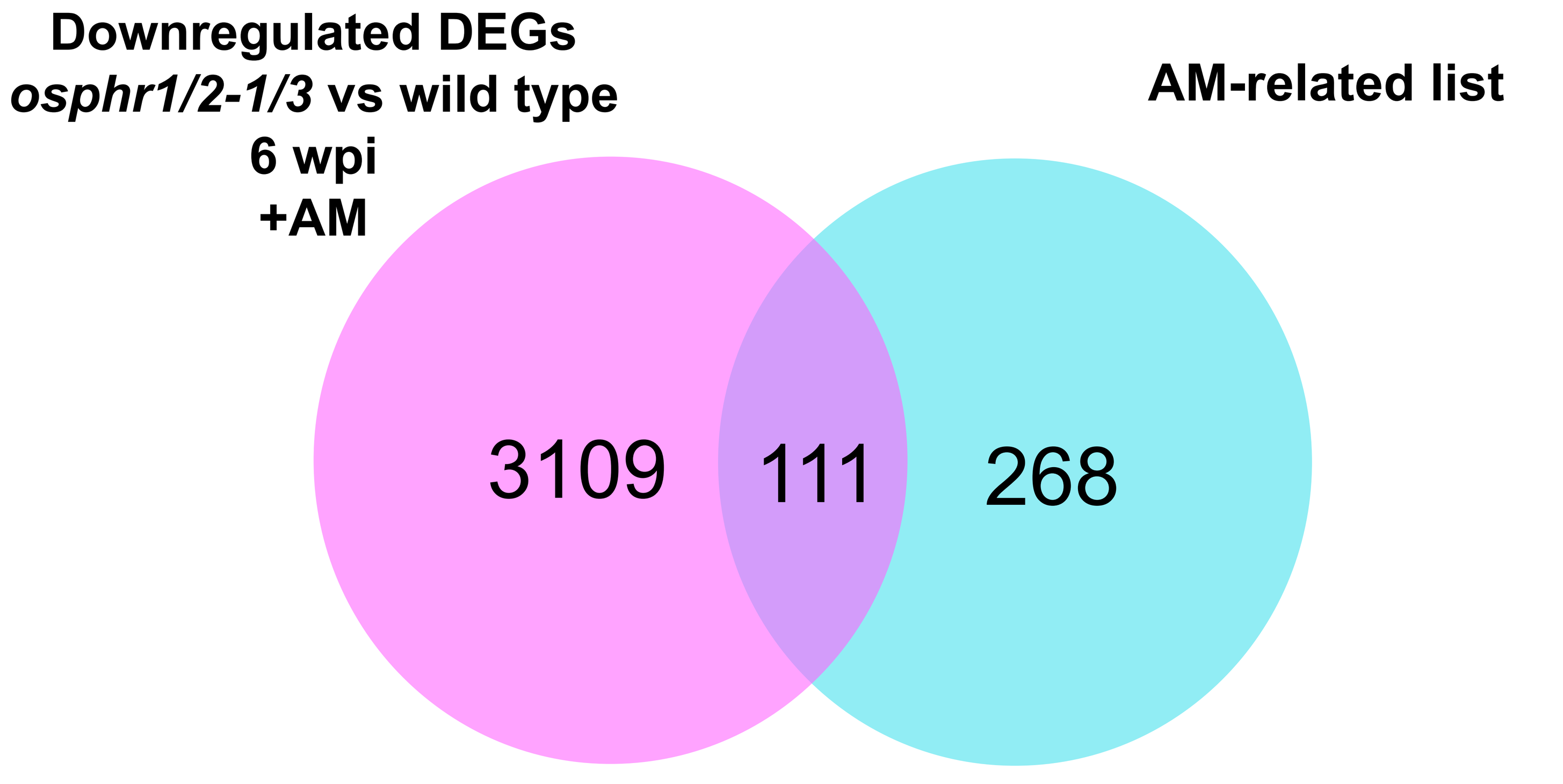

i

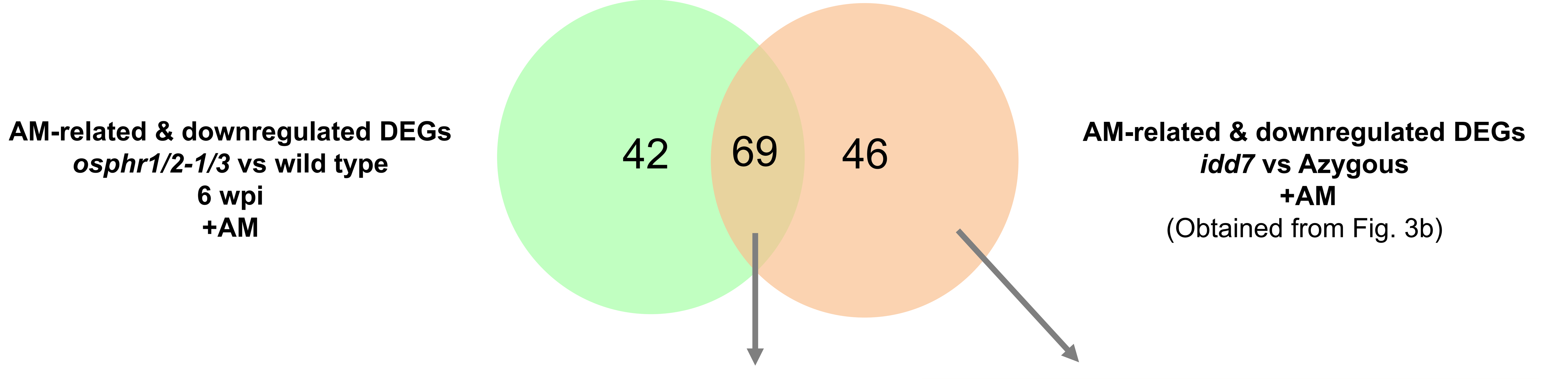

AM-related gene	Locus ID
PT11	LOC_Os01g46860
PT13	LOC_Os04g10800
STR	LOC_Os09g23640
HA1	LOC_Os03g01120
AMT2;3	LOC_Os01g65000
NPF4.5	LOC_Os01g54515
ABCB12	LOC_Os03g08380
NOPE1	LOC_Os04g01520
CYCLOPS	LOC_Os06g02520
FatM	LOC_Os01g31760
RAM2	LOC_Os03g52570
WRI5a	LOC_Os06g05340
NF-YC3	LOC_Os04g58680
NSP2	LOC_Os03g15680
CCD7	LOC_Os04g46470
CCD8	LOC_Os01g54270
CKL2	LOC_Os02g20140
LYK1	LOC_Os01g36550
ADK1	LOC_Os01g65940
RFCb	LOC_Os10g42400

AM-related gene	Locus ID
ARK2	LOC_Os04g39180
EXO70I	LOC_Os08g40840
KIN2	LOC_Os06g44430
VAPYRIN	LOC_Os03g45290
WRI5b	LOC_Os05g45954
MYR1	LOC_Os03g13080
DIP1	LOC_Os12g06540
SMAX1	LOC_Os08g15230

Supplementary Figure 7. IDD7 collaborates with SLR1 and PHR2 to achieve the strongest activation of the *RAM1*, *WRI5a,* and *CYCLOPS* promoters, but not CCaMK. Transactivation assay (DLR assay) of **a** *pPT11:LUC* and *pPT11-ΔP1BS:LUC*, **b** *pRAM1:LUC* and *pRAM1-mP1BS:LUC*, **c** *pWRI5a:LUC*, **d** *pCYCLOPS:LUC*, **e** *pCCaMK:LUC*, and **g** *pPT11:LUC* in *N. benthamiana* leaves. Reporter constructs containing the indicated promoter fragments (*pPT11*, 3700 bp; *pPT11-ΔP1BS*, 3660 bp; *pRAM1* and *pRAM1-mP1BS*, 3000 bp; *pWRI5a*, 2500 bp; *pCYCLOPS*, 2500 bp; and *pCCaMK*, 2000 bp) were fused to firefly luciferase (LUC) and co-expressed with p35S:REN (Renilla luciferase) together with the indicated effector constructs, including EV (empty vector), 4×Myc-IDD7, 4×Myc-IDD7$^{\Delta TQDFLG}$, SLR1-3×FLAG, PHR2-12×His, and 4×Myc-IDD7-VP16. **f** Immunoblot analysis corresponding to the DLR assay shown in Fig. 5b, confirming accumulation of the indicated effector proteins. For the *PT11* promoter, the WT data shown in Fig. 5b are reproduced in Supplementary Fig. 7a solely to facilitate comparison with the ΔP1BS construct. **g** Immunoblot analysis corresponding to the DLR assay shown in (g), confirming accumulation of 4×Myc-IDD7, 4×Myc-IDD7ΔTQDFLG, SLR1-3×FLAG, and PHR2-12×His. Actin served as a loading control. Data are presented as mean ± SEM (n = 14 leaf discs from 14 independently infiltrated leaves per treatment, with one or two leaves per plant used for a given treatment). Different letters represent significant differences among the treatments ($P < 0.05$, one-way ANOVA followed by a least significant difference post hoc test); exact P values for the post hoc pairwise comparisons are provided in the Source Data. Representative immunoblot results are shown; the experiments were independently repeated twice with similar results. **h** Venn diagram showing the overlapping genes of the AM-related list used in Fig. 3b and the downregulated DEGs of '*osphr1/2-1/3* vs. wild type' in AM-inoculated roots collected at 6 weeks postinoculation (wpi) in Shi et al. (2022)[21]. **i** Venn diagram showing the intersection of AM-related DEGs between '*osphr1/2-1/3* vs. wild type' and '*idd7* vs. Azygous' in AM-inoculated roots. The wild type used in Shi et al. (2022) is Nipponbare. The overlapping gene list is further shortlisted to some genes with previously established functions in AM symbiosis in rice or other plant species.

Supplementary Fig.8

a

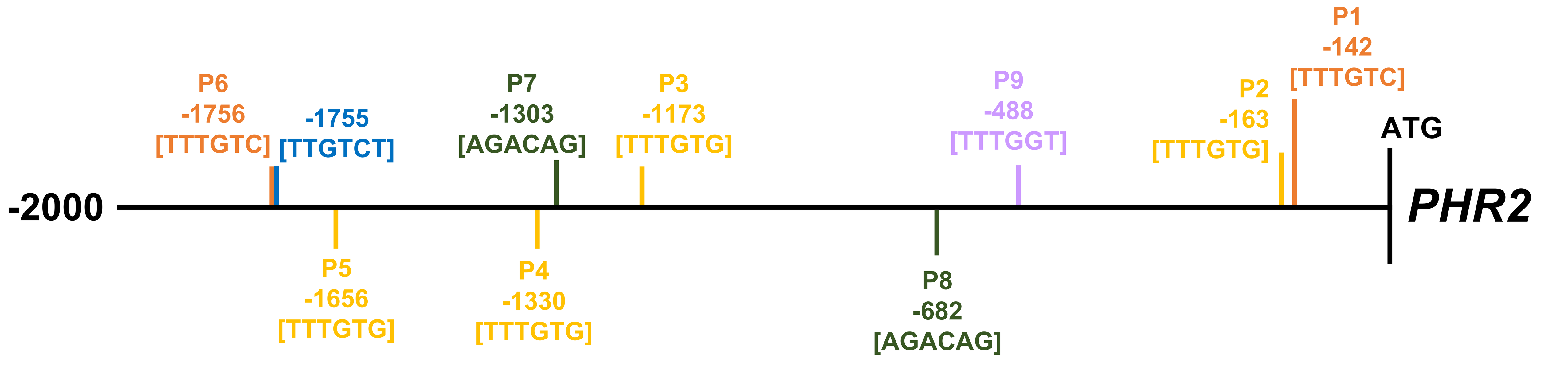

Supplementary Fig.8 (continued)

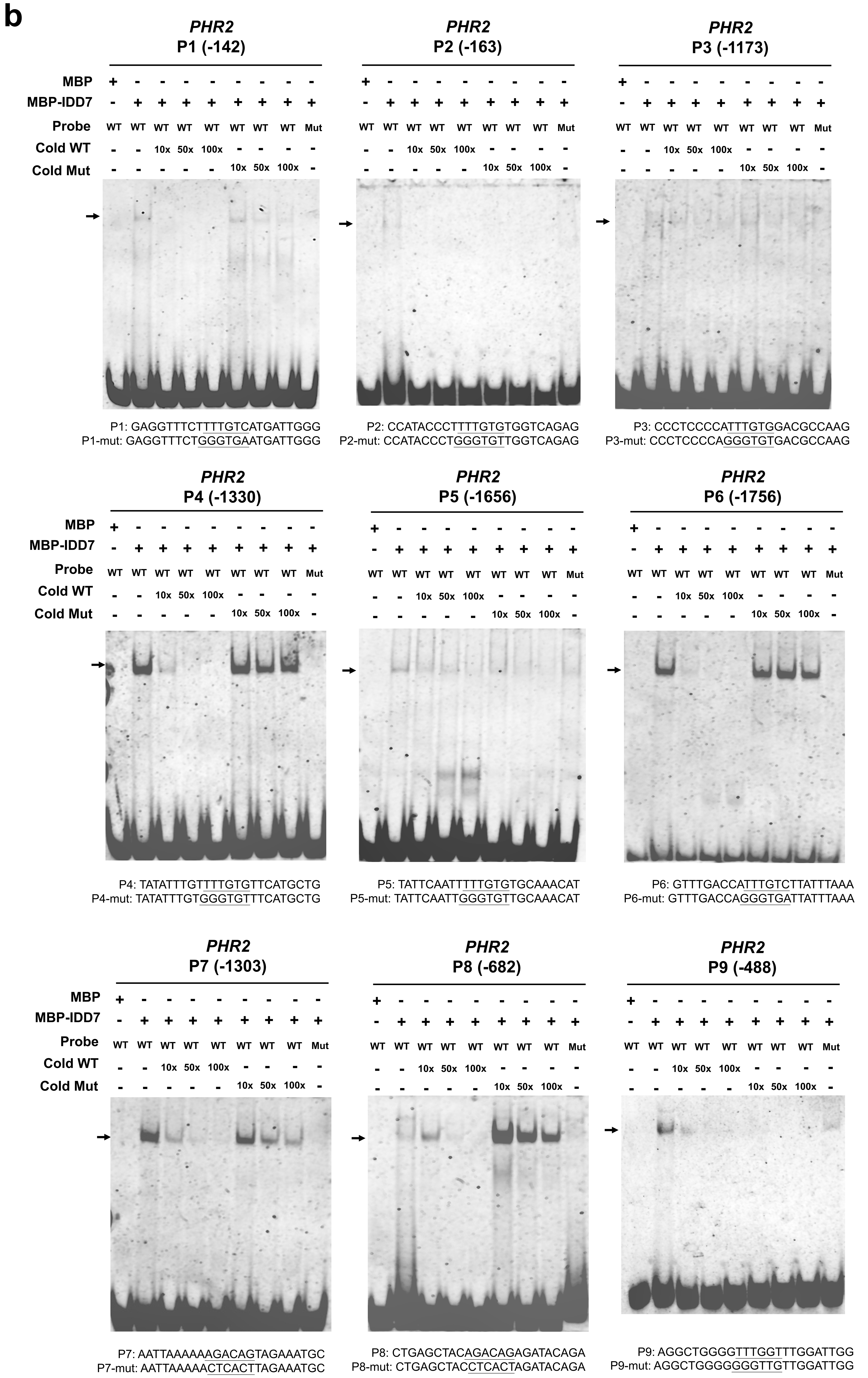

Supplementary Fig.8 (continued)

c

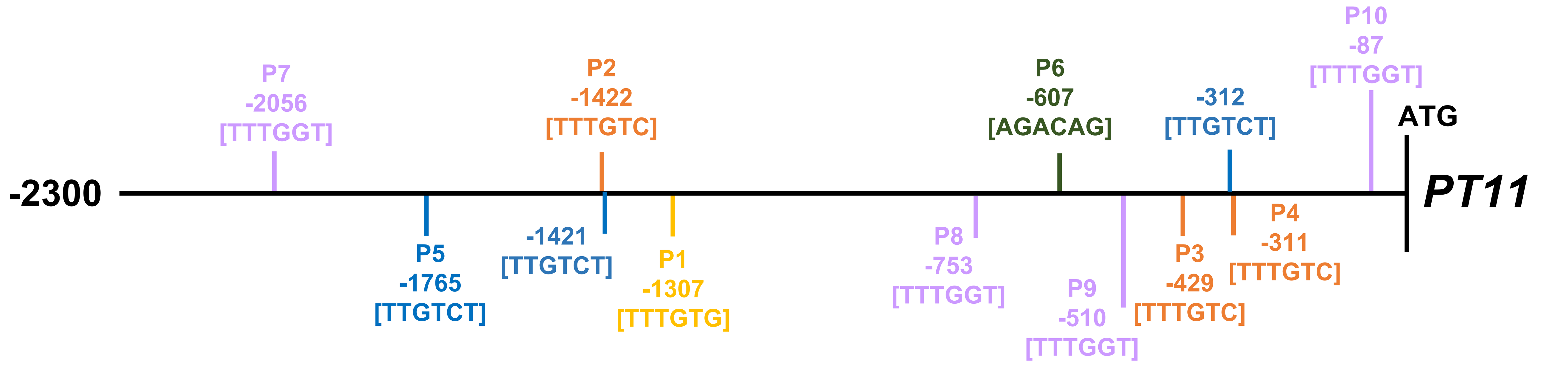

Supplementary Fig.8 (continued)

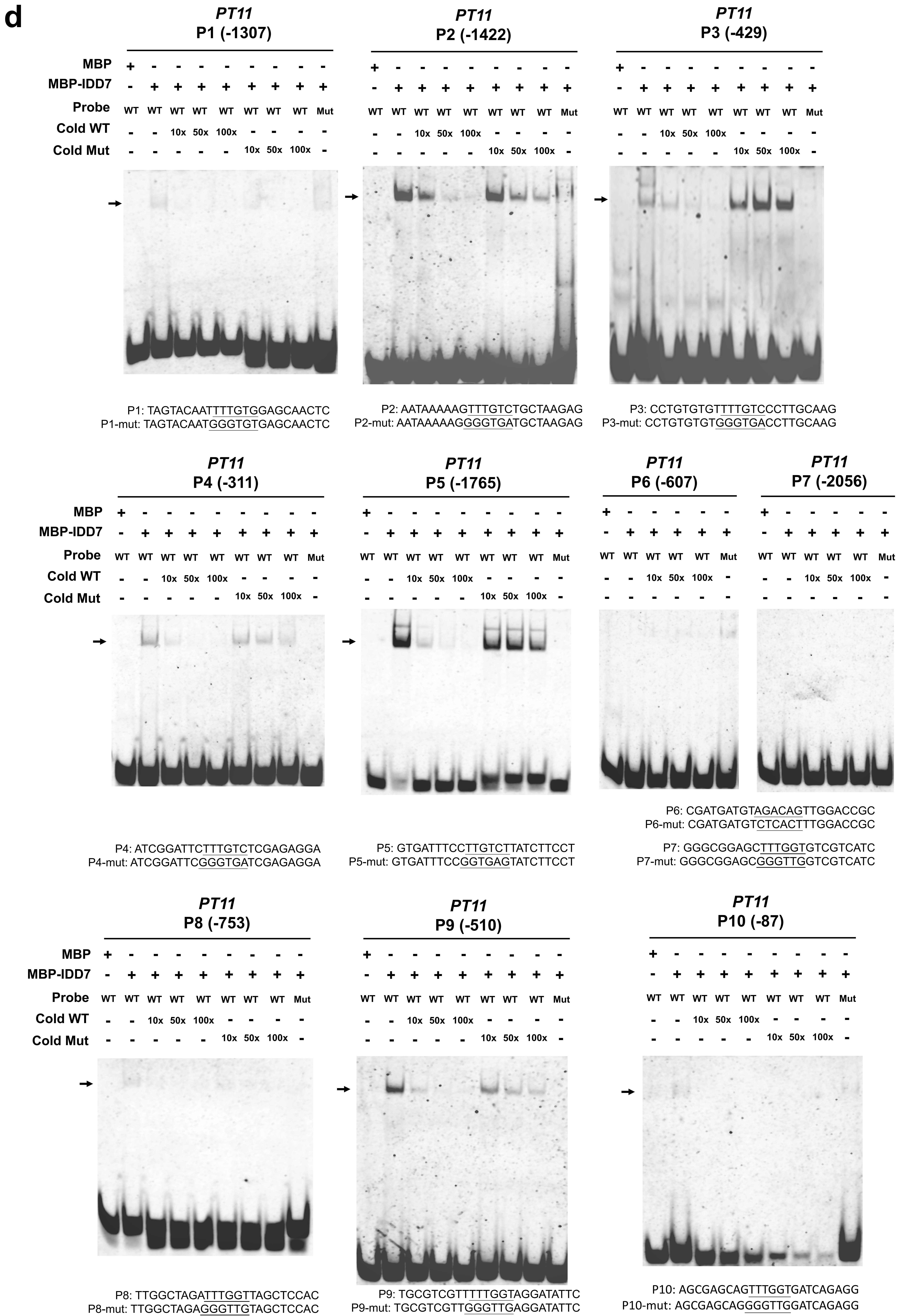

Supplementary Fig.8 (continued)

e

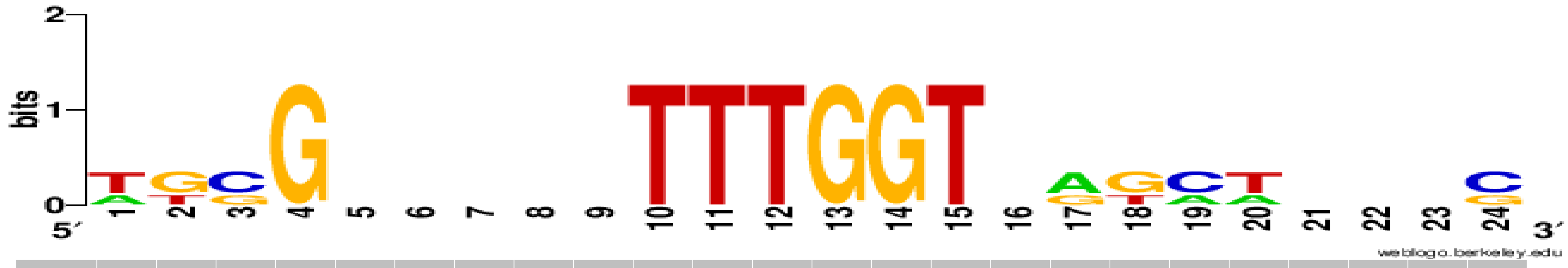

		1	2	3	4	5	6	7	8	9	10	11	12	13	14	15	16	17	18	19	20	21	22	23	24
	P7	G	G	G	C	G	G	A	G	C	T	T	T	G	G	T	G	T	C	G	T	C	A	T	C
pPT11	P8	T	T	G	G	C	T	A	G	A	T	T	T	G	G	T	T	A	G	C	T	C	C	A	C
	P9	T	G	C	G	T	C	G	T	T	T	T	T	G	G	T	A	G	G	A	T	A	T	T	C
	P10	A	G	C	G	A	G	C	A	G	T	T	T	G	G	T	G	A	T	C	A	G	A	G	G

f

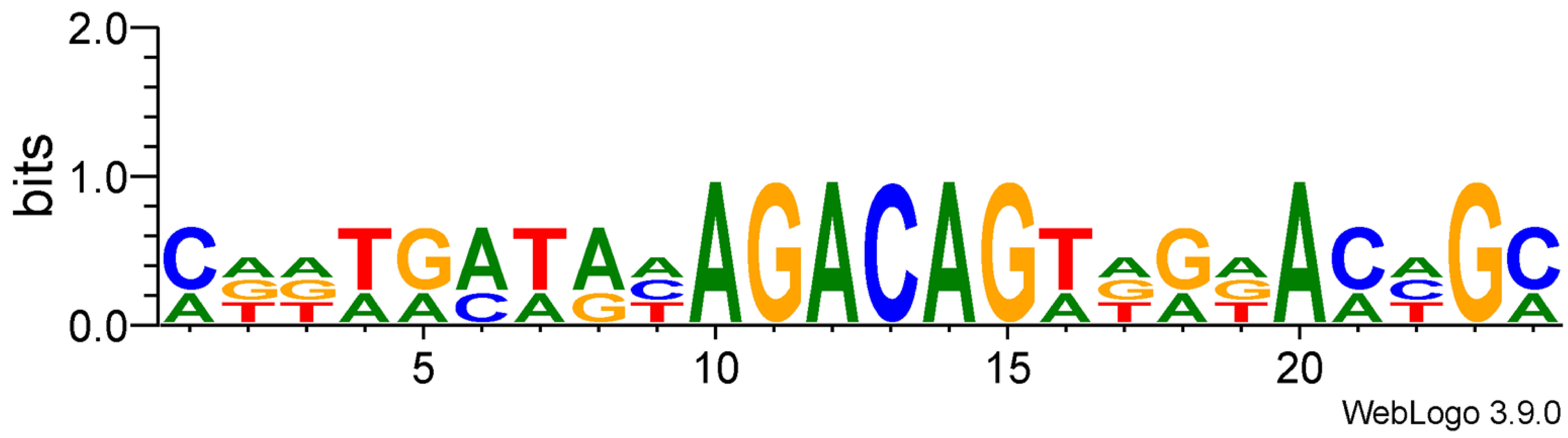

	1	2	3	4	5	6	7	8	9	10	11	12	13	14	15	16	17	18	19	20	21	22	23	24
**pPHR2_P7**	A	A	T	T	A	A	A	A	A	A	G	A	C	A	G	T	A	G	A	A	A	T	G	C
**pPHR2_P8**	C	T	G	A	G	C	T	A	C	A	G	A	C	A	G	A	G	A	T	A	C	A	G	A
**pPT11_P6**	C	G	A	T	G	A	T	G	T	A	G	A	C	A	G	T	T	G	G	A	C	C	G	C

Supplementary Figure 8. IDD7 can bind to motifs containing TTTGTG/C, AGACAG, or TTTGGT sequences on the *PHR2* promoter, and to motifs containing TTTGTG/C, TTGTCT, or TTTGGT sequences on the *PT11* promoter. a Schematic representations showing the locations of reported IDD binding sites (TTTGTG/C, AGACAG, TTTGGT and TTGTCT) on the 2000-bp *PHR2* promoter. EMSA probes contained the corresponding motifs along with flanking sequences, resulting in a total length of 24 bps. **b** Results of electrophoretic mobility shift assays (EMSAs). **c** Schematic representations showing the locations of reported IDD binding sites (TTTGTG/C, AGACAG, TTTGGT and TTGTCT) on the 2300-bp *PT11* promoter. EMSA probes contained the corresponding motifs along with flanking sequences, resulting in a total length of 24 bps. **d** Results of electrophoretic mobility shift assays (EMSAs). MBP and MBP-IDD7 were expressed in *E. coli BL21*. Wild-type (WT) and mutated (Mut) probes were synthesized and labeled with FAM at the 5’ end, whereas unlabeled WT probes (cold WT) and mutated probes (cold Mut) were used as competitors. Underlined sequences indicate the target motif and mutated sites. The black arrows indicate the expected shift locations. Representative EMSA results are shown; the experiments were independently repeated twice with similar results. **e** Sequence features of TTTGGT-containing probes associated with IDD7 binding. The sequence logo at the top was generated from probes containing the TTTGGT core motif (P8, P9, and P10). The alignment below shows sequence variation among probes P7–P10. Notably, the fourth position differs between bound and unbound probes: P7 contains a “T”, whereas P8, P9, and P10 contain a “G”, suggesting that this position may contribute to IDD7 binding specificity. **f** Sequence features of AGACAG-containing probes associated with IDD7 binding. The sequence logo at the top was generated from probes containing the AGACAG core motif (*PHR2*, P7/P8 and *PT11*, P6). The alignment below highlights sequence variation among these probes. In addition to the conserved AGACAG core, an upstream “A” is enriched among bound probes, suggesting that flanking nucleotides may contribute to IDD7 binding specificity.

Supplementary Fig.9

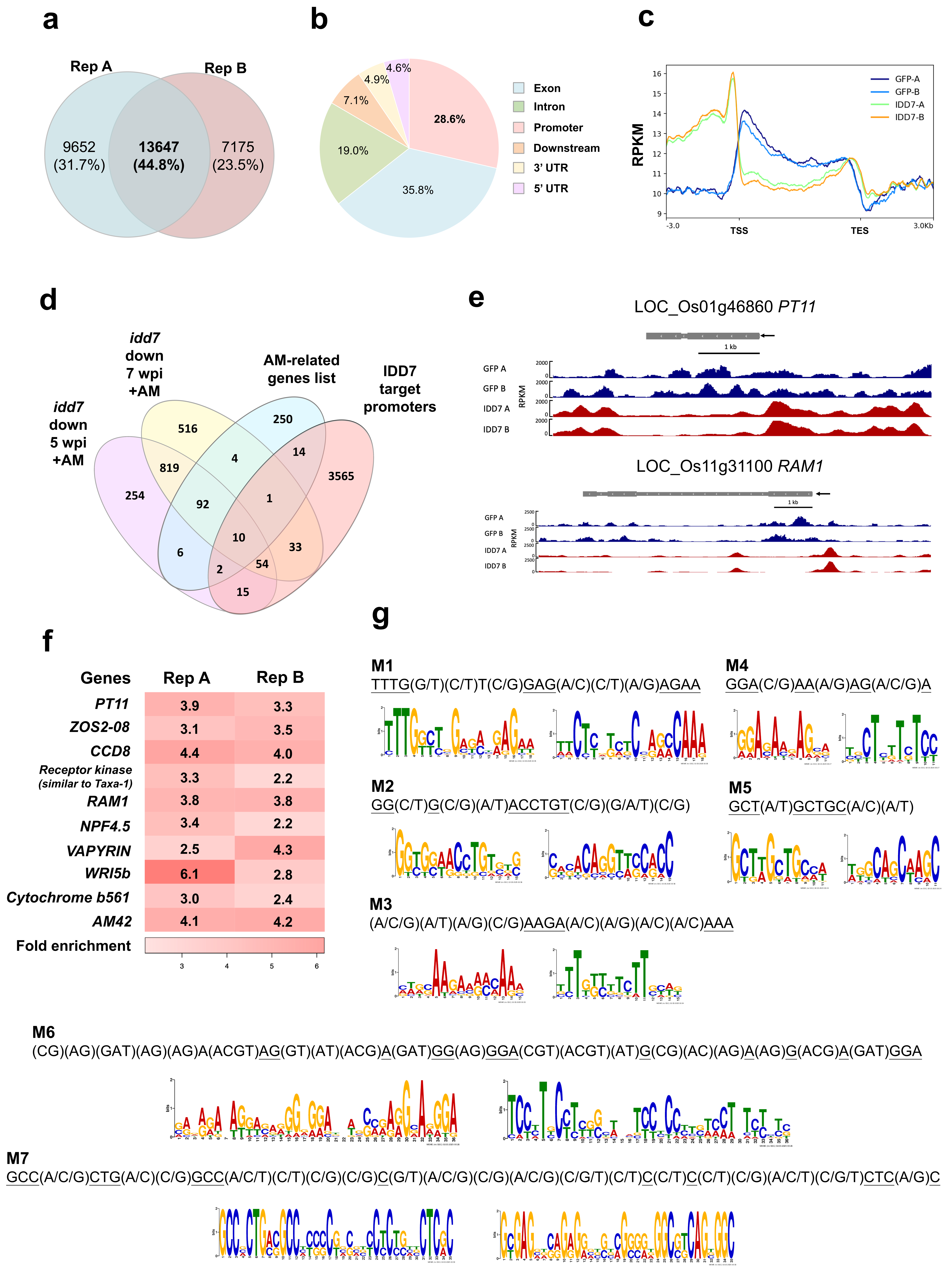

Supplementary Fig.9 (continued)

h

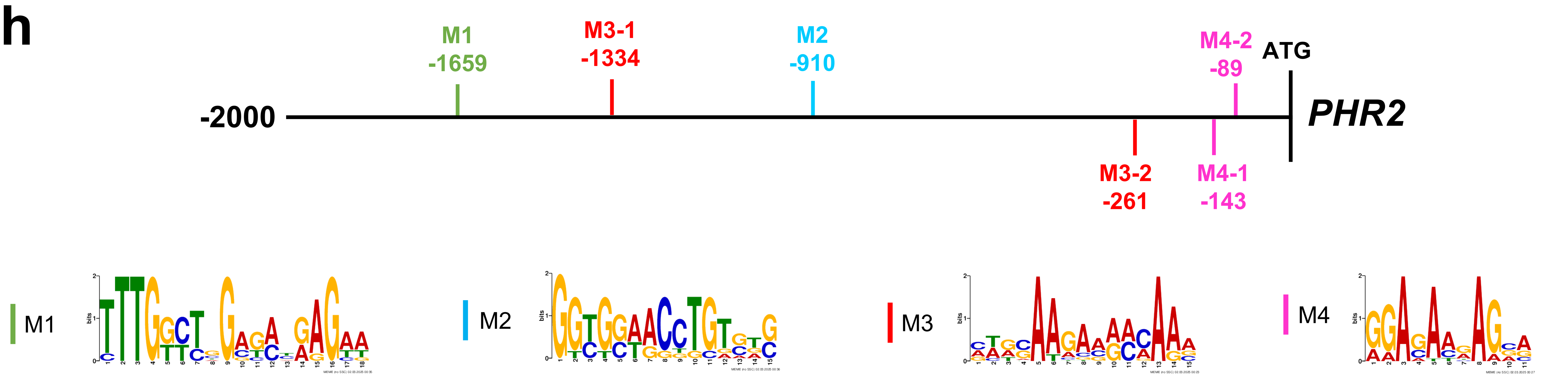

i

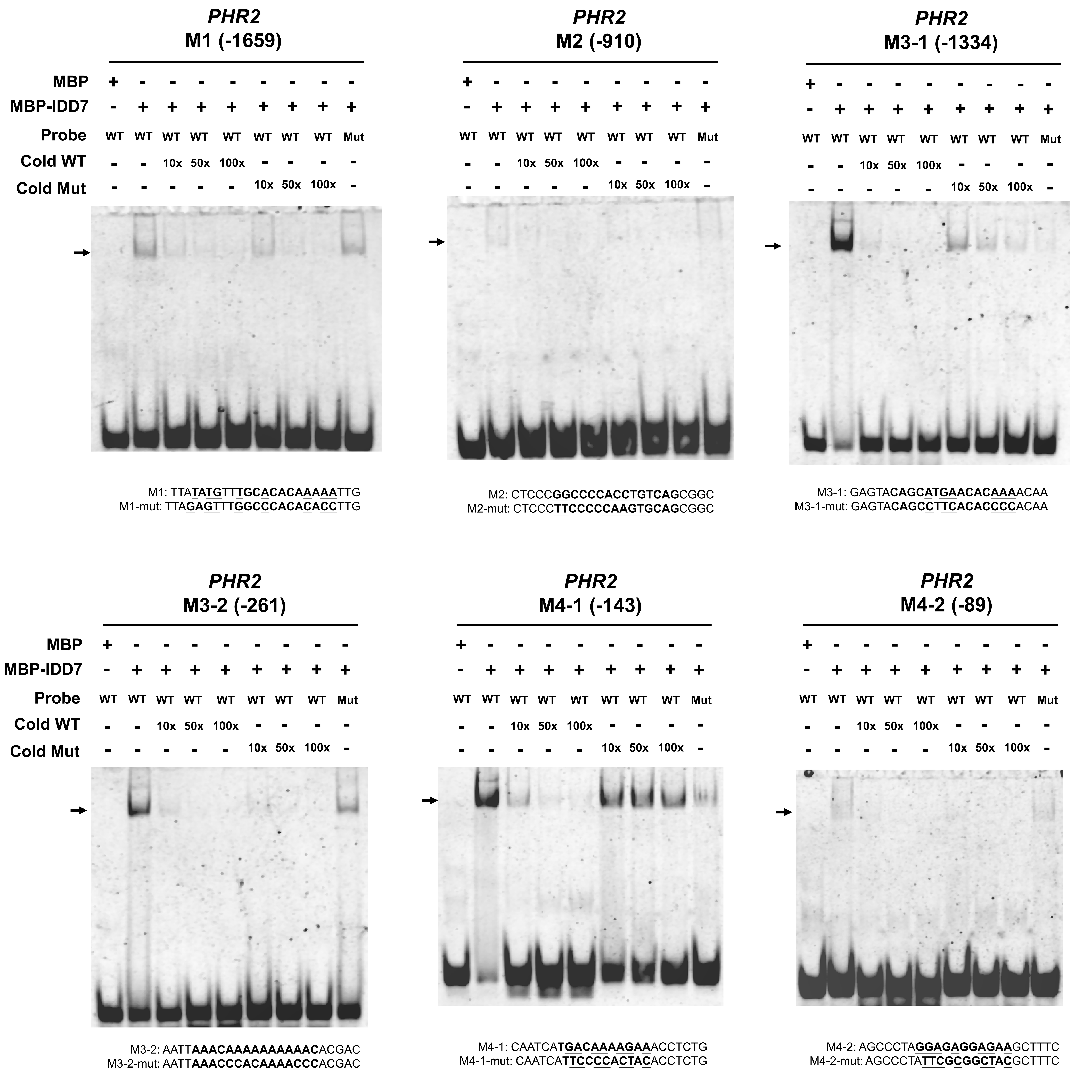

Supplementary Fig.9 (continued)

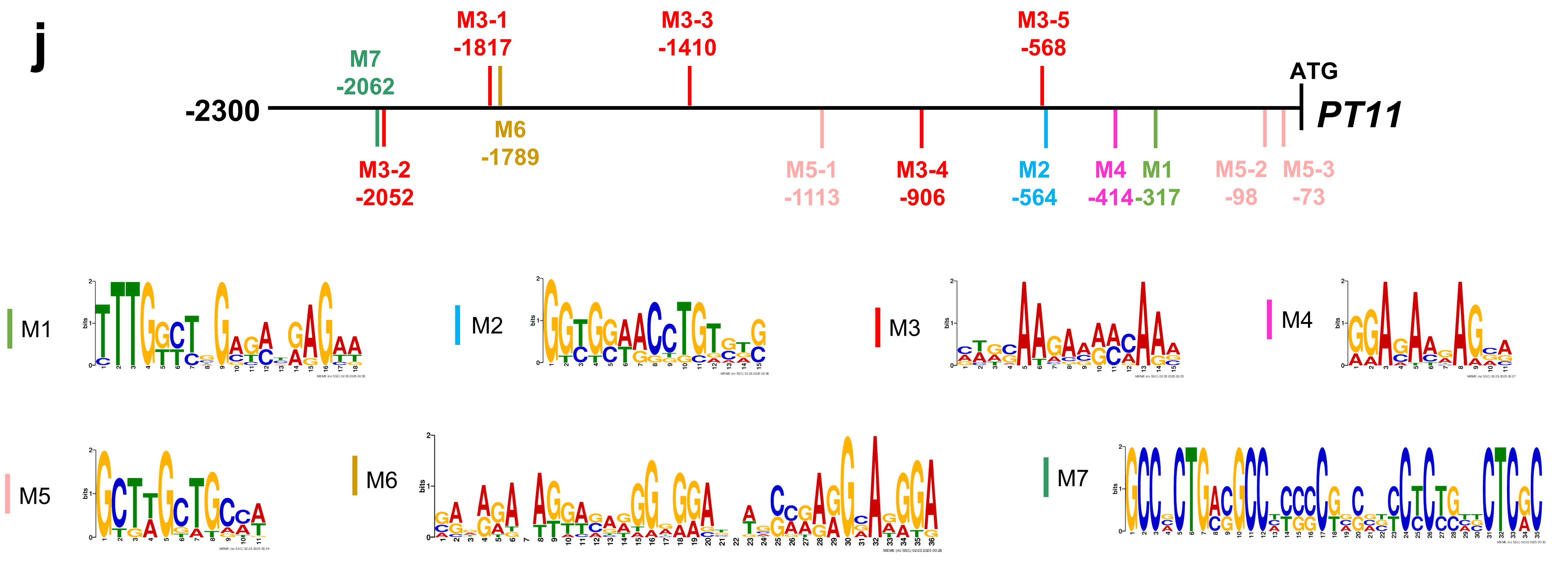

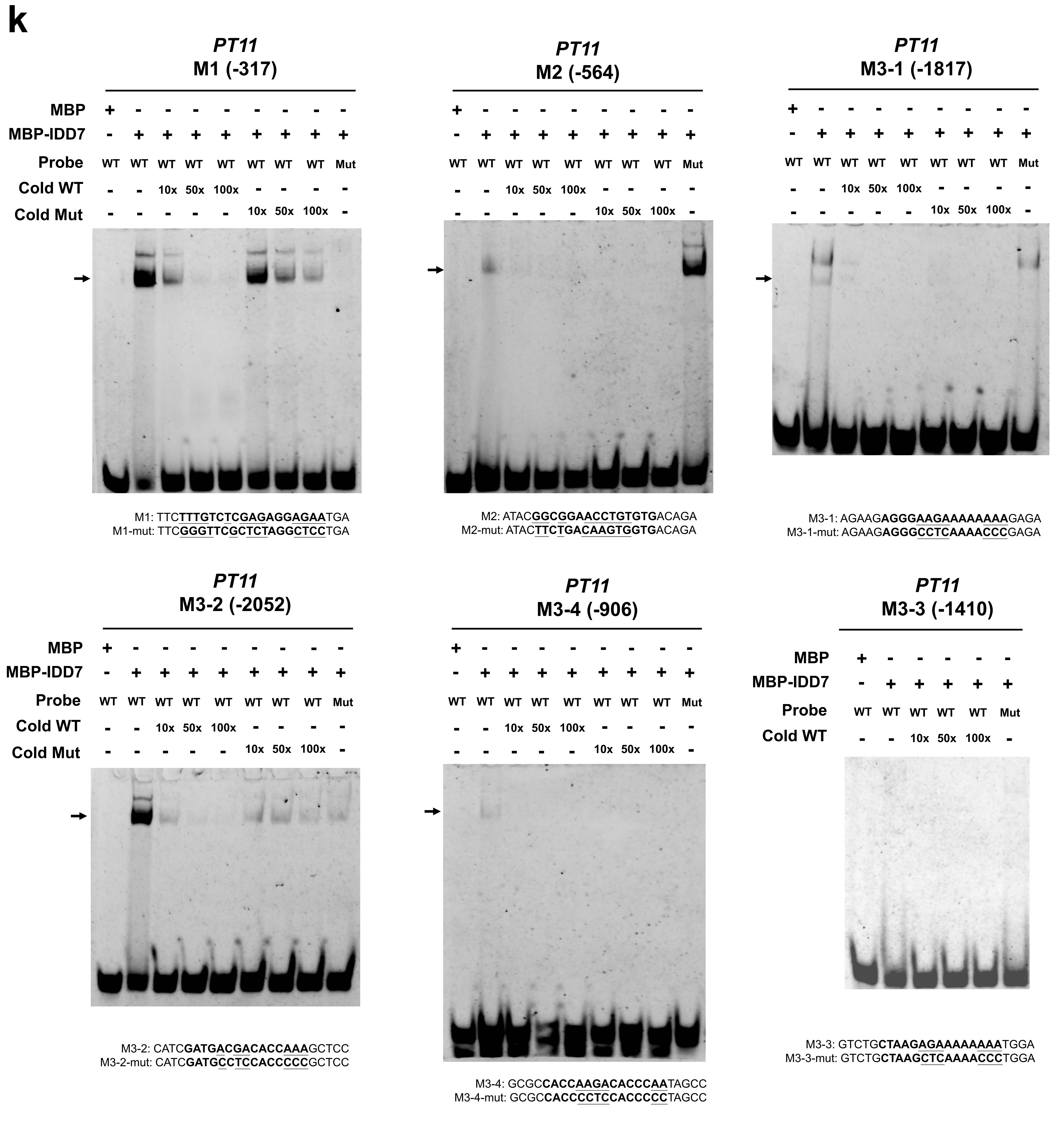

Supplementary Fig.9 (continued)

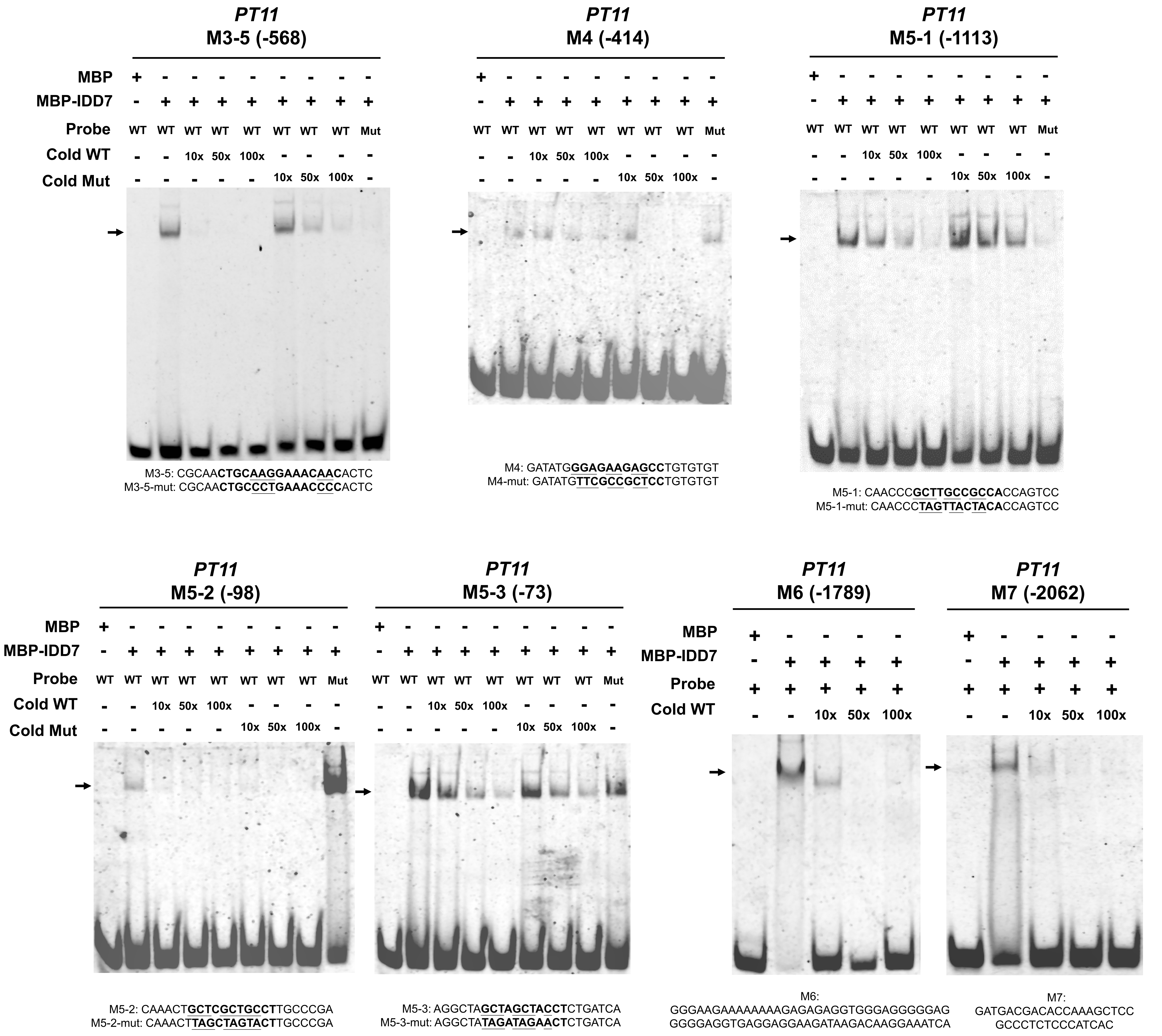

Supplementary Fig.9 (continued)

l

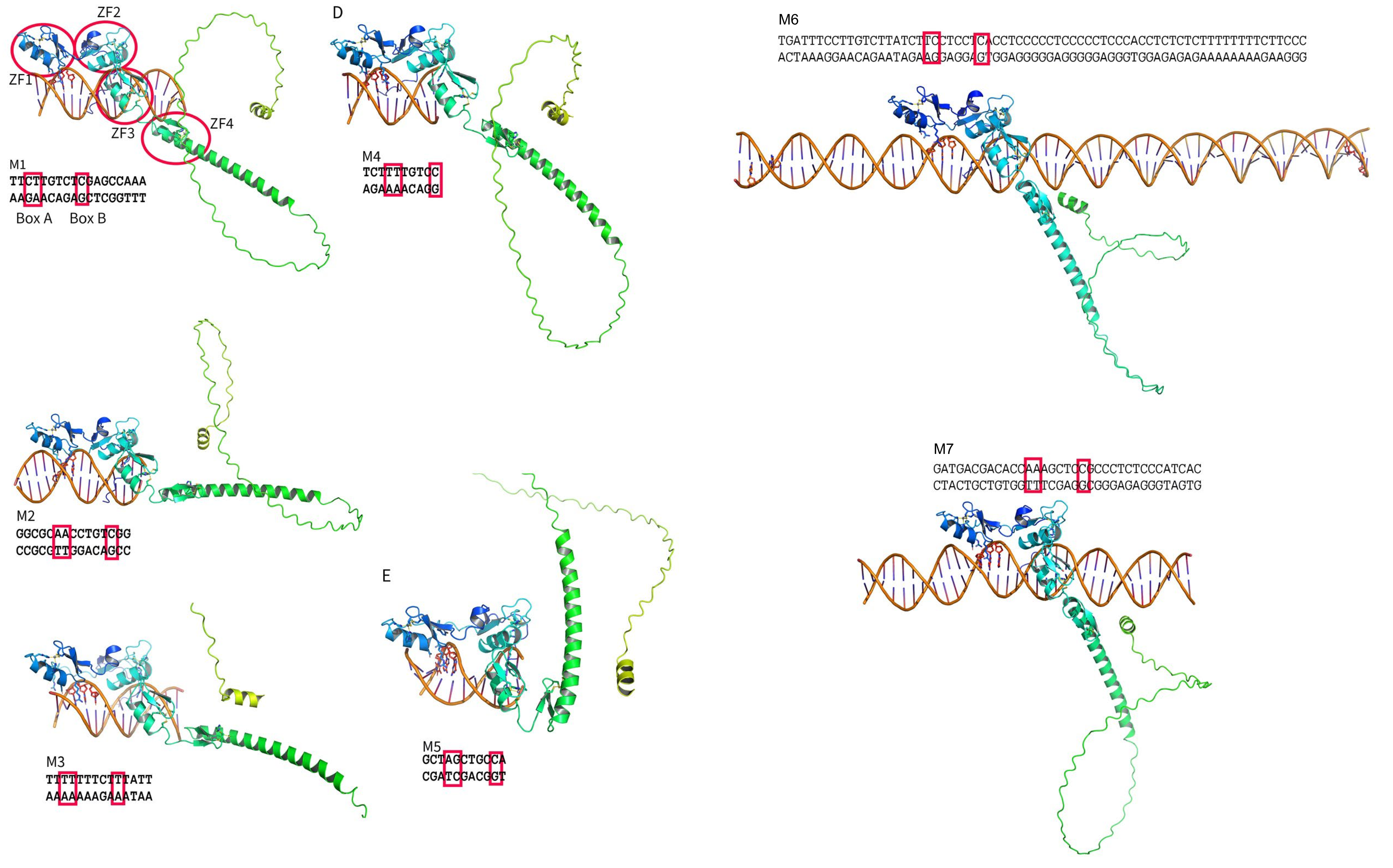

Supplementary Figure 9. Genome-wide identification of IDD7 targets by DAP-seq, and IDD7 binds probes containing M1–M4 motifs on the *PHR2* promoter and probes containing M1–M7 motifs on the *PT11* promoter. Bimodal dual-groove DNA recognition by IDD7 is shown. a Venn diagram depicting the overlap between the two DAP-seq replicates. **b** Distribution of IDD7 binding sites in the *Oryza sativa* genome. **c** IDD7 binding sites located in the promoter region were enriched near the transcription start site (TSS) compared with those in the GFP control. RPKM, reads per kilobase per million. TES, transcription end site. **d** Venn diagram depicting the overlap between genes bound by IDD7 at their promoter, genes downregulated in the *idd7* mutant, and the AM-related gene list. **e** IGV browser view of IDD7 binding to its target genes *PT11* and *RAM1*. Tracks show data from two GFP control samples and two IDD7 replicate samples. Gray boxes represent the gene model. The direction of transcription is indicated by the black arrow and the arrowheads within the gene model. **f** The heatmap depicts the fold enrichment of selected AM-related genes that might be bound by IDD7 at their promoters. The fold enrichment is relative to that of the GFP control. **g** Possible motifs were predicted by MEME. Rep A, IDD7 replicates A relative to GFP replicates A; Rep B, IDD7 replicates B relative to GFP replicates B. GFP was used as the negative control. Schematic representations showing the locations of DAP-seq-identified motifs: **h** M1–M4 on the 2000-bp *PHR2* promoter, and **j** M1–M5 on the 2300-bp *PT11* promoter. Motifs allowing one mismatch or gap/deletion every 6–10 bases were identified. To identify the M1 motif on the *PHR2* promoter and M6/M7 motifs on the *PT11* promoter, one mismatch or gap/deletion was allowed for every four bases. EMSA probes contained the corresponding motifs along with flanking sequences, resulting in a total length of 24 bps. **i-k** Results of electrophoretic mobility shift assays (EMSAs). MBP and MBP-IDD7 were expressed in *E. coli BL21*. Wild-type (WT) and mutated (Mut) probes were synthesized and labeled with FAM at the 5' end, whereas unlabeled WT probes (cold WT) and mutated probes (cold Mut) were used as competitors. For M6 and M7, only WT competitor assays are shown because these motifs were identified using a permissive sequence-matching threshold and lack a clearly defined short core sequence suitable for motif-specific mutation. Underlined bases indicate nucleotides in the probe that match conserved positions of the MEME-derived consensus motif and were mutated in EMSA assays. The black arrows indicate the expected shift locations. Representative EMSA results are shown; the experiments were independently repeated twice with similar results. **l** Bimodal dual-groove DNA recognition by IDD7. Molecular modeling and atomic interaction profiling of IDD7-DNA complexes reveal a strategy where the transcription factor interrogates both the major and minor grooves of the DNA helix. The structural model displays the core region containing four zinc finger (ZF) domains (residues 33–199); the disordered N-terminal (residues 1–32) and C-terminal (residues 306–492) regions as well as the models in the panel M3 and M5 with long coiled region are omitted for clarity. Key residues ARG77 (ZF1 domain) and ARG179 (ZF4 domain) are highlighted. DNA sequences M1 through M7 are shown adjacent to the structural models. Red Box A (left) depicts non-canonical minor groove binding by ARG77, which penetrates the narrowed groove of AT-rich sequences to establish electrostatic and hydrogen bonds with adenine and thymine bases. Red Box B (right) illustrates canonical major groove recognition by ARG179, where the guanidinium side chain anchors to G-rich core motifs by forming hydrogen bonds with guanine bases. This dual-reader architecture enables IDD7 to robustly identify diverse promoter elements (M1–M7) despite sequence variations. Ribbon diagram is generated by PyMOL3.1 (Schrödinger, LLC.).

Supplementary Fig.10

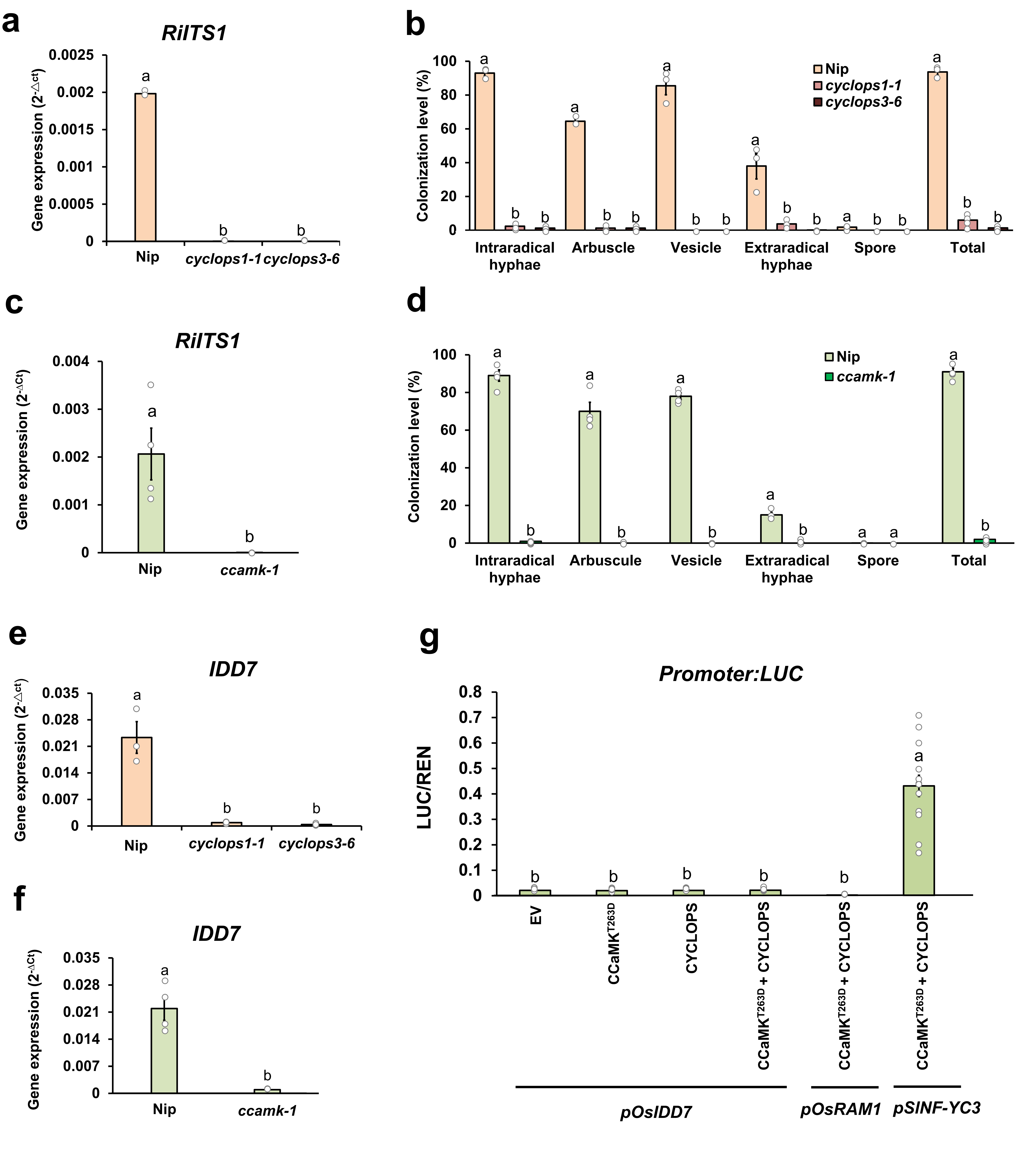

Supplementary Figure 10. *IDD7* expression is compromised in *cyclops* and *ccamk* mutants, but autoactive CCaMK and CYCLOPS do not activate the *IDD7* promoter. a *RiITS1* expression and **b** AM colonization level in the *cyclops* mutant. **c** *RiITS1* expression and **d** AM colonization level in the *ccamk* mutant. **e-f** *IDD7* expression in *cyclops* and *ccamk* mutants. Nipponbare (Nip), the *cyclops* mutant and the *ccamk* mutant were inoculated with the AM fungus *R. irregularis* (Ri), grown under 25 μM Pi conditions and harvested at 7 weeks postinoculation (wpi). Gene expression in the roots was analyzed by qRT–PCR and normalized to that of *Cyclophilin2* (*CYC2*) ($2^{-\Delta Ct}$). Data are presented as mean ± SEM. Each biological replicate consisted of roots pooled from two independently grown plants. For panels a, b and e, n = 3 biologically independent replicates for Nip; n = 4 biologically independent replicates for all other groups. For panels c, d and f, n = 4 biologically independent replicates. **g**, Transactivation assay by DLR in *N. benthamiana* leaves transiently coexpressing the reporter construct containing *pOsIDD7* (2043 bp), *pOsRAM1* (3000 bp) or *pSlNF-YC3* (2137 bp)*: LUC* (firefly luciferase) with *p35S: REN* (Renilla luciferase) and effector constructs, including EV (empty vector), CCaMK^{T263D} and CYCLOPS. Autoactive CCaMK^{T263D} is a truncated CCaMK with 1–298 amino acids consisting of a T263D point mutation, which is a phosphomimetic form of CCaMK, and an NLS sequence (CCaMK$^{298\text{-}T263D}$-NLS). Data are presented as mean ± SEM (n = 14 leaf discs from 14 independently infiltrated leaves per treatment, with one or two leaves per plant used for a given treatment). Different letters represent significant differences ($P < 0.05$, one-way ANOVA followed by a least significant difference post hoc test); exact P values for the post hoc pairwise comparisons are provided in the Source Data.

Supplementary information

Supplementary Table 1. RNA-seq data of all AM-induced rice IDDs

Supplementary Table 2. Primers for genotyping PCR

Supplemental Table 3. Primers used for qRT–PCR

Supplementary Table 1. RNA-seq data of all AM-induced rice IDDs

		Log$_2$ fold change (+AM vs -AM, wild-type)		
Locus number	Gene	Shi et al., 2021	Das et al., 2022	Shi et al., 2022
LOC_Os10g28330	ID1	Not DEG[a]	Not DEG	Not DEG
LOC_Os03g10140	IDD1	Not DEG	Not DEG	Not DEG
LOC_Os01g09850	IDD2	Not DEG	Not DEG	Not DEG
LOC_Os09g38340	IDD3	Not DEG	Not DEG	Not DEG
LOC_Os02g45054	IDD4	Not DEG	0.601267993	Not DEG
LOC_Os07g39310	IDD5	-1.03458	Not DEG	Not DEG
LOC_Os08g44050	IDD6	Not DEG	Not DEG	Not DEG
LOC_Os02g31890	IDD7	12.19165621	4.69239256	1.870178
LOC_Os01g14010	IDD8	Not DEG	Not DEG	Not DEG
LOC_Os01g70870	IDD9	Not DEG	2.717955666	3.89855287
LOC_Os04g47860	IDD10	Not DEG	0.761623976	Not DEG
LOC_Os01g39110	IDD11	1.292528711	-0.812144689	Not DEG
LOC_Os08g36390	IDD12	Not DEG	Not DEG	Not DEG
LOC_Os09g27650	IDD13	Not DEG	2.111811973	Not DEG
LOC_Os03g13400	IDD14	Not DEG	3.019131458	Not DEG

[a] Not DEG: The Log$_2$fold change value was not shown as it does not meet the designated DEG cut-off thresholds adopted in the corresponding publications, as listed below: Shi, 2021: |Log$_2$ fold change| > 1, p < 0.001; Das, 2022 : |Log$_2$ fold change| ≥ 0.5845, FDR ≤ 0.05; Shi, 2022: |Fold change| > 2, p < 0.001

Supplementary Table 2. Primers for genotyping PCR

Target	Direction	Primer sequence (5' to 3')
idd7 wild-type allele	Forward	AAGTTGCTTCCATGCGAGAT
	Reverse	CAAATGGGTTTTTCCAATGC
idd7 mutant allele	Forward	TCGTTAAAACTGCCTGGCACAGC
	Reverse	CAAATGGGTTTTTCCAATGC
IDD7 promoter: GUS line	Forward	CCAAACGTAAAACGGCTTGT
	Reverse	ACCCACAGGTGCTAATGACG
Empty vector of promoter: GUS line	Forward	CTACACAGCCATCGGTCCA
	Reverse	GTGCTTGACATTGGGGAGTT
IDD7 complementation EV line (*idd7*^{EV})	Forward	GCATGATGACCACCGATATG
	Reverse	ACACATGAGCGAAACCCTAT
IDD7 complementation line (*idd7*^{IDD7})	Forward	ATCATGAGGCCAGGTACTGTG
	Reverse	ACACATGAGCGAAACCCTAT
ccamk-1	Forward	TGTTCTCTTCCACAAAAGACACAT
	Reverse	CAGCAACGATGTAGATGGTCAAGC
cyclops-1	Forward	AGGCATTTTCATCACCCATC
	Reverse	ATTGTTAGGTTGCAAGTTAGTTAAGA
cyclops-3	Forward	ATTGTTAGGTTGCAAGTTAGTTAAGA
	Reverse	ATGCTGTACCAAGCCAAACC
pPHR2:GUS line (Azy$^{pPHR2:GUS}$ *and idd7*$^{pPHR2:GUS}$*)*	Forward	TTCGGTCGGGTTCGTTGG
	Reverse	GAACTTCAGGGTCAGCTTGC

Supplemental Table 3. Primers used for qRT–PCR

ID	Gene name or function	Primer sequence (5' - 3')
DQ028775	*RiITS1*	F: GAGACCATGATCAGAGGTCAGGT<br>R: GGTCATTTAGAGGAAGTAAAAGTCGTAAC
CYC2	*LOC_ Os02g02890*	F: GTGGTGTTAGTCTTTTTATGAGTTCGT<br>R: ACCAAACCATGGGCGATCT
IDD7	*LOC_Os02g31890*	F: CACACACCGAGCCTTCTGT<br>R: CTGTTGACCGTGCAGACTTC
PT11	*LOC_Os01g46860*	F: CATATCCCAGATGAGCGTATCATG<br>R: GAGAAGTTCCCTGCTTCAAGCA
RAM1	*LOC_Os11g31100*	F: CCCTACTTCTTGGGCAGGTT<br>R: CACTGCTCCACCTTCATCCT
WRI5a	*LOC_Os06g05340*	F: GAAGGAAGAGAGAGCCATCG<br>R: TCTTGTTAGCCGCATTGCTA
PHR2	*LOC_Os07g25710*	F: AGCTTGTACTGAAACTCAACCAC<br>R: AGCTGATTGGTGAACTGCGA
FatM	*LOC_Os01g31760*	F: ATCTTCGTCCGAGCAACCAG<br>R: ATTTCGTCCCTGACCTCCTT
NPF4.5	*LOC_Os01g54515*	F: CGTACCAGCAGAGCACAAAG<br>R: AGGCAAAAATGGTAGCAACAA
KIN2	*LOC_Os06g44430*	F: CAAGGCGGAGATGGAGTTC<br>R: GAGGTTCGGCATGTAGTCGT
CYCLOPS	*LOC_Os06g02520*	F: GGCAGAAGCAAAGGAAAGAA<br>R: CGCTCTTTTTCTTCCACCAG
CCaMK	*LOC_Os05g41090*	F: GATCTCATGGATGCAGAGGTCGTC<br>R: GCTCTCAGCACCTGCTCAAAC
SLR1	*LOC_Os03g49990*	F: GGAGGCCGTGCAGCAGGAGAACT<br>R: GCGGCGTCGAGGAGGGTGCTGTC
IDD4.3	*Loc_Os02g45054*	F: GTCTTGGGTTTGGGTGTTTG<br>R: GACAAGAAGCTGGGGAAAAA
IDD9	*Loc_Os01g70870*	F: AGTTAACACGAGGCGAGCTT<br>R: GGCATGGAACATTGGGATTT
IDD12	*LOC_Os08g36390*	F: TGCCAGATTGCTCTCCTTTT<br>R: AATCTGCCATGCGAGAGAAC
IDD13.1	*LOC_Os09g27650*	F: GCCGCCCCAGATATCATCAT<br>R: CGCCACACAATTGCATGCA
IDD14	*Loc_Os03g13400*	F: CGTGCACGCGTATGTATGTA<br>R: ACTTCATCAATTTGCGGCTA
