# Supplementary material for: OsIDD7 integrates signaling networks for arbuscular mycorrhizal symbiosis
Source: Nat Commun. 2026 Aug 31;17:10378. doi: 10.1038/s41467-026-77162-3 (PMC13627655; doi:10.1038/s41467-026-77162-3)
Supplement: Supplementary file 2 [file 41467_2026_77162_MOESM2_ESM.pdf]

Description of Additional Supplementary Files

File Name: Supplementary Data 1

Description: idd7 mutant RNA-seq.

File Name: Supplementary Data 2

Description: List of enriched IDD7-binding peaks identified with DAP-seq.

File Name: Supplementary Data 3

Description: Primers for construct generation.

File Name: Supplementary Data 4

Description: EMSA probes from publications.

File Name: Supplementary Data 5

Description: EMSA probes from the DAP-seq analysis.
