# Supplementary material for: OsIDD7 integrates signaling networks for arbuscular mycorrhizal symbiosis
Source: Nat Commun. 2026 Aug 31;17:10378. doi: 10.1038/s41467-026-77162-3 (PMC13627655; doi:10.1038/s41467-026-77162-3)
Supplement: Supplementary file 3 [file 41467_2026_77162_MOESM8_ESM.pdf]

nature portfolio

Corresponding author(s): Shu-Yi Yang

Last updated by author(s): Aug 12, 2026

Reporting Summary

Nature Portfolio wishes to improve the reproducibility of the work that we publish. This form provides structure for consistency and transparency in reporting. For further information on Nature Portfolio policies, see our Editorial Policies and the Editorial Policy Checklist.

Statistics

For all statistical analyses, confirm that the following items are present in the figure legend, table legend, main text, or Methods section.

n/a	Confirmed	
☐	☒	The exact sample size (*n*) for each experimental group/condition, given as a discrete number and unit of measurement
☐	☒	A statement on whether measurements were taken from distinct samples or whether the same sample was measured repeatedly
☐	☒	The statistical test(s) used AND whether they are one- or two-sided<br>*Only common tests should be described solely by name; describe more complex techniques in the Methods section.*
☒	☐	A description of all covariates tested
☐	☒	A description of any assumptions or corrections, such as tests of normality and adjustment for multiple comparisons
☐	☒	A full description of the statistical parameters including central tendency (e.g. means) or other basic estimates (e.g. regression coefficient) AND variation (e.g. standard deviation) or associated estimates of uncertainty (e.g. confidence intervals)
☐	☒	For null hypothesis testing, the test statistic (e.g. *F*, *t*, *r*) with confidence intervals, effect sizes, degrees of freedom and *P* value noted<br>*Give P values as exact values whenever suitable.*
☒	☐	For Bayesian analysis, information on the choice of priors and Markov chain Monte Carlo settings
☒	☐	For hierarchical and complex designs, identification of the appropriate level for tests and full reporting of outcomes
☒	☐	Estimates of effect sizes (e.g. Cohen's *d*, Pearson's *r*), indicating how they were calculated

Our web collection on statistics for biologists contains articles on many of the points above.

Software and code

Policy information about availability of computer code

Data collection	No custom software or code was used for data collection. Data collection was performed using standard commercial software provided with the instruments, including LAS X, ZEN Black software (v7.0) and ImageJ. JS (v0.6.0) for microscopy imaging. Experimental data were recorded manually and managed using Microsoft Excel 365.
Data analysis	No custom software or code was used for data analysis. Statistical analyses and data visualization were performed using standard commercial software, including R Studio software (v4.4.1) and Microsoft Excel 365.

For manuscripts utilizing custom algorithms or software that are central to the research but not yet described in published literature, software must be made available to editors and reviewers. We strongly encourage code deposition in a community repository (e.g. GitHub). See the Nature Portfolio guidelines for submitting code & software for further information.

Data

Policy information about availability of data

All manuscripts must include a data availability statement. This statement should provide the following information, where applicable:

- Accession codes, unique identifiers, or web links for publicly available datasets
- A description of any restrictions on data availability
- For clinical datasets or third party data, please ensure that the statement adheres to our policy

The RNA-seq and DAP-seq datasets generated during this study have been deposited in the NCBI Gene Expression Omnibus (GEO) under accession number GSE326810 and GSE327762, respectively. All other data supporting the findings of this study are available within the paper and its Supplementary Information.

April 2023

Source data are provided with this paper.

Research involving human participants, their data, or biological material

Policy information about studies with human participants or human data. See also policy information about sex, gender (identity/presentation), and sexual orientation and race, ethnicity and racism.

Reporting on sex and gender	Not applicable
Reporting on race, ethnicity, or other socially relevant groupings	Not applicable
Population characteristics	Not applicable
Recruitment	Not applicable
Ethics oversight	Not applicable

Note that full information on the approval of the study protocol must also be provided in the manuscript.

Field-specific reporting

Please select the one below that is the best fit for your research. If you are not sure, read the appropriate sections before making your selection.

- [x] Life sciences
- [ ] Behavioural & social sciences
- [ ] Ecological, evolutionary & environmental sciences

For a reference copy of the document with all sections, see nature.com/documents/nr-reporting-summary-flat.pdf

Life sciences study design

All studies must disclose on these points even when the disclosure is negative.

Sample size	Sample sizes were determined based on common practice in plant molecular biology research, and no statistical methods were used to pre-determine sample sizes. Each experiment was performed with at least 3 independent biological replicates. The final sample size (n) for each group, after any exclusion of outliers, is specified in the respective figure legends. These sample sizes provided sufficient statistical power to detect significant differences ($p<0.05$).
Data exclusions	Outliers were identified as samples deviating more than 2 standard deviations from the mean and were excluded to ensure the representative nature of the data. The final sample sizes (n) for each experiment are specified in the respective figure legends.
Replication	All experiments were performed with at least 3 independent biological replicates. Experimental findings were consistently reproduced across independent sittings, and all attempts at replication were successful.
Randomization	Plants were randomly assigned to experimental groups. To account for potential micro-environmental effects, the placement of plant pots within growth chambers was randomized.
Blinding	Blinding was not possible during treatment application; however, data analysis was performed using objective, standardized criteria to minimize potential bias.

Reporting for specific materials, systems and methods

We require information from authors about some types of materials, experimental systems and methods used in many studies. Here, indicate whether each material, system or method listed is relevant to your study. If you are not sure if a list item applies to your research, read the appropriate section before selecting a response.

Materials & experimental systems

n/a	Involved in the study	
[ ]	[x]	Antibodies
[x]	[ ]	Eukaryotic cell lines
[x]	[ ]	Palaeontology and archaeology
[x]	[ ]	Animals and other organisms
[x]	[ ]	Clinical data
[x]	[ ]	Dual use research of concern
[ ]	[x]	Plants

Methods

n/a	Involved in the study	
[x]	[ ]	ChIP-seq
[x]	[ ]	Flow cytometry
[x]	[ ]	MRI-based neuroimaging

Antibodies

Antibodies used	For western blot of DLR assay, BiFC and LCI, anti-Myc (M4439-100UL), anti-FLAG (F1804-50UG) (both 1:1000; Sigma-Aldrich, St. Louis, MO, USA), anti-His (1:5000; HIT001M, BIOMAN, New Taipei City, Taiwan), anti-nYFP (1:2000; AS11 1776, Agrisera, Vännäs, Sweden), anti-cYFP (1:10000; AS11 1775, Agrisera, Vännäs, Sweden), anti-n/c-LUC (1:4000; L0159, Sigma-Aldrich, USA), anti-actin (1:4000; AS132640, Agrisera, Vännäs, Sweden), HRP-conjugated anti-mouse (1:4000; E-AB-1001, Elabscience, Wuhan, China), and anti-rabbit (1:10000; ab6721, Abcam, Cambridge, UK) antibodies were used. For co-IP, anti-GFP (1:10000; ab290-50UL, Abcam, Cambridge, UK), anti-FLAG (1:4000; F1804-50UG, Sigma-Aldrich, St. Louis, MO, USA), anti-MYC (1:2500; M4439-100UL, Sigma-Aldrich, St. Louis, MO, USA), anti-His (1:5000; HIT001M, Bioman, New Taipei City, Taiwan), HRP-conjugated anti-mouse (1:10000; E-AB-1001, Elabscience, Wuhan, China), and Clean-Blot IP Detection Reagent (1:1000; 21230, Thermo Fisher, Massachusetts, USA) were used.
Validation	All antibodies used in this study are commercially available and have been validated by the manufacturers for the specific applications. Validation data and representative images are provided on the manufacturers' websites.

Plants

Seed stocks	Seed stocks for the T-DNA insertion mutants (3A-04543R) were obtained from the Postech, Korea. Transgenic lines generated in this study are maintained in our laboratory's seed collection. All seed stocks are available from the corresponding author upon reasonable request. Detailed information on the background and generation of these stocks is provided in the Methods section.
Novel plant genotypes	Detailed information on the generation and validation of the novel plant genotypes (including transgenic lines and T-DNA mutants) is provided in the Methods section and Supplementary Figures. All seeds/lines will be made available to the research community upon reasonable request. Where applicable, T-DNA insertion lines were obtained from the Postech (Korea) under their respective accession numbers as listed in the Methods.
Authentication	The identity of all seed stocks was authenticated by PCR genotyping and phenotypic observation in each generation.
