# Supplementary material for: OsIDD7 integrates signaling networks for arbuscular mycorrhizal symbiosis
Source: Nat Commun. 2026 Aug 31;17:10378. doi: 10.1038/s41467-026-77162-3 (PMC13627655; doi:10.1038/s41467-026-77162-3)
Supplement: Supplementary file 4 [file 41467_2026_77162_MOESM9_ESM.pdf]

nature portfolio

Peer Review File

OsIDD7 integrates signaling networks for arbuscular mycorrhizal symbiosis

Corresponding Author: Dr Shu-Yi Yang

This file contains all reviewer reports in order by version, followed by all author rebuttals in order by version.

Version 0:

Reviewer comments:

Reviewer #1

(Remarks to the Author)
The authors identify a previously uncharacterized transcription factor required for rice root colonization by arbuscular mycorrhiza (AM) fungi. They propose that this AM-induced IDD7 protein interacts with the phosphate starvation response regulator PHR2 the proteolytic target of gibberellin signaling DELLA/SLR1 to regulate downstream genes involved in arbuscular mycorrhiza symbiosis such as the transcription factors RAM1, WRI, required for arbuscule development and PT11 required for phosphate uptake from fungal arbuscules. The topic is timely, and several observations are exciting. However, the central conclusions are overstated and require more experimental evidence. In addition, the writing needs major improvement for clarity and precision.

Major concerns
1) Reduced AM marker gene induction in idd7 can be a consequence of reduced colonization, not direct IDD7 regulation: A key issue throughout the manuscript is that many “downstream targets” are defined by reduced expression in idd7 versus WT under AM conditions. Because idd7 shows reduced colonization, most AM-induced genes would be expected to show weaker induction as a secondary effect, because most of them are induced in a cell autonomous manner in colonized cells. This makes it difficult to interpret differential expression as evidence of direct IDD7-dependent transcriptional regulation. Bona fide target genes should be only declared if they are less expressed in idd7 mutants and IDD7 binds to their promoter.

2) The authors rely heavily on transient assays in Nicotiana and in vitro EMSA. These approaches are useful for hypothesis generation but are prone to artifacts. This concern is partly acknowledged by the authors themselves: DAP-seq did not recover binding sites previously inferred from EMSA (e.g., for PHR2, lines 251–257), raising questions about specificity and reproducibility.
To support the claim of direct regulation by IDD7, ChIP–qPCR/ChIP–seq for IDD7 binding to candidate promoters in rice would be ideal, or at least rice overexpression/inducible expression lines showing increased target gene expression.
The absence of pPHR2:GUS signal in arbuscule cells in idd7 is a stronger piece of evidence, but it addresses only one proposed target and does not explain the mutant phenotype (phr2 mutants reportedly do not show the same arbuscule defect).
Related to this, several statements should be toned down because they treat AM-downregulated genes in idd7 as IDD7 targets. For example:
Line 112: “...115 AM-related DEGs that were likely to be regulated by IDD7 (Fig. 3b).” Given reduced colonization, these genes are more plausibly “AM-regulated genes whose induction is reduced in idd7,” not necessarily IDD7 targets.
Line 18 (similar claim also in line 132): “IDD7 is involved in regulating genes involved in fatty acid biosynthesis, nutrient transport, and symbiotic signaling.”This appears based largely on GO enrichment and reduced induction of AM genes in idd7, which can be explained by reduced colonization. The authors should consistently incorporate colonization differences into their interpretation and avoid causal language unless supported by direct in vivo evidence. At a minimum these statements should be backed up by the DAP-seq data.

3) Promoter selection and controls (Line 138). The authors test transactivation for PHR2, PT11, RAM1, and WRI5a promoters, but the selection criteria are unclear given the broader DEG list (Fig. 3c). Please clarify how these promoters were chosen and how many additional candidates were tested but did not respond to IDD7.
Also, the Nicotiana assays would benefit from stronger negative controls to evaluate artifact rates. For example, choose promoters not downregulated in idd7 but containing similar putative IDD motifs, and test whether they are transactivated in

Nicotiana. This would help define specificity and reliability in this heterologous system. For all promoters IDD7 and PHR2 binding sites should be mutated as a specificity control.

4) Protein–protein interaction should be supported by orthogonal experiments.
The conclusion that IDD7 interacts with PHR2 and SLR1 is currently based only on BiFC and split-luciferase assays in Nicotiana, which are susceptible to false positives/negatives due to heterologous overexpression and even more so due to covalent binding between the two halfs of the fluoreophore/lucifease. Additional orthogonal interaction evidence is needed to substantiate these claims, for example Y2H assay or co-IP.
Problems: (1) Inconsistency between assays: e.g., Extended Data Fig. 6a suggests IDD7ΔTQDFLG does not interact with PHR2, whereas Extended Data Fig. 6f suggests the opposite (although the signal is weaker than with wild type PHR2). (2) It would be good if the authors could show the level of protein accumulation in both assays.
Line 150: The authors suggest that the PHR2–SLR1 interaction is weakened in the presence of IDD7, implying mutual exclusivity rather than a trimeric complex (Extended Data Fig. 6c).
Without protein level controls for all BiFC constructs in each condition (e.g., immunoblotting using anti-N-YFP/C-YFP tags), the loss/reduction of YFP could simply reflect altered abundance or stability of one fusion protein when co-expressed, as the leaf cells may be overloaded with production of three proteins.
Similarly, for the deletion analysis (Fig. 4), the authors infer that TQDFLG mediates interactions because IDD7ΔTQDFLG shows no YFP signal. This conclusion requires evidence that the deletion does not reduce protein stability/accumulation.
Finally, please clarify how interaction "strength" (YFP intensity) is quantified: Are the normalization markers (e.g., NLS-mCherry) on the same plasmid? Are constructs delivered via separate Agrobacteria strains, and if so, how is the efficiency of transformation controlled? Ideally, the authors should normalize YFP to a co-expressed marker from the same plasmid to minimize variability from gene dosis (different amounts of plasmids per cell) and expression differences.

5) Transactivation assay results in Nicotiana are confusing and suggest indirect effects
In earlier figures (e.g., Fig. 5), the authors argue that IDD7 regulates PHR2 and PT11 because IDD7–VP16 and IDD7 + SLR1 transactivate these promoters. However, later results show that IDD7–VP16 can still activate promoters even when all putative IDD binding sites are removed. This weakens the claim that promoter activation depends on direct binding to those motifs and raises the possibility of indirect activation through other TFs in the transient system.
Given this, it would be better to support claims of direct DNA binding and direct regulation in vivo binding evidence such as ChIP–qPCR/ChIP–seq or cut and tag, and the transactivation assays in Nicotiana leaves should be interpreted with care.

6) Other major points
Line 68 / Fig. S1 (phylogeny): The IDD7 phylogenetic tree appears unusual with all branches at the same level. Please verify the tree quality (e.g., inclusion of known IDD family members clustering as expected; alignment quality; appropriate model/method). Also please mention gene identifiers, not simply the names, as they are often inconsistent across publications.
Figure callouts: numerous statements in the text lack clear figure references, making the manuscript difficult to follow. Please add figure citations consistently (e.g., "(Fig. X)" or "(Extended Data Fig. X)"). Examples: Line 210: "its coexpression with SLR1 or PHR2 was similar to that of full length IDD … (Fig. ?)" Line 225: "with P4 being the strongest (Fig. ?)"

Data presentation: For bar charts especially where differences are modest, please show all datapoints.

Line 203–208: Even if PHR2 and IDD7 interact, overlap in downstream gene changes between mutants does not necessarily support or refute interaction because they could act redundantly or in parallel. The authors should avoid overinterpreting expression overlap patterns.

Line 209: It is surprising that IDD7ΔTQDFLG activates PT11 alone. This could reflect altered protein stability/abundance. Do the authors see differential accumulation of IDD7 vs. IDD7ΔTQDFLG on a Western Blot?

Lines 174-214: This part is difficult to follow with different details for different promoters. Rephrasing for clarity is recommended.

Lines 216–234; Figs. S8–S9: The EMSA section is difficult to follow and needs clearer presentation. This section is currently very hard to read because many similar short sequences are referenced without a clear organizing framework. Suggestions: Provide a summary table listing each probe (P1, P2, etc.) with the full 24 bp probe sequence (including flanks), and indicate which bases are mutated. Use a sequence logo to show degenerate positions and motif variability, rather than listing multiple motifs only in text. Clearly define "P motifs" when first introduced (it initially was unclear what P6 refers to around line 222). In Figs. S8/S9, avoid labeling probes only with short cores (e.g., "TTTGTG") when multiple probes share similar cores. it becomes impossible to reconcile why binding differs.

Figure 5c: There is large variation in the basal expression of PHR2 promoter version. Can this be explained?

Extended data Figure 8: All EMSAs are lacking one important specificity control, which is the competition between the labelled WT probe with 10x, 50x and 100x of the unlabelled mutated probe. If the binding is specific the mutated unlabelled probe should not or hardly compete with the labelled probe. Furthermore, can IDD7 binding to the "mutated" P9 and P10 should be explained?

Line 285: It is unclear how EMSA probe sequences can be deleted. It would be better to use precise language and name the

cis-element that was deleted. Generally deletion of cis-elements can change the structure or promoters, therefore mutation of cis-elements is preferable.

Target validation (Lines 297–303). The authors validate pPHR2 but claim many additional downstream targets. Given that major conclusions rely heavily on heterologous transient overexpression assays, additional evidence in rice (as with pPHR2 in idd7) for more proposed targets would substantially strengthen the work. Did the authors test additional candidate promoters/targets in the native context?

CCaMK–CYCLOPS pathway interpretation (Line 305; Fig. S10)

Line 305: The current section does not demonstrate that IDD acts downstream of CCaMK–CYCLOPS. IDD7 expression could reflect colonization status rather than CCaMK–CYCLOPS regulation. This interpretation should be revised. Furthermore, CCaMK T265D is known to induce spontaneous nodules in legumes but it is not known to activate the RAM1 promoter together with CYCLOPS. In Pimprikar et al the kinase-only version of CCaMK (CCaMK 314) was used. Therefore, the data cannot be directly compared.

Line 335: “...supports a model in which PHR2 acts through IDD7-bound DNA...”
This is not strictly supported. The requirement for both factors in a reporter assay could also reflect indirect effects via downstream TFs induced by PHR2. The authors should rephrase more cautiously unless direct binding and mechanism are demonstrated.

Fig. S7c: Please explain the contrasting pCCaMK::LUC patterns under different factor combinations.

Minor points

In several instances the author write that genes are ‘downregulated’ in the idd7 mutant. This seems to be a mis-conception as they are likely not actively downregulated but ‘less expressed’ either due to lower colonization in the mutant or the absence of induction by IDD7.

Line 71: Baseline pIDD7:GUS expression under mock conditions is important; consider moving this to a main figure.

Line 80: Please state the length of the native promoter used and which terminator was used in the complementation construct.

Line 85: Please define RiITS1 when first mentioned. Furthermore, ‘upregulated’ would entail that the gene expression per fungal unit is increased. In reality the increased RiITS1 expression likely reflects increased colonization and this should be phrased as such.

Line 87: The two complementation lines show dramatically different expression levels of IDD and downstream genes; please discuss why. An additional independent line to support conclusions about native promoter functionality could be helpful.

Use of two WT controls (Dongjin and an azygous line) is confusing, although not incorrect. For Fig. 2c (and elsewhere), clarify which control is the appropriate comparator for idd7 and why.

Line 101: This headline seems incomplete. Did the authors mean “IDD7 regulates genes involved in nutrient exchange and signaling”? The same is true for the headline in line 146, 174, 216, 305.

Line 102: What do the authors mean with ‘downstream role’: Should this read: ‘role in regulating downstream genes’?

Line 178: How does SLR1 activate the CYCLOPS promoter alone? This should be discussed as GRAS factors are generally known not to bind DNA.

Line 200 (Extended Data Fig. 7c): If IDD7–VP16 can transactivate promoters lacking IDD binding sites, interpretations of “IDD7 requires a partner to activate this promoter” should be reconsidered.

Line 221: Why are TTGTCT (5′-AGACAA-3′), AGACAG, and TTTGGT in the PHR3 promoter specifically mentioned? Are these established IDD binding sites?

Please clarify motif differences and binding outcomes between AGACAG in PHR2 (Fig. S8 P7/P8) and PT11 (Fig. S8d P6), and between TTTGGT (Fig. S8d P7) and probes with mobility shifts (e.g., Fig. S8d P9/P10; Fig. S8b P9). A sequence logo would help readers understand which positions are truly informative.

Line 265–271: If a mutated IDD7 shows auto-activation, did the authors test whether the mutation also alters DNA-binding properties?

Line 276: “in which IDD motifs defined using a 4–10 bp mismatch/gap tolerance were deleted” needs grammatical correction (and similar phrasing elsewhere should be edited).

Discussion and Fig. 6: The authors state that CCaMK and CYCLOPS are expressed only in the epidermis. Has this been shown in rice?

Reviewer #2

(Remarks to the Author)
The manuscript by Chen et al. reports the identification of OsIDD7 as a novel key transcription factor regulating AM symbiosis in rice. They show that IDD7 controls arbuscule development together with OsSLR1 and OsPHR2, both of which can interact with IDD7 through its TQDFLG domain. Multiple cis-regulatory elements bound by IDD7 were identified in the promoters of genes induced in arbuscule-containing cells, positioning IDD7 as a central coordinator of SLR1 and RAM2 mediated expression to ensure proper arbuscule development.

This original work adds an important novel component to the transcriptional network that controls arbuscule development at the heart of this important symbiosis. The manuscript is clearly written and the conclusions are largely support by the presented results.

Below I have several suggestions and comments. Especially, a confirmation of the suggested interactions in roots would strengthen the work.

According to Supplementary table 1, additional IDD genes in rice may show induced expression upon AMS. Did the authors test the expression of other IDD genes by qPCR as they did for IDD7?
Is the arbuscule specific expression of IDDs also supported by public available single cell data?

IDD proteins have been shown to be mobile. It would be good to check the localization of an IDD7 GFP fusion.

Briefly explain the Azygous control in the text (line 103).

OsRAM1 did not interact with OsIDD7 in the BiFC assay. Did the authors check the expression of RAM1 at the protein level in Nicotiana leaves, to make sure that it was properly expressed?

It would be good to verify the interactions in roots using co-IP to confirm that the interactions truly occur in the relevant rice tissue.

Line 246, explain IGV.

Line 275, clarify ΔPub-IDDBM.

Line 371, maybe indicate more clearly that this is based on predictions. No validation experiments are presented.

In the final model (Fig. 6) the negative regulation of CCaMK expression by IDD7 is indicated. This is based on the transactivation in Nicotiana leaves. Given a lack of verification of this downregulation in arbuscule containing cells (or rice roots), I would suggest to remove this speculative result from the model.

Version 1:

Reviewer comments:

Reviewer #1

(Remarks to the Author)
The manuscript has been improved however, some of the data (e. g. the phylogenetic tree and EMSAs) and also the writing have not yet reached publication quality. Some figures stretch over multiple pages, which makes understanding them cumbersome. The authors could consider reducing the size of the panels to fit the figure onto one page or to divide the figure into several.
Comments: +
Line 42: Please note that Lotus CBX1 serves as critical regulator of nutrient exchange genes in complex with RAM1 (Paries et al 2025, PNAS). This should be mentioned in the introduction.
Figure 1b: The phylogenetic tree is practically not improved from the last submission and still contains many polytomies, which limits the usefulness of the tree. Especially the location of the protein of interest OsIDD7 in the phylogeny is ambiguous. I suggest to either remove the tree entirely (as in the current version it does not add useful information to the manuscript) or to fix the polytomies by 1) adding more data and 2) by rooting the tree. Especially adding more dicot sequences and at least a second Solanaceaous species, a second Brassicacae species and a second legume, may help to turn the polytomies into dichotomies.
Fig. 5 and Fig S5b: The transactivation of target promoters is quite poor and for some promoters not even 2-fold. I wonder

whether the chosen promoters are really bona fide targets and/or whether IDD7-VP16 or IDD7 in complex with PHR2 and SLR1 is sufficient to explain their induction.
The EMSAs are lacking an important control: The mutated cold probe in the same dilution series as the cold WT probe.
Fig. S9e, please indicate the direction of the genes.
Line 270: Binding strength can only be estimated by determining the Kd of the binding (e. g. by microscalte thermophoresis or similar methods). I suggest to rephrase for rigor: "The quantity of the shifted probe varied, potentially suggesting differences in binding strength…".
Line 313: The text should reflect that alpha-fold can only make predictions. E. g. the word 'reveal' should be replaced by 'predicted' etc.
Line 349-353: The transactivation of promoters from which all IDD7 binding sites have been mutated by IDD7-VP16 may indicate unspecific transactivation. This may potentially lead to the rejection of the hypothesis that IDD7 specifically regulates the chosen target promoters. This notion is supported by the relatively weak induction of promoters as compared to background activation in absence of IDD7. Alternatively, more TFs are required to strongly induce the promoters.
Line 372 and following: This is a misinterpretation. The assay in S10g cannot be interpreted because CYCLOPS phosphorylation by the truncated CCaMK only containing the kinase domain is required for induction of the RAM1 promoter and other AM-induced promoters. Furthermore, in this assay the EV control for most promoters except the IDD7 promoter are missing.
Overall, the manuscript text is written in a lengthy and partially repetitive manner. It would be good to revise it for better readability.

Minor comments:
Throughout the manuscript I suggest to use ‚arbuscule-containing cells' instead of 'arbusculated cells'.
Line 74-76: The reason for mentioning the tree here is not very clear, as it seems more intuitive to continue from with expression pattern analysis from qPCR to promoter:reporter assays. If there is a reason for describing the tree before the promoter:GUS assay this should be introduced/explained in a way that the reader can understand.
Line 81: Do the authors mean 'N. benthamiana leaves'?
Fig. 2C is very crowded and difficult to grasp. I suggest to separate the bars indicating fungal structures by a vertical dotted line (or similar).
Line 188: 'the complementation results' should be rephrased for readability. Do the authors mean: 'the results from the complementation experiment'?
Line 163: I am not sure if 'data not shown' is permitted by the journal. In any case if these promoters are used as negative controls it is important to show the data.
Line 184: I suggest to rephrase the sentence: "Therefore, the results suggest, that IDD7 may affect …" (do not conclusively demonstrate weakens the narrative unnessessarily, 'suggest' and 'may' is careful enough in my opinion).
Line 199: This statement is vage, describe which differences in protein level were observed.
Figure S7 is difficult to read because the transactivation assays with mutated promoters are far away from the respective WT promoters. For comparison it would be helpful to show WT and mutated promoters side-by-side.

Reviewer #2

(Remarks to the Author)
The revised manuscript by Chen and colleagues reveals an important novel role for rice IDD7 in the signaling network for arbuscular mycorrhizal symbiosis. They show that IDD7 can interacting with PHR2 and DELLA SLR1, to synergistacly regulate important genes for arbuscule development and functioning.
Although validation of interactions remains to be proven in the relevant rice root tissues the data make a strong case for the central role of OsIDD7 (and possibly additional IDDs). This work is the first to show the involvement of IDDs in the symbiosis and is expected to inspire a further unraveling of their role in other plant species as well.
In my view the authors have sufficiently addressed the concerns raised by the reviewers.

Open Access This Peer Review File is licensed under a Creative Commons Attribution 4.0 International License, which permits use, sharing, adaptation, distribution and reproduction in any medium or format, as long as you give appropriate credit to the original author(s) and the source, provide a link to the Creative Commons license, and indicate if changes were made.
In cases where reviewers are anonymous, credit should be given to 'Anonymous Referee' and the source.
The images or other third party material in this Peer Review File are included in the article's Creative Commons license, unless indicated otherwise in a credit line to the material. If material is not included in the article's Creative Commons license and your intended use is not permitted by statutory regulation or exceeds the permitted use, you will need to obtain permission directly from the copyright holder.
To view a copy of this license, visit https://creativecommons.org/licenses/by/4.0/

Title of manuscript: OsIDD7 integrates signaling networks for arbuscular mycorrhizal symbiosis
Manuscript number: NCOMMS-26-007876A

We are grateful for the suggestions and comments to improve our manuscript. Below are our point-by-point responses to the editor and reviewers' comments.

Answers to Referee #1

Referee #1-1

Reduced AM marker gene induction in idd7 can be a consequence of reduced colonization, not direct IDD7 regulation: A key issue throughout the manuscript is that many "downstream targets" are defined by reduced expression in idd7 versus WT under AM conditions. Because idd7 shows reduced colonization, most AM-induced genes would be expected to show weaker induction as a secondary effect, because most of them are induced in a cell autonomous manner in colonized cells. This makes it difficult to interpret differential expression as evidence of direct IDD7-dependent transcriptional regulation. Bona fide target genes should be only declared if they are less expressed in idd7 mutants and IDD7 binds to their promoter.

<u>Our response:</u>

We thank the reviewer for this important comment. We agree that reduced expression of AM-induced genes in *idd7* mutants could partially result from decreased fungal colonization. We have revised the manuscript to clarify this point and to avoid overinterpretation of RNA-seq data as evidence of direct regulation.

Specifically, we now distinguish between (i) genes showing IDD7-dependent expression changes and (ii) candidate direct targets supported by additional evidence. We have revised the text to indicate that many AM-related genes identified by RNA-seq may be indirectly affected.

Importantly, we provide additional evidence supporting direct regulation for a subset of genes. DAP-seq and EMSA assays demonstrate that IDD7 binds to the promoters of *PT11*, *RAM1*, and *PHR2*, and transactivation assays show that IDD7, together with SLR1 and/or PHR2, activates these promoters. Therefore, *PT11*, *RAM1*, and *PHR2* are now described as candidate direct targets of IDD7, whereas other genes are described more cautiously as IDD7-associated genes.

We have revised the Results section (Lines 113-152), Fig. 3 legend (Lines 1176-1177) and Fig. 6 (Lines 1233-1237) legend accordingly.

Referee #1-2

The authors rely heavily on transient assays in Nicotiana and in vitro EMSA. These

approaches are useful for hypothesis generation but are prone to artifacts. This concern is partly acknowledged by the authors themselves: DAP-seq did not recover binding sites previously inferred from EMSA (e.g., for PHR2, lines 251 – 257), raising questions about specificity and reproducibility.

To support the claim of direct regulation by IDD7, ChIP – qPCR/ChIP – seq for IDD7 binding to candidate promoters in rice would be ideal, or at least rice overexpression/inducible expression lines showing increased target gene expression.

Our response:

We thank the reviewer for this important and constructive comment. We agree that chromatin-based assays such as ChIP-qPCR, ChIP-seq, or CUT&Tag would provide valuable complementary evidence for in vivo binding of IDD7 to candidate target promoters in rice.

In the current study, we used multiple complementary approaches to investigate the regulatory activity of IDD7. EMSA assays showed sequence-specific binding of IDD7 to candidate promoter fragments, and transcriptional activation assays in *Nicotiana benthamiana* supported the ability of IDD7 to activate transcription from these promoters in a plant transient-expression system. However, in light of the reviewer's concern, we have revised the manuscript to interpret these data more cautiously and to avoid overstatement regarding definitive in vivo direct regulation.

We also explored the possibility of generating suitable materials for ChIP-based analyses. However, our unpublished data showed that constitutive overexpression of IDD7 or tagged IDD7 constructs was not suitable for this purpose. Specifically, overexpression of IDD7 or IDD7-EGFP failed to rescue the fungal developmental defects of the *idd7* mutant, and overexpression of IDD7 or 4xMyc-IDD7 in wild-type Nipponbare negatively affected AM symbiosis. These results suggest that proper IDD7 function requires tightly controlled spatial expression and that ectopic expression in non-arbusculated cells may interfere with AM development. At present, we have a functional complementation line (*pIDD7:IDD7* in the *idd7* mutant background) that rescues the mutant phenotype, but we do not yet have a suitable antibody or tagged functional line optimized for ChIP analysis. We are currently generating tagged complementation materials for future chromatin-based studies.

To further strengthen the manuscript in a homologous rice system, we have now added BiFC assays in rice protoplasts, which confirm the interaction of IDD7 with SLR1 and PHR2 in rice cells (Supplementary Fig. 6c, Lines 176-178). This result complements our previous interaction data obtained in *Nicotiana* and provides additional support for the physiological relevance of the IDD7–SLR1 and IDD7-PHR2 modules in rice.

Accordingly, in the revised manuscript we have:

1. softened the wording regarding "direct regulation" by IDD7 (Lines 288-290, 399-402,

469-473),
2. clarified the limitations of the transient transactivation system and the current lack of chromatin-level validation (Lines 310-312, 349-353, 477), and
3. added new interaction data in rice protoplasts to strengthen the mechanistic model in a homologous system (Lines 176-177).
We hope the reviewer agrees that the revised manuscript now provides a stronger and more balanced framework for understanding IDD7 function in AM symbiosis, while also clearly acknowledging the need for future chromatin-based validation of candidate target genes.

Referee #1-3
The absence of pPHR2:GUS signal in arbuscule cells in idd7 is a stronger piece of evidence, but it addresses only one proposed target and does not explain the mutant phenotype (phr2 mutants reportedly do not show the same arbuscule defect).
Our response:
We thank the reviewer for this insightful comment. We respectfully note that previous work has in fact reported arbuscule defects in *phr2* mutants. Specifically, Shi et al. (2021) showed that *Osphr2-1* mutants exhibit significantly reduced mycorrhizal colonization and impaired arbuscule development, including smaller arbuscules, at multiple time points (2, 4, and 6 weeks post-inoculation; Figures 3A–3D and S5A) (Shi et al. 2021). This supports a functional role for PHR2 in arbuscule development.
Consistent with this, our results show that *idd7* mutants display pronounced defects in arbuscule morphology, including degeneration and hyphal septation (Fig. 2h), as well as a significant reduction in arbuscule size (Fig. 2i and j). These observations indicate that IDD7 is required for proper arbuscule development and maintenance.
Importantly, while reduced *PHR2* expression and loss of *pPHR2:GUS* signal in *idd7* support that PHR2 acts downstream of IDD7, we agree that IDD7 likely regulates additional targets contributing to the mutant phenotype. Indeed, our binding and transactivation data indicate that IDD7 directly regulates multiple AM-related genes (e.g., *PT11* and *RAM1*), suggesting that the *idd7* phenotype arises from combined defects in multiple regulatory pathways rather than from PHR2 alone.
We have revised the manuscript to clarify this point (Lines 393-405).

Referee #1-4
Related to this, several statements should be toned down because they treat AM-downregulated genes in idd7 as IDD7 targets. For example:
Line 112: “...115 AM-related DEGs that were likely to be regulated by IDD7 (Fig. 3b).”
Given reduced colonization, these genes are more plausibly “AM-regulated genes

whose induction is reduced in idd7," not necessarily IDD7 targets.

<u>Our response:</u>

We thank the reviewer for this important comment and fully agree that reduced expression of AM-related genes in *idd7* mutants may partly result from decreased fungal colonization rather than direct transcriptional regulation by IDD7.

Accordingly, we have revised the manuscript to avoid overinterpretation of RNA-seq data. Specifically, we have replaced statements such as "genes regulated by IDD7" with more accurate descriptions, including "IDD7-dependent genes" or "AM-regulated genes whose induction is reduced in idd7." In the revised text, the sentence now reads: "...115 AM-related DEGs that were associated with IDD7-dependent expression changes…"(Lines 125).

Referee #1-5

Line 18 (similar claim also in line 132): "IDD7 is involved in regulating genes involved in fatty acid biosynthesis, nutrient transport, and symbiotic signaling." This appears based largely on GO enrichment and reduced induction of AM genes in idd7, which can be explained by reduced colonization. The authors should consistently incorporate colonization differences into their interpretation and avoid causal language unless supported by direct in vivo evidence. At a minimum these statements should be backed up by the DAP-seq data.

<u>Our response:</u>

We thank the reviewer for this important comment and agree that reduced expression of AM-related genes in *idd7* mutants may partly result from decreased fungal colonization rather than direct transcriptional regulation by IDD7.

Accordingly, we have revised the Abstract to avoid overinterpretation of transcriptome data. Specifically, statements implying direct regulation have been replaced with more precise descriptions such as "IDD7 is required for the proper induction of AM-associated genes" or "IDD7-dependent gene expression changes." We also explicitly acknowledge that reduced gene expression in *idd7* may reflect both indirect effects of reduced colonization and direct transcriptional regulation (Lines 18-20).

Referee #1-6

3) Promoter selection and controls (Line 138). The authors test transactivation for PHR2, PT11, RAM1, and WRI5a promoters, but the selection criteria are unclear given the broader DEG list (Fig. 3c). Please clarify how these promoters were chosen and how many additional candidates were tested but did not respond to IDD7.

<u>Our response:</u>

We thank the reviewer for this helpful suggestion. The promoters of *PHR2*, *PT11*,

RAM1, and *WRI5a* were selected based on their well-established roles in AM symbiosis and their positions as key regulatory nodes within the transcriptional network. In particular, *PHR2*, *RAM1*, and *WRI5a* encode transcription factors that control the expression of multiple AM-associated genes, whereas *PT11* encodes a major phosphate transporter specifically induced in arbusculated cells. Therefore, these genes were chosen to represent central components of AM symbiotic signaling and nutrient exchange pathways.

To assess the specificity of IDD7-mediated transactivation, we also tested additional AM-related genes whose expression was reduced in *idd7* mutant. For example, the promoters of *ABCB* (a DEG identified in our RNA-seq dataset) and *VAPYRIN* (which showed reduced expression in *idd7* mutant at 7 wpi) were examined in transactivation assays. However, IDD7-VP16 did not activate the *ABCB* promoter and instead reduced *VAPYRIN* promoter activity under our experimental conditions (unpublished data).

These results indicate that IDD7 does not broadly activate all AM-associated genes but instead selectively regulates a subset of targets, consistent with our conclusion that IDD7 directly controls specific components of the AM transcriptional network.

We have revised the manuscript to clarify the selection criteria and the specificity of promoter activation (Lines 155-158, 161-165).

Referee #1-7

Also, the Nicotiana assays would benefit from stronger negative controls to evaluate artifact rates. For example, choose promoters not downregulated in idd7 but containing similar putative IDD motifs, and test whether they are transactivated in Nicotiana. This would help define specificity and reliability in this heterologous system. For all promoters IDD7 and PHR2 binding sites should be mutated as a specificity control.

Our response:

We thank the reviewer for this helpful suggestion. In the original manuscript, we included the *CCaMK* promoter as an additional control to evaluate the specificity of IDD7-mediated transcriptional regulation in the heterologous *Nicotiana* system. Although *CCaMK* was not identified as a DEG, its expression showed a decreasing trend in the mutant and was restored in one of the IDD7 complementation lines (Supplementary Fig. 4e), similar to the pattern observed for *PHR2*. Both the *PHR2* and *CCaMK* promoters contain previously reported IDD binding sites as well as DAP-seq–identified motifs (Supplementary Fig. 5a). In DLR assays, IDD7 in combination with SLR1 or PHR2 activated the *PHR2* promoter (Fig. 5a). In contrast, IDD7 with SLR1 did not activate the *CCaMK* promoter, while IDD7 together with PHR2 repressed its activity (Supplementary Fig. 7c). These findings suggest that the presence of putative IDD

binding motifs alone is not sufficient for transcriptional activation and support the specificity of the *Nicotiana* assay system.

In the original manuscript, we also showed that deletion of the P1BS element in the *PT11* promoter abolished PHR2-dependent activation and eliminated the additive effect with IDD7, indicating that this motif is required for transcriptional activation. In response to the reviewer's suggestion, we further mutated the P1BS element in the *RAM1* promoter and found that this similarly impaired PHR2-dependent activation (Supplementary Fig. 7e) (Lines 221-224, 548-550). Together, these results support that PHR2-mediated activation depends on intact cis-elements. Although this analysis was not extended to all promoters tested, the consistent results obtained from both *PT11* and *RAM1* support the generality of this mechanism.

For IDD7, EMSA assays indicate that binding is not fully abolished by mutations in the core motif, suggesting that flanking sequences contribute to binding specificity (Supplementary Fig. 8-9). This complicates the design of mutations that completely disrupt IDD7 binding and limits their utility as definitive specificity controls.

Referee #1-8

4) Protein – protein interaction should be supported by orthogonal experiments.

The conclusion that IDD7 interacts with PHR2 and SLR1 is currently based only on BiFC and split-luciferase assays in Nicotiana, which are susceptible to false positives/negatives due to heterologous overexpression and even more so due to covalent binding between the two halfs of the fluoreophore/lucifease. Additional orthogonal interaction evidence is needed to substantiate these claims, for example Y2H assay or co-IP.

<u>Our response:</u>

We thank the reviewer for these constructive suggestions. To address this concern, we performed additional experiments and included new data in the revised manuscript. Specifically, we added co-immunoprecipitation (Co-IP) assays in *Nicotiana benthamiana*, which provide additional support for the physical association of IDD7 with SLR1 and PHR2 *in planta*. These new results and corresponding descriptions have been incorporated into the revised manuscript (Supplementary Fig. 6b and Lines 174-176).

Referee #1-9

Problems: (1) Inconsistency between assays: e.g., Extended Data Fig. 6a suggests IDD7 △TQDFLG does not interact with PHR2, whereas Extended Data Fig. 6f suggests the opposite (although the signal is weaker than with wild type PHR2). (2) It would be good if the authors could show the level of protein accumulation in both assays.

Our response:

We thank the reviewer for pointing out this issue. We have now included immunoblot analyses for both the BiFC and LCI assays to monitor protein accumulation of the corresponding fusion proteins (revised Supplementary Fig. 6g and 6j).

In the BiFC assay, IDD7$^{\triangle TQDFLG}$ showed little or no detectable YFP complementation with PHR2, whereas the LCI assay still detected a weak interaction signal. Importantly, immunoblot analyses confirmed accumulation of the relevant fusion proteins in both assays. Therefore, the difference between the two assays is unlikely to result solely from lack of protein expression.

We interpret these results as indicating that deletion of the TQDFLG motif strongly weakens, but may not completely abolish, the interaction between IDD7 and PHR2. The apparent discrepancy between BiFC and LCI likely reflects differences in assay sensitivity, fusion configuration, or signal detection thresholds between the two systems. Accordingly, we have revised the manuscript text to avoid an absolute conclusion (Lines 167, 192-203).

Referee #1-10

Line 150: The authors suggest that the PHR2 – SLR1 interaction is weakened in the presence of IDD7, implying mutual exclusivity rather than a trimeric complex (Extended Data Fig. 6c).

Without protein level controls for all BiFC constructs in each condition (e.g., immunoblotting using anti-N-YFP/C-YFP tags), the loss/reduction of YFP could simply reflect altered abundance or stability of one fusion protein when co-expressed, as the leaf cells may be overloaded with production of three proteins.

Similarly, for the deletion analysis (Fig. 4), the authors infer that TQDFLG mediates interactions because IDD7△TQDFLG shows no YFP signal. This conclusion requires evidence that the deletion does not reduce protein stability/accumulation.

Our response:

For the comment regarding to PHR2-SLR1 interaction, we thank the reviewer for this important comment. To address this concern, we performed immunoblot analyses using anti-cYFP and anti-nYFP antibodies for all BiFC combinations under the corresponding co-expression conditions. The results are now included in Supplementary Fig. 6d. The immunoblot analysis showed that accumulation of some fusion proteins, particularly SLR1-containing constructs, was moderately reduced in the presence of 4xMYC-IDD7 during triple co-expression. Therefore, we agree that the reduced BiFC signal between PHR2 and SLR1 cannot be attributed solely to weakened protein interaction. Accordingly, we have revised the manuscript text to avoid overinterpretation regarding mutual exclusivity or exclusion of a trimeric complex. The

revised text now states that the PHR2–SLR1 BiFC signal decreased in the presence of IDD7, which is consistent with a possible influence of IDD7 on the PHR2–SLR1 association, although altered protein accumulation may also contribute to the reduced signal (Lines 180-185).

For the comment regarding to IDD7$^{\Delta TQDFLG}$ BiFC assay, we thank the reviewer for this important suggestion. To address this concern, we performed immunoblot analyses for the BiFC constructs using anti-cYFP and anti-nYFP antibodies. As shown in the revised Supplementary Fig. 6j, both wild-type IDD7 and IDD7$^{\Delta TQDFLG}$ fusion proteins were detectable in the corresponding co-expression combinations, indicating that the mutant proteins accumulated in planta and that the absence/reduction of BiFC signal was not simply due to complete loss of protein expression. However, because some differences in protein accumulation were observed between wild-type and mutant fusion proteins, we have revised the manuscript text to avoid overinterpreting the data. We now state that the TQDFLG motif substantially contributes to the IDD7–PHR2 interaction rather than concluding that it is absolutely required (Lines 167, 192-203).

Referee #1-11

Finally, please clarify how interaction "strength" (YFP intensity) is quantified: Are the normalization markers (e.g., NLS-mCherry) on the same plasmid? Are constructs delivered via separate Agrobacteria strains, and if so, how is the efficiency of transformation controlled? Ideally, the authors should normalize YFP to a co-expressed marker from the same plasmid to minimize variability from gene dosis (different amounts of plasmids per cell) and expression differences.

Our response:

We thank the referee for this important comment. We agree that BiFC fluorescence intensity can be influenced by transformation efficiency, construct expression, and gene dosage, particularly when the normalization marker is not encoded on the same plasmid.

In our experiments, the NLS-mCherry marker was not placed on the same BiFC plasmid. Therefore, we have revised the manuscript to clarify that YFP fluorescence was used as a relative BiFC signal, rather than as an absolute quantitative measurement of interaction strength. Constructs were delivered by separate *Agrobacterium* strains, and YFP images were acquired under identical microscope settings. Only cells showing nuclear mCherry fluorescence were selected for analysis, and YFP intensity was quantified using ImageJ after background subtraction. We have added these details to the Methods section (Lines 609-616).

Importantly, the conclusions drawn from BiFC intensity were supported by independent assays where possible. For the reduced interaction between IDD7$^{\Delta TQDFLG}$

and PHR2 or SLR1, the weaker BiFC signal was consistent with LCI assay. For the effect of IDD7 on the PHR2–SLR1 interaction, we now present the BiFC quantification as supportive evidence and rely primarily on the LCI assay to support this conclusion. We have revised the relevant text to avoid overinterpreting BiFC fluorescence intensity as a direct measure of interaction strength (Lines 180-185, 195-197, 1332-1336, 1354-1356).

Referee #1-12

5) Transactivation assay results in Nicotiana are confusing and suggest indirect effects In earlier figures (e.g., Fig. 5), the authors argue that IDD7 regulates PHR2 and PT11 because IDD7 ‑ VP16 and IDD7 + SLR1 transactivate these promoters. However, later results show that IDD7 ‑ VP16 can still activate promoters even when all putative IDD binding sites are removed. This weakens the claim that promoter activation depends on direct binding to those motifs and raises the possibility of indirect activation through other TFs in the transient system.

Given this, it would be better to support claims of direct DNA binding and direct regulation in vivo binding evidence such as ChIP ‑ qPCR/ChIP ‑ seq or cut and tag, and the transactivation assays in Nicotiana leaves should be interpreted with care.

Our response:

We thank the reviewer for this insightful comment. We agree that the transactivation results in *Nicotiana benthamiana* should be interpreted cautiously. The observation that IDD7–VP16 can still activate promoter reporters after removal of the predicted IDD-binding motifs suggests that transcriptional activation in this heterologous system may not depend exclusively on those motifs and may involve indirect mechanisms. To address this concern, we have revised the manuscript (Lines 349–353) to clarify that these assays support the transcriptional regulatory activity of IDD7 but should not be interpreted as definitive evidence of direct regulation in rice roots.

In response to this comment, we have revised the manuscript to clarify that the *Nicotiana* transactivation assays should not be taken as definitive evidence of motif-dependent direct regulation in vivo. Instead, we now present these assays as evidence that IDD7 has transcriptional regulatory activity and can promote activation of these promoter fragments in a transient plant system, while acknowledging that this system does not fully reproduce the regulatory context of rice roots undergoing AM symbiosis (Lines 310-312, 477).

At the same time, the EMSA results still support the biochemical capacity of IDD7 to bind specific promoter regions in vitro, and the genetic and expression data support a functional role for IDD7 in regulating AM-associated processes. To further strengthen the interaction aspect of the model in a rice system, we have now added BiFC assays

in rice protoplasts, which confirm the interaction between IDD7 and SLR1 in rice cells (Supplementary Fig. 6c). This new result strengthens the conclusion that the IDD7–SLR1 interaction is not limited to a heterologous *Nicotiana* system.

Because we currently lack suitable functional tagged lines and optimized reagents for ChIP or CUT&Tag, we are not yet able to provide chromatin-level validation of IDD7 occupancy in rice. We have therefore revised the manuscript to avoid overinterpreting the promoter activation data and to state more cautiously that the results are consistent with IDD7-mediated regulation of these candidate genes, rather than proving direct in vivo occupancy.

In the revised manuscript, we have:

1. revised the interpretation of the transactivation assays,
2. softened claims of motif-dependent direct regulation,
3. clarified the limitations of the heterologous transient system, and
4. added new BiFC data in rice protoplasts to strengthen the mechanistic model in a homologous rice context.

We hope this revision addresses the reviewer's concern and improves the clarity and rigor of our conclusions.

Referee #1-13

6）Other major points

Line 68 / Fig. S1 (phylogeny): The IDD7 phylogenetic tree appears unusual with all branches at the same level. Please verify the tree quality (e.g., inclusion of known IDD family members clustering as expected; alignment quality; appropriate model/method). Also please mention gene identifiers, not simply the names, as they are often inconsistent across publications.

<u>Our response:</u>

We thank the reviewer for this important comment. In response, we have re-constructed the phylogenetic tree of IDD proteins using a more rigorous and well-documented pipeline (Supplementary Fig. 1b).

Specifically, protein sequences from *A. thaliana*, *O. sativa subsp. japonica*, *Marchantia polymorpha*, *Medicago truncatula*, *Solanum lycopersicum*, and *Zea mays* were collected based on previously published annotations. Multiple sequence alignment was performed using MAFFT (v7.407) with the L-INS-i algorithm, and poorly aligned regions were removed using TrimAl (v1.4.1) with a gap threshold of 0.8. Phylogenetic analysis was conducted using the Maximum Likelihood method implemented in MEGA12. The best-fit substitution model (JTT+G) was selected based on model testing, and branch support was assessed using 1,000 bootstrap replicates (Lines 783-799, 1248-1256).

The revised tree shows a well-resolved hierarchical topology with improved branch support, and known IDD homologs across species cluster in a manner consistent with previously reported IDD phylogenies. To improve clarity and reproducibility, all sequences are now labeled with gene identifiers (locus IDs) rather than gene names alone. In addition, branches with bootstrap support values lower than 50% were collapsed in the final tree for clearer visualization.
These revisions address the reviewer’s concerns regarding tree quality, alignment, and annotation.

Referee #1-14
Figure callouts: numerous statements in the text lack clear figure references, making the manuscript difficult to follow. Please add figure citations consistently (e.g., “(Fig. X)” or “(Extended Data Fig. X)”). Examples: Line 210: “its coexpression with SLR1 or PHR2 was similar to that of full length IDD … (Fig. ?)” Line 225: “with P4 being the strongest (Fig. ?)”
Our response:
We thank the reviewer for this helpful comment. We have revised the manuscript to include consistent figure citations throughout the text (Supplementary Fig. 7g) (Line 246). In addition, we have carefully revised statements describing EMSA results to avoid overinterpretation of band intensity. Specifically, rather than stating that a particular probe shows the strongest binding, we now describe relative differences more cautiously (e.g., “variation in shift intensity across probes”), as EMSA band intensity is not strictly quantitative under these conditions (Supplementary Fig. 8b) (Line 262).
These revisions improve clarity and ensure that all statements are appropriately supported by the data.

Referee #1-15
Data presentation: For bar charts especially where differences are modest, please show all datapoints.
Our response:
We have revised all bar charts to display individual data points, ensuring full transparency and clearer visualization of modest differences.

Referee #1-16
Line 203 – 208: Even if PHR2 and IDD7 interact, overlap in downstream gene changes between mutants does not necessarily support or refute interaction because they could act redundantly or in parallel. The authors should avoid overinterpreting

expression overlap patterns.

Our response:

We thank the reviewer for this important point and agree that overlap in differentially expressed genes does not by itself demonstrate functional interaction between IDD7 and PHR2.

Accordingly, we have revised the Results section to avoid over interpretation of expression overlap and now describe these genes more cautiously as representing convergence of IDD7- and PHR2-dependent pathways (Lines 236-243). We explicitly state that such overlap may arise from direct coregulation, parallel regulation, or indirect effects, including those associated with altered AM colonization. We have also revised related statements describing IDD7–PHR2 cooperation to avoid implying a specific molecular mechanism that is not directly demonstrated by the data.

In addition, we have clarified this limitation in the Discussion section (Lines 420, 419-424), emphasizing that transcriptomic overlap alone does not constitute evidence of functional interaction.

Importantly, our conclusion regarding interaction between IDD7 and PHR2 is supported by independent evidence, including protein–protein interaction and cooperative transactivation assays.

Referee #1-17

Line 209: It is surprising that IDD7△TQDFLG activates PT11 alone. This could reflect altered protein stability/abundance. Do the authors see differential accumulation of IDD7 vs. IDD7△TQDFLG on a Western Blot?

Our response:

We thank the reviewer for this helpful suggestion. To determine whether the differential activation of PT11 by IDD7$^{\Delta TQDFLG}$ is due to altered protein stability or abundance, we examined protein accumulation using Western blot analysis.

This result has now been included in Supplementary Fig. 7g, alongside the corresponding DLR assay. The data show that IDD7 and IDD7$^{\Delta TQDFLG}$ accumulate at comparable levels, indicating that the observed difference in *PT11* activation is not due to differences in protein abundance, but rather reflects a functional effect of the TQDFLG motif.

Referee #1-18

Lines 174-214: This part is difficult to follow with different details for different promoters. Rephrasing for clarity is recommended.

Our response:

We thank the reviewer for this helpful suggestion. We agree that the original text

contained dense and repetitive descriptions of promoter-specific effects, which made the section difficult to follow.
We have substantially revised this section to improve clarity and readability (Lines 209-235). Specifically, we reorganized the paragraph to group results by regulatory logic (e.g., single-factor effects, cooperative activation, and promoter-specific variation) rather than describing each promoter individually. We also reduced repetitive phrasing and simplified the presentation of transactivation results.
In addition, we revised several statements to avoid overinterpretation of cooperative effects and to clearly distinguish between observed transcriptional outcomes and underlying mechanisms.
These changes improve the logical flow and make the section easier to follow.

Referee #1-19
Lines 216 – 234; Figs. S8 – S9: The EMSA section is difficult to follow and needs clearer presentation. This section is currently very hard to read because many similar short sequences are referenced without a clear organizing framework. Suggestions: Provide a summary table listing each probe (P1, P2, etc.) with the full 24 bp probe sequence (including flanks), and indicate which bases are mutated. Use a sequence logo to show degenerate positions and motif variability, rather than listing multiple motifs only in text. Clearly define "P motifs" when first introduced (it initially was unclear what P6 refers to around line 222). In Figs. S8/S9, avoid labeling probes only with short cores (e.g., "TTTGTG") when multiple probes share similar cores. it becomes impossible to reconcile why binding differs.
Our response:
We thank the reviewer for this helpful suggestion. We have revised the EMSA section (Lines 251-279) and associated figures (Supplementary Figs. S8–S9) to improve clarity and organization.
We added a summary table (Table S7–S8) listing all probes with their full 24-bp sequences, including flanking regions, and clearly indicated mutated nucleotides. We replaced motif descriptions in the text with sequence logos derived from MEME analysis, which better illustrate motif degeneracy and conserved positions. We also defined P motifs at their first occurrence, noting that they represent candidate IDD7-binding sites identified in this study and are labeled in the order of identification.
In addition, we revised figure labeling to use probe IDs (e.g., P1, M1) instead of short core sequences, and improved EMSA presentation by adding clear lane annotations and indicating shifted bands. Mutated nucleotides are now explicitly marked in probe sequences.
These changes improve the readability and interpretation of the EMSA data.

Referee #1-20

Figure 5c: There is large variation in the basal expression of PHR2 promoter version. Can this be explained?

Our response:

We thank the reviewer for this comment. The variation in basal activity among the *PHR2* promoter fragments is likely due to differences in the cis-regulatory elements retained in each truncated construct, reflecting the modular organization of plant promoters (Benfey and Chua 1990). All assays were performed under identical conditions and normalized to the REN internal control, indicating that this variation represents intrinsic promoter properties and does not affect the overall conclusion of Fig. 5c. We also add the explanation in the result section (Lines 331-333).

Referee #1-21

Extended data Figure 8: All EMSAs are lacking one important specificity control, which is the competition between the labelled WT probe with 10x, 50x and 100x of the unlabelled mutated probe. If the binding is specific the mutated unlabelled probe should not or hardly compete with the labelled probe. Furthermore, can IDD7 binding to the “mutated” P9 and P10 should be explained?

Our response:

We thank the reviewer for this important suggestion. We repeated the EMSA assays using newly purified IDD7 protein and included additional competition controls with increasing amounts (10×, 50×, and 100× molar excess) of unlabeled mutated probes. The mutated competitors exhibited greatly reduced competition compared with the WT competitors, supporting the specificity of IDD7 binding.

In the repeated assays, residual binding to the mutated P9 probe was no longer detected. This residual interaction with mutated P10 may reflect the degenerate nature of the IDD7-binding motif, in which partial sequence similarity and/or remaining conserved nucleotides can still support weak binding. We have updated Supplementary Figure 8 and revised the corresponding text accordingly (Lines 262-265, 271-274).

Referee #1-22

Line 285: It is unclear how EMSA probe sequences can be deleted. It would be better to use precise language and name the cis-element that was deleted. Generally deletion of cis-elements can change the structure or promoters, therefore mutation of cis-elements is preferable.

Our response:

We thank the reviewer for this suggestion. We have revised the text to clarify that promoter regions encompassing IDD-binding motifs were deleted. The specific regions (corresponding to EMSA probes P1–P9, M1–M5, etc.) are now explicitly described in the Methods section (Lines 532-548), along with their definitions in Supplementary Figs. 8 and 9. In addition, we clarify that deletion rather than mutation was used because mutation of individual motifs did not fully abolish IDD7 binding in EMSA assays (Lines 322-331, 338-341).

Referee #1-23

Target validation (Lines 297 – 303). The authors validate pPHR2 but claim many additional downstream targets. Given that major conclusions rely heavily on heterologous transient overexpression assays, additional evidence in rice (as with pPHR2 in idd7) for more proposed targets would substantially strengthen the work. Did the authors test additional candidate promoters/targets in the native context?

<u>Our response:</u>

We thank the reviewer for this important suggestion. We agree that validation of additional targets in the native rice context would further strengthen the conclusions. In this study, we successfully validated *PHR2* regulation in the *idd7* mutant background, demonstrating that IDD7 is essential for its expression in arbusculated cells.

We attempted to extend this analysis to additional candidate target, such as *PT11*, by generating corresponding promoter–reporter lines in the *idd7* mutant background. However, despite multiple transformation attempts, we were unable to obtain stable transgenic lines for this construct.

Nevertheless, our transient transactivation assays, together with EMSA and DAP-seq data, consistently support direct regulation of these promoters by IDD7. We have revised the text to clarify that, while PHR2 is validated in planta, regulation of additional targets is supported by complementary in vitro and transient assays (Lines 394, 400-402, 471-473).

Referee #1-24

CCaMK – CYCLOPS pathway interpretation (Line 305; Fig. S10)

Line 305: The current section does not demonstrate that IDD acts downstream of CCaMK – CYCLOPS. IDD7 expression could reflect colonization status rather than CCaMK – CYCLOPS regulation. This interpretation should be revised. Furthermore, CCaMK T265D is known to induce spontaneous nodules in legumes but it is not known to activate the RAM1 promoter together with CYCLOPS. In Pimprikar et al the kinase-only version of CCaMK (CCaMK 314) was used. Therefore, the data cannot be directly compared.

<u>Our response:</u>

Thank you for this insightful comment. We agree that our original wording could lead to an overinterpretation of the data.

First, we acknowledge that reduced *IDD7* expression in *ccamk* and *cyclops* mutants does not demonstrate that IDD7 is directly regulated by the CCaMK–CYCLOPS complex. Instead, the reduced expression may reflect impaired colonization and/or disruption of the common symbiosis signaling pathway (CSSP). We have therefore revised our interpretation to clarify that IDD7 functions downstream of CSSP in a broader sense, but is not directly regulated by CCaMK–CYCLOPS.

Second, we appreciate the reviewer's point regarding the difference between CCaMK constructs. In Pimprikar et al., a kinase-only, constitutively active CCaMK ($CCaMK^{1-314}$) was used, whereas in our study we used a gain-of-function mutant ($CCaMK^{T263D}$). As these constructs differ mechanistically, their activities are not directly comparable, and this likely contributes to the differing outcomes in *RAM1* promoter activation.

Consistent with this, our transactivation assays showed that $CCaMK^{T263D}$ together with CYCLOPS did not activate the *OsIDD7* or *OsRAM1* promoters, supporting the conclusion that these genes are not direct transcriptional targets of the CCaMK–CYCLOPS complex in our system.

To avoid ambiguity, we have revised the section title and text accordingly (Lines 363-381).

Referee #1-25

Line 335: "...supports a model in which PHR2 acts through IDD7-bound DNA..."

This is not strictly supported. The requirement for both factors in a reporter assay could also reflect indirect effects via downstream TFs induced by PHR2. The authors should rephrase more cautiously unless direct binding and mechanism are demonstrated.

<u>Our response:</u>

We thank the reviewer for this important comment and agree that the current data do not directly demonstrate that PHR2 acts through IDD7-bound DNA. The requirement for both factors in the reporter assay may reflect cooperative regulation, but does not by itself establish the underlying molecular mechanism.

Accordingly, we have revised the text to avoid over interpretation (Lines 415-419). We now describe the results more cautiously as indicating cooperative function between IDD7 and PHR2, without specifying the mechanism of action. We have also clarified that transcriptomic overlap between idd7 and *phr* mutants does not distinguish between direct coregulation, parallel pathways, or indirect effects (Lines 422-424).

Importantly, our conclusion that IDD7 and PHR2 functionally interact is supported by

multiple independent lines of evidence, including protein–protein interaction and cooperative transactivation assays, rather than the reporter assay alone.

Referee #1-26

Fig. S7c: Please explain the contrasting pCCaMK::LUC patterns under different factor combinations.

Our response:

Thank you for this helpful comment. We agree that the contrasting *pCCaMK::LUC* patterns under different factor combinations require clarification.

Our results suggest that *CCaMK* promoter activity is subject to context-dependent regulation by the tested factors. SLR1 appears to be associated with repression of *CCaMK* promoter activity, potentially through interaction with an unknown DNA-binding transcription factor. PHR2 alone also reduces *CCaMK* promoter activity. When IDD7 is co-expressed with SLR1, the observed change in promoter activity suggests that IDD7 may modulate SLR1-dependent regulation, although the underlying mechanism remains unclear. In contrast, combinations including PHR2 consistently show strong repression, indicating that PHR2 exerts a dominant negative effect on *CCaMK* promoter activity.

To avoid overinterpretation, we have revised the main text to describe these results more cautiously and emphasize the context-dependent nature of *CCaMK* promoter regulation rather than proposing a definitive mechanism (Lines 233-235).

Referee #1-27

Minor points

In several instances the author write that genes are ‘downregulated’ in the idd7 mutant. This seems to be a mis-conception as they are likely not actively downregulated but ‘less expressed’ either due to lower colonization in the mutant or the absence of induction by IDD7.

Our response:

Thank you for this helpful comment. We agree that the term “downregulated” may imply active repression, which is not directly supported by our data. In this study, the observed differences in gene expression in the idd7 mutant likely reflect reduced induction and/or differences in colonization rather than direct repression by IDD7. We have therefore revised the text throughout the manuscript to use more neutral terms such as “reduced expression” or “lower expression levels” where appropriate (Lines 121, 127, 137, 142, 144, 236, 237, 288, 428).

Referee #1-28

Line 71: Baseline pIDD7:GUS expression under mock conditions is important; consider moving this to a main figure.

<u>Our response:</u>

Thank you for this helpful suggestion. We agree that showing the baseline *pIDD7:GUS* expression under mock conditions is important for interpreting the induction pattern. Accordingly, we have moved this result from the supplementary data to the main figures, where it is now presented as Fig. 1b (Lines 77-78, 1150). This change improves the clarity and completeness of the expression analysis.

Referee #1-29

Line 80: Please state the length of the native promoter used and which terminator was used in the complementation construct.

<u>Our response:</u>

Thank you for this helpful suggestion. We have now clarified the length of the native promoter and the terminator used in the complementation construct in the revised manuscript (Lines 86-87).

Referee #1-30

Line 85: Please define RiITS1 when first mentioned. Furthermore, 'upregulated' would entail that the gene expression per fungal unit is increased. In reality the increased RiITS1 expression likely reflects increased colonization and this should be phrased as such.

<u>Our response:</u>

Thank you for this helpful comment. We have now defined *RiITS1* (the *Rhizophagus irregularis* internal transcribed spacer 1, used as a fungal colonization marker) at its first occurrence in the revised manuscript. We also agree that the term "upregulated" may be misleading in this context. The increased *RiITS1* signal likely reflects higher fungal colonization rather than increased expression per fungal unit. Therefore, we have revised the wording accordingly (Lines 91-93).

Referee #1-31

Line 87: The two complementation lines show dramatically different expression levels of IDD and downstream genes; please discuss why. An additional independent line to support conclusions about native promoter functionality could be helpful.

<u>Our response:</u>

We thank the reviewer for this helpful suggestion. We analyzed a total of nine independent T1 complementation lines, which displayed varying levels of *IDD7* expression, likely reflecting positional effects of transgene insertion. Among these, we

selected lines #6 and #13 for detailed analysis because they showed the highest *IDD7* expression levels and most robust phenotypic complementation.
The observed differences in *IDD7* and downstream gene expression between these two lines are consistent with typical variation among independent transgenic lines and do not affect the overall conclusion, as both lines exhibit consistent trends in downstream gene regulation and phenotypic rescue. We have clarified it in the text (Lines 95-96).
While we agree that additional independent lines can further support the conclusions, the analysis of multiple T1 lines (n = 9) prior to selection, together with the consistent results obtained from the two representative high-expressing lines, provides strong support for the functionality of the native promoter-driven IDD7 construct.

Referee #1-32
Use of two WT controls (Dongjin and an azygous line) is confusing, although not incorrect. For Fig. 2c (and elsewhere), clarify which control is the appropriate comparator for idd7 and why.
Our response:
We thank the reviewer for raising this important point. The azygous line represents the segregating wild-type derived from the *idd7* heterozygous background and is therefore the most appropriate genetic control for comparison with the *idd7* mutant and complementation lines, whereas Dongjin is the parental cultivar.
We note that fungal colonization differs slightly between these controls in a time-dependent manner: at 5 wpi, colonization is lower in the azygous line compared to Dongjin, whereas at 7 wpi the levels are comparable. Importantly, the complementation line (#13), in which *IDD7* expression is not fully restored to the azygous level, nevertheless rescues the colonization phenotype to the level of the azygous control, supporting functional complementation.
We have clarified in the text that the azygous line is the primary comparator (Lines 99-103).

Referee #1-33
Line 101: This headline seems incomplete. Did the authors mean “IDD7 regulates genes involved in nutrient exchange and signaling”? The same is true for the headline in line 146, 174, 216, 305.
Our response:
We thank the reviewer for pointing out the lack of clarity in several section headings. We have revised the title in Line 101 to explicitly reflect the regulatory role of IDD7 in both nutrient exchange and signaling during AM symbiosis.

In addition, we have carefully revised the other section headings (Lines 146, 174, 216, and 305) to ensure they are complete, grammatically correct, and accurately describe the corresponding results. These changes improve clarity and consistency throughout the manuscript (Lines 113-114, 167, 209, 251, 363-364).

Referee #1-34

Line 102: What do the authors mean with ‘downstream role’: Should this read: ‘role in regulating downstream genes’?

Our response:

We thank the reviewer for this helpful suggestion. We agree that the phrase “downstream role” was ambiguous. We have revised the sentence to clarify that we are referring to the role of IDD7 in regulating downstream genes during AM symbiosis (Lines 115).

Referee #1-35

Line 178: How does SLR1 activate the CYCLOPS promoter alone? This should be discussed as GRAS factors are generally known not to bind DNA.

Our response:

We thank the reviewer for this insightful comment. GRAS family proteins, including SLR1, are generally not considered to bind DNA directly. Therefore, the observed activation of the CYCLOPS promoter by SLR1 alone likely occurs indirectly. One plausible explanation is that SLR1 interacts with endogenous tobacco transcription factors that are capable of binding the CYCLOPS promoter, thereby functioning as a co-regulator to modulate transcription. Alternatively, SLR1 may influence transcription through protein–protein interactions with other components of the transcriptional machinery. Similar indirect regulatory roles have been reported for GRAS proteins in other contexts. We have added a brief discussion in the revised manuscript to clarify this point (Lines 213, 412-415).

Referee #1-36

Line 200 (Extended Data Fig. 7c): If IDD7 – VP16 can transactivate promoters lacking IDD binding sites, interpretations of “IDD7 requires a partner to activate this promoter” should be reconsidered.

Our response:

Thank you for this important comment. We would like to clarify the interpretation of the IDD7–VP16 transactivation results.

In our study, the *CCaMK* promoter used in Supplementary Fig. 7c contains predicted IDD-binding sites (as shown in Supplementary Fig. 5a). Therefore, the activation

observed with IDD7–VP16 is consistent with direct binding of IDD7 to its cognate sites in the *CCaMK* promoter.

The VP16 domain functions as a strong transcriptional activation domain, effectively mimicking the activity of a co-activator that may normally interact with IDD7. Thus, the IDD7–VP16 fusion artificially bypasses the requirement for endogenous partner proteins by providing activation capability in cis. In contrast, native IDD7 lacks a strong activation domain and therefore likely requires additional cofactors to activate transcription.

Based on this, our interpretation is not that IDD7 binds promoters lacking IDD-binding sites, but rather that IDD7 alone is insufficient to activate transcription efficiently and requires a partner with activation activity, which is functionally substituted by VP16 in this assay.

To avoid misunderstanding, we have revised the text to clarify that the requirement for a partner refers to transcriptional activation rather than DNA binding (Lines 231-233).

Referee #1-37

Line 221: Why are TTGTCT (5´-AGACAA-3´), AGACAG, and TTTGGT in the PHR3 promoter specifically mentioned? Are these established IDD binding sites?

<u>Our response:</u>

We thank the reviewer for this important point. The sequences TTGTCT (5'-AGACAA-3'), AGACAG, and TTTGGT were mentioned because they are previously reported IDD-binding motifs identified in different studies. However, we agree that this was not sufficiently explained in the original text and could lead to confusion.

To improve clarity, we have revised the paragraph to more systematically describe our motif analysis. Specifically, we now present a conserved but degenerate core motif (most commonly centered on TTTGTG/C) identified in our analysis, and define candidate binding sites within the promoter as P motifs (P1–P9) for systematic evaluation by EMSA.

These revisions clarify that the tested motifs include both previously reported IDD-binding sequences and related variants identified through motif analysis, providing a more comprehensive and unbiased framework for assessing IDD7 binding (Lines 252-259).

Referee #1-38

Please clarify motif differences and binding outcomes between AGACAG in PHR2 (Fig. S8 P7/P8) and PT11 (Fig. S8d P6), and between TTTGGT (Fig. S8d P7) and probes with mobility shifts (e.g., Fig. S8d P9/P10; Fig. S8b P9). A sequence logo would help readers

understand which positions are truly informative.

Our response:

Thank you for this helpful suggestion. We agree that the previous presentation did not sufficiently clarify the sequence features associated with IDD7 binding. To address this, we compared the motifs from probes showing binding versus those that did not and generated sequence logos for the AGACAG- and TTTGGT-containing probe sets.

For the AGACAG-related probes, both the PHR2 probes (Fig. S8, P7/P8) and the PT11 probe (Fig. S8d, P6) contain the AGACAG core, but their flanking sequences differ. Comparison of these sequences suggests that, in addition to the AGACAG core, an upstream A residue may also contribute to IDD7 binding, consistent with the sequence logo in which this position shows enrichment. Thus, the AGACAG motif alone may not be fully sufficient, and neighboring bases likely influence binding affinity.

For the TTTGGT-related probes, the canonical TTTGGT core is evident in Fig. S8d (P7), but comparison with shifted probes such as Fig. S8d (P9/P10) and Fig. S8b (P9) suggests that an additional upstream G residue may also be important for efficient IDD7 binding. The sequence logo supports this interpretation by showing enrichment of G at this position among bound probes.

We have now added the sequence logos (Supplementary Fig. S8e-f) and revised the text to clarify that (Lines 275-278, 1395-1405), while AGACAG and TTTGGT represent the principal core motifs, adjacent nucleotides also appear to contribute to binding specificity.

Referee #1-39

Line 265 – 271: If a mutated IDD7 shows auto-activation, did the authors test whether the mutation also alters DNA-binding properties?

Our response:

We thank the reviewer for this insightful question. We did not directly test whether the mutated IDD7 protein alters its DNA-binding capacity (e.g., by EMSA or related assays).

Our AlphaFold3-based structural modeling indicates that DNA recognition by IDD7 is primarily mediated by residues within the N-terminal zinc finger domains, including Arg77 (ZF1) and Arg179 (ZF4), which interact with the minor and major grooves of DNA, respectively (Supplementary Fig. 9l). "In contrast, the IDD7$^{\Delta TQDFLG}$ mutant analyzed in this study lacks the C-terminal TQDFLG domain, which is spatially separated from the predicted DNA-contacting interfaces.

This spatial separation suggests that the mutation is less likely to directly impair DNA binding. Instead, the C-terminal region, including the TQDFLG domain, may contribute to transcriptional regulatory functions, such as protein–protein interactions or

activation potential. Therefore, we speculate that the observed auto-activation phenotype arises from altered transcriptional activity rather than changes in DNA-binding affinity.

Nevertheless, we cannot exclude the possibility that the mutation indirectly affects DNA binding or chromatin association. Direct biochemical assays, such as EMSA or ChIP-based approaches, will be required to fully address this question and represent an important direction for future investigation.

Referee #1-40

Line 276: “in which IDD motifs defined using a 4 – 10 bp mismatch/gap tolerance were deleted” needs grammatical correction (and similar phrasing elsewhere should be edited).

Our response:

We thank the reviewer for pointing out this issue. We have revised the sentence for grammatical clarity and consistency. Specifically, “in which IDD motifs defined using a 4–10 bp mismatch/gap tolerance were deleted” has been rephrased to improve readability (ex. Line 325), and similar expressions have been corrected throughout the manuscript.

Referee #1-41

Discussion and Fig. 6: The authors state that CCaMK and CYCLOPS are expressed only in the epidermis. Has this been shown in rice?

Our response:

We thank the reviewer for this important point. We agree that our original wording could be interpreted as implying epidermis-specific expression of CCaMK and CYCLOPS in rice, which is not directly demonstrated in the cited study. We have therefore revised the text to clarify that CCaMK and CYCLOPS mediate signaling required for AM symbiosis, rather than implying strict epidermis-specific expression. The citation has been retained to support their essential role in AM signaling, but the wording has been adjusted to avoid overinterpretation (Lines 459-460, 467-468, 1223-1224).

Answers to Referee #2

Referee #2-1

Below I have several suggestions and comments. Especially, a confirmation of the suggested interactions in roots would strengthen the work.

Referee #2-7

It would be good to verify the interactions in roots using co-IP to confirm that the interactions truly occur in the relevant rice tissue.

Our response:

We thank the reviewer for these constructive suggestions regarding validation of the IDD7 interactions in relevant rice tissues. To address this concern, we performed additional experiments and included new data in the revised manuscript.

Specifically, we now show that IDD7 interacts with both the GRAS transcription factor SLR1 and the MYB transcription factor PHR2 in rice protoplasts using BiFC assays, providing validation in a homologous rice system. In addition, we added Co-immunoprecipitation (Co-IP) assays in *Nicotiana benthamiana*, provide additional support the physical association of IDD7 with SLR1 and PHR2 in planta.

We fully agree that direct validation in rice root tissues would further strengthen the physiological relevance of these interactions. We attempted to isolate rice root protoplasts for BiFC assays; however, the yield and viability of root-derived protoplasts were insufficient for downstream transformation experiments. In addition, we currently do not have stable transgenic rice materials suitable for Co-IP analyses in rice roots. Despite these technical limitations, the combined evidence from BiFC, LCI, and Co-IP assays across multiple systems consistently supports the robustness of the IDD7–SLR1 and IDD7–PHR2 interactions.

These new results and clarifications have been incorporated into the revised manuscript (Supplementary Fig. 6b-c and Lines 174-178, 473-474, 476).

Referee #2-2

According to Supplementary table 1, additional IDD genes in rice may show induced expression upon AMS. Did the authors test the expression of other IDD genes by qPCR as they did for IDD7?

Our response:

Thank you for this insightful suggestion. Based on Supplementary Table 1, several *IDD* genes besides IDD7 show potential differential expression under AM symbiosis. To further evaluate this, we performed qPCR analysis on a subset of IDD genes.

Our results show that:

IDD9 expression is significantly induced by AM symbiosis at 5 and 7 weeks post-inoculation (wpi). *IDD14* expression is induced at 3, 5, and 7 wpi. *IDD12* shows induction at 5 wpi. In contrast, *IDD4.3* and *IDD13.1* do not exhibit significant changes in expression under AM symbiosis.

These results partially support the transcriptomic datasets and indicate that, in addition to *IDD7*, a subset of IDD family members is responsive to AM colonization.

We have now included these qPCR results in the revised manuscript (see Supplementary Fig. 1a), and updated the Results section accordingly (Lines 66-73, 1241-1248).

Referee #2-3

Is the arbuscule specific expression of IDDs also supported by public available single cell data?

Our response:

We thank the reviewer for this excellent point. Yes, the arbuscule-specific/enriched expression of specific IDD transcription factors is directly supported by recent, publicly available single-nucleus RNA sequencing (snRNA-seq) data.

Reference: "Decoding stage-specific symbiotic programs in the arbuscular mycorrhizal symbiosis at single-nucleus resolution" (Stuer et al. 2026).

Concrete Evidence:

By tracking individual root cell trajectories during symbiosis, this study bypassed bulk root noise to isolate colonized inner cortex cells. The single-nucleus dataset explicitly identifies members of the plant-specific indeterminate-domain (IDD) family, such as *SlNUC* (*Solyc09g074780.3*), as highly enriched and specifically induced within the precise cell clusters undergoing active arbuscule development. This provides high-resolution, cell-type-specific transcriptomic validation for the localized role of IDDs.

We have incorporated this reference into the revised manuscript (Lines 385-388).

Referee #2-4

IDD proteins have been shown to be mobile. It would be good to check the localization of an IDD7 GFP fusion.

Our response:

Thank you for this helpful suggestion. IDD proteins are generally characterized as nuclear transcription factors, and their functions have primarily been described as cell-autonomous. While intercellular mobility has been reported for some transcription factors, such as GRAS family regulator SHORT-ROOT (SHR), there is currently limited evidence supporting cell-to-cell mobility of IDD proteins.

In fact, we have already examined the subcellular localization of IDD7 in the original manuscript. As shown in Supplementary Figure 1c, an IDD7–GFP fusion protein expressed in *Nicotiana benthamiana* leaves is localized exclusively to the nucleus, consistent with its predicted role as a transcription factor. These results support that IDD7 functions as a nuclear protein.

Referee #2-5

Briefly explain the Azygous control in the text (line 103).

Our response:

Thank you for this helpful suggestion. The azygous segregant, derived from the *idd7*

heterozygous background, was used as the primary wild-type control. We have now revised the text to clarify that (Line 99).

Referee #2-6

OsRAM1 did not interact with OsIDD7 in the BiFC assay. Did the authors check the expression of RAM1 at the protein level in Nicotiana leaves, to make sure that it was properly expressed?

Our response:

We appreciate the reviewer's concern. To confirm that OsRAM1 was properly expressed in *Nicotiana benthamiana* leaves during the BiFC assay, we performed western blot analysis using protein extracts from the infiltrated leaves. The results confirmed that OsRAM1 was successfully expressed at the protein level. Therefore, the lack of BiFC signal between OsRAM1 and OsIDD7 is unlikely to be caused by failed expression of OsRAM1 (Supplementary Fig. 6j).

Referee #2-8

Line 246, explain IGV.

Thank you for this helpful suggestion. IGV is "Integrative Genomic Viewer". We have now revised the text to clarify that (Line 292).

Referee #2-9

Line 275, clarify △Pub-IDDBM.

Our response:

We thank the reviewer for this suggestion. We have clarified the meaning of ΔPub-IDDBM in the revised manuscript. Specifically, ΔPub-IDDBM now explicitly refers to deletion of EMSA probe sequences containing IDD-binding motifs previously reported in the literature. This clarification has been added at the first occurrence of the term to improve readability (Line 324).

Referee #2-10

Line 371, maybe indicate more clearly that this is based on predictions. No validation experiments are presented.

Our response:

Thank you for this helpful suggestion. We agree that this conclusion is based on structural prediction and has not yet been experimentally validated. We have revised the sentence to more clearly indicate that this interpretation is derived from our structural modeling analysis (Lines 454, 456).

Referee #2-11

In the final model (Fig. 6) the negative regulation of CCaMK expression by IDD7 is indicated. This is based on the transactivation in Nicotiana leaves. Given a lack of verification of this downregulation in arbuscule containing cells (or rice roots), I would suggest to remove this speculative result from the model.

<u>Our response:</u>

Thank you for this constructive suggestion. We agree that the negative regulation of *CCaMK* by IDD7, which is based on transactivation assays in *Nicotiana benthamiana*, lacks direct validation in arbuscule-containing cells or rice roots. Therefore, we have removed this speculative regulatory relationship from the model in Fig. 6 in the revised manuscript.

Reference

Benfey PN, Chua NH (1990) The cauliflower mosaic virus 35S promoter: combinatorial regulation of transcription in plants. Science 250 (4983):959-966

Shi J, Zhao B, Zheng S, Zhang X, Wang X, Dong W, Xie Q, Wang G, Xiao Y, Chen F, Yu N, Wang E (2021) A phosphate starvation response-centered network regulates mycorrhizal symbiosis. Cell 184 (22):5527-5540.e5518. doi:10.1016/j.cell.2021.09.030

Stuer N, Leroy T, Eekhout T, De Keyser A, Staut J, De Rybel B, Vandepoele K, Van Damme P, Van Dingenen J, Goormachtig S (2026) Decoding stage-specific symbiotic programs in the Rhizophagus irregularis–tomato interaction using single-nucleus transcriptomics. Preprint at bioRxiv. doi:https://doi.org/10.64898/2026.01.22.701092

Title of manuscript: OsIDD7 integrates signaling networks for arbuscular mycorrhizal symbiosis

Manuscript number: NCOMMS-26-007876B

We are grateful for the suggestions and comments to improve our manuscript. Below are our point-by-point responses to the editor and reviewers' comments.

Answers to Referee #1

The manuscript has been improved however, some of the data (e. g. the phylogenetic tree and EMSAs) and also the writing have not yet reached publication quality. Some figures stretch over multiple pages, which makes understanding them cumbersome. The authors could consider reducing the size of the panels to fit the figure onto one page or to divide the figure into several.

Our response:

We thank the reviewer for this helpful suggestion. We have carefully revised the presentation of the supplementary figures to improve their overall readability. In particular, we reorganized the EMSA figures, adjusted the panel layout and spacing, and avoided splitting individual panels across page breaks. For figures that could not be presented clearly on a single page without substantially reducing the panel size, we allowed the figure to continue onto the following page (e.g., "Supplementary Fig. X (continued)") to preserve the visibility of the EMSA bands. We have also revised the organization of the supplementary figures where appropriate to improve the flow and readability of the data presentation.

Comments:

Referee #1-1

Line 42: Please note that Lotus CBX1 serves as critical regulator of nutrient exchange genes in complex with RAM1 (Paries et al 2025, PNAS). This should be mentioned in the introduction.

Our response:

We thank the reviewer for bringing this important study to our attention. We have now cited the recent work by Paries et al. (2025) in the Introduction and revised the corresponding paragraph to acknowledge that *Lotus japonicus* CBX1 functions together with RAM1 to regulate nutrient exchange genes during AM symbiosis. This finding further supports the importance of transcription factor complexes in controlling arbuscule function.

The Introduction has been revised accordingly (Lines 45-47).

Referee #1-2

Figure 1b: The phylogenetic tree is practically not improved from the last submission and still contains many polytomies, which limits the usefulness of the tree. Especially the location of the protein of interest OsIDD7 in the phylogeny is ambiguous. I suggest to either remove the tree entirely (as in the current version it does not add useful information to the manuscript) or to fix the polytomies by 1) adding more data and 2) by rooting the tree. Especially adding more dicot sequences and at least a second Solanaceaous species, a second Brassicacae species and a second legume, may help to turn the polytomies into dichotomies.

<u>Our response:</u>

We thank the reviewer for this helpful suggestion. We agree that the phylogenetic analysis is not essential for the main conclusions of this study. Since the tree does not contribute directly to the mechanistic findings regarding IDD7 function during AM symbiosis, we have removed the phylogenetic tree from the manuscript and Supplementary Information. The corresponding description in the Results has also been removed.

Referee #1-3

Fig. 5 and Fig S5b: The transactivation of target promoters is quite poor and for some promoters not even 2-fold. I wonder whether the chosen promoters are really bona fide targets and/or whether IDD7-VP16 or IDD7 in complex with PHR2 and SLR1 is sufficient to explain their induction.

<u>Our response:</u>

We thank the reviewer for this thoughtful comment. We agree that the transient transactivation assays should be interpreted cautiously and are not intended to demonstrate that IDD7 alone is a strong transcriptional activator or that the IDD7-containing complexes are sufficient to account for the full induction of these genes during AM symbiosis.

We note, however, that relative to the empty vector control, all promoter activation events discussed in Fig. 5 and Supplementary Fig. 5b exceeded twofold, with several promoter–effector combinations exhibiting approximately three- to fivefold activation. To improve clarity, we have added the corresponding fold activation values to the figures (Lines 1166-1167, 1258-1259). Fold-change labels were not added to the promoter-deletion assays because these experiments were intended to assess the effect of cis-element removal on reporter activity, for which the normalized LUC/REN values and statistical comparisons provide the more direct readout.

Nevertheless, we agree that the observed activation is likely to represent only part of the transcriptional regulatory mechanism. The transient assays were performed in a heterologous *Nicotiana benthamiana* system and therefore do not fully recapitulate

the regulatory environment of arbuscule-containing rice cells. Additional transcription factors, chromatin context, and AM-specific signaling are likely required for full activation in planta. Accordingly, we have revised the Discussion (Lines 440-447) to clarify that these assays support cooperative transcriptional regulation by IDD7 in combination with SLR1 and/or PHR2, rather than demonstrating that these complexes alone are sufficient to explain the complete induction of the target genes during AM symbiosis.

Referee #1-4

The EMSAs are lacking an important control: The mutated cold probe in the same dilution series as the cold WT probe.

Our response:

We thank the reviewer for highlighting the importance of including mutated competitors in the EMSA assays. We have now repeated representative EMSAs for the DAP-seq/MEME-derived M1–M5 motifs using increasing concentrations of unlabeled WT and mutated competitors (10×, 50×, and 100× molar excess) (Supplementary Fig. 9, Lines 1373-1377). The repeated EMSAs showed that excess unlabeled WT competitors generally reduced the mobility shifts, whereas mutations altered the competition and mobility-shift patterns in a motif-dependent manner. In addition, several mutant probes retained detectable mobility shifts, indicating that the effects of the mutations varied among individual motifs. Together, these results provide additional support for sequence-dependent DNA recognition by IDD7 (Lines 300-315). For the candidate M6- and M7-containing regions of the *PT11* promoter, we did not generate mutated competitors because these regions were identified using a relatively permissive sequence-matching threshold, and the MEME-derived matches extend across long contiguous sequences without clearly defined short core motifs suitable for targeted mutagenesis. Extensive mutation of these regions would substantially alter the overall probe sequence and therefore would not constitute an interpretable motif-specific control. We therefore retained the WT competition assays for these probes but revised the manuscript to clarify that these experiments demonstrate binding to the tested promoter fragments rather than establish specific recognition of the predicted M6 or M7 motifs (Lines 345-349).

Referee #1-5

Fig. S9e, please indicate the direction of the genes.

Our response:

We thank the reviewer for this helpful suggestion. Although the transcriptional direction was originally indicated by the arrowheads within the gene model, we agree

that this was not sufficiently obvious. We have therefore revised Supplementary Fig. 9e by adding a directional arrow to explicitly indicate the direction of transcription, making the gene orientation easier to recognize (Lines 1361-1362).

Referee #1-6

Line 270: Binding strength can only be estimated by determining the Kd of the binding (e. g. by microscalte thermophoresis or similar methods). I suggest to rephrase for rigor: "The quantity of the shifted probe varied, potentially suggesting differences in binding strength…".

Our response:

We thank the reviewer for this suggestion. We agree that EMSA does not allow quantitative determination of binding affinity. Accordingly, we have revised the wording throughout the manuscript by replacing "binding strength" with more appropriate descriptions such as "the quantity of shifted probe varied" or "the observed shift intensity differed among probes," avoiding overinterpretation of the EMSA data (Line 272).

Referee #1-7

Line 313: The text should reflect that alpha-fold can only make predictions. E. g. the word 'reveal' should be replaced by 'predicted' etc.

Our response:

We thank the reviewer for this suggestion. We have revised the manuscript throughout to clarify that the structural analyses are AlphaFold3 predictions. Terms such as "revealed" have been replaced with "predicted" where appropriate (Line 317).

Referee #1-8

Line 349-353: The transactivation of promoters from which all IDD7 binding sites have been mutated by IDD7-VP16 may indicate unspecific transactivation. This may potentially lead to the rejection of the hypothesis that IDD7 specifically regulates the chosen target promoters. This notion is supported by the relatively weak induction of promoters as compared to background activation in absence of IDD7. Alternatively, more TFs are required to strongly induce the promoters.

Our response:

We thank the reviewer for this insightful comment. We agree that the transactivation results obtained in *Nicotiana benthamiana* should be interpreted cautiously. Accordingly, we have substantially revised this section of the manuscript. We now explicitly state that activation of the deletion constructs indicates that transcriptional activation in this heterologous system does not strictly depend on the predicted IDD-

binding motifs and may involve indirect effects mediated by endogenous transcription factors or additional cis-elements. Therefore, these assays are no longer presented as definitive evidence of direct motif-dependent regulation in vivo (Lines 352-359, 452-458).

Referee #1-9

Line 372 and following: This is a misinterpretation. The assay in S10g cannot be interpreted because CYCLOPS phosphorylation by the truncated CCaMK only containing the kinase domain is required for induction of the RAM1 promoter and other AM-induced promoters. Furthermore, in this assay the EV control for most promoters except the IDD7 promoter are missing.

<u>Our response:</u>

We thank the reviewer for this important comment. We agree that the transient transactivation assay cannot be directly compared with the previously reported constitutively active kinase system and therefore does not allow conclusions regarding direct regulation by the CCaMK–CYCLOPS module. Accordingly, we have removed the corresponding interpretation and substantially shortened this section. We now simply conclude that, under our experimental conditions, OsCYCLOPS together with the CCaMK construct used in this study did not activate the tested promoters (Lines 378-382).

Overall, the manuscript text is written in a lengthy and partially repetitive manner. It would be good to revise it for better readability.

<u>Our response:</u>

We thank the reviewer for this valuable suggestion. We have carefully revised the manuscript throughout to improve readability. In the Results section, we reduced repetitive descriptions of RNA-seq, EMSA, and DAP-seq analyses, simplified several long sentences, and removed redundant summaries. In the Discussion, we streamlined repetitive interpretations while retaining important caveats and reviewer-requested clarifications. We also corrected minor grammatical issues and improved the logical flow between paragraphs. Overall, these revisions substantially improve the readability of the manuscript without changing the scientific conclusions.

Minor comments:

Referee #1-10

Throughout the manuscript I suggest to use ‘arbuscule-containing cells’ instead of ‘arbusculated cells’.

Our response:

Thank you for this helpful suggestion. We have now revised the text according to the suggestion.

Referee #1-11

Line 74-76: The reason for mentioning the tree here is not very clear, as it seems more intuitive to continue from with expression pattern analysis from qPCR to promoter:reporter assays. If there is a reason for describing the tree before the promoter:GUS assay this should be introduced/explained in a way that the reader can understand.

Our response:

We thank the reviewer for this helpful suggestion. We have removed the phylogenetic tree from the manuscript and Supplementary Information. The corresponding description in the Results has also been removed.

Referee #1-12

Line 81: Do the authors mean 'N. benthamiana leaves'?

Our response:

Thank you for this helpful suggestion. Yes, we have now revised the text according to the suggestion (Line 82).

Correct.

Referee #1-13

Fig. 2C is very crowded and difficult to grasp. I suggest to separate the bars indicating fungal structures by a vertical dotted line (or similar).

Our response:

We thank the reviewer for this suggestion. We have revised Fig. 2c by separating the fungal colonization categories with vertical divider lines to improve readability.

Referee #1-14

Line 188: 'the complementation results' should be rephrased for readability. Do the authors mean: 'the results from the complementation experiment'?

Our response:

Thank you for this helpful suggestion. We have now revised the text according to the suggestion (Lines 120-121).

Referee #1-15

Line 163: I am not sure if 'data not shown' is permitted by the journal. In any case if

these promoters are used as negative controls it is important to show the data.

Our response:

We thank the reviewer for this suggestion. We agree that results referred to as "data not shown" should either be presented or removed. We have therefore included the complete dual-luciferase datasets for the *ABCB* and *VAPYRIN* promoters in Supplementary Fig. 5b (Lines 163-169). These experiments contained multiple effector combinations and were analyzed by one-way ANOVA followed by the indicated multiple-comparison test. In this analysis, IDD7–VP16 did not significantly increase *ABCB* or *VAPYRIN* promoter activity relative to EV after correction for multiple comparisons. We have revised the text to describe the results and statistical framework more precisely.

Referee #1-16

Line 184: I suggest to rephrase the sentence: "Therefore, the results suggest, that IDD7 may affect …" (do not conclusively demonstrate weakens the narrative unnessessarily, 'suggest' and 'may' is careful enough in my opinion).

Our response:

Thank you for this helpful suggestion. We have now revised the text according to the suggestion (Lines 186-187).

Referee #1-17

Line 199: This statement is vague, describe which differences in protein level were observed.

Our response:

We thank the reviewer for this suggestion. We have revised the Results to explicitly describe the protein accumulation observed in the immunoblot analysis (Lines 198-201). Most BiFC fusion proteins accumulated at comparable levels; however, the SLR1-cYFP and IDD7$^{\Delta TQDFLG}$-nYFP signals were moderately reduced compared with the corresponding WT IDD7–SLR1 combination. Despite this modest reduction in protein accumulation, the loss of the BiFC signal for the SLR1–IDD7$^{\Delta TQDFLG}$ pair was substantially greater than expected from the observed protein levels, supporting the conclusion that deletion of the TQDFLG motif markedly impairs the interaction between IDD7 and SLR1.

Referee #1-18

Figure S7 is difficult to read because the transactivation assays with mutated promoters are far away from the respective WT promoters. For comparison it would be helpful to show WT and mutated promoters side-by-side.

<u>Our response:</u>

We thank the reviewer for this helpful suggestion. To facilitate direct comparison, we reorganized Supplementary Fig. 7 so that the WT and corresponding mutant promoter data are presented side-by-side. For the *PT11* promoter, the WT data shown in Fig. 5b are reproduced in Supplementary Fig. 7 solely for comparison with the ΔP1BS construct (Lines 1303-1316). This reorganization improves figure readability without affecting the underlying data.

Referee #2

Reviewer #2 (Remarks to the Author):

The revised manuscript by Chen and colleagues reveals an important novel role for rice IDD7 in the signaling network for arbuscular mycorrhizal symbiosis. They show that IDD7 can interacting with PHR2 and DELLA SLR1, to synergistically regulate important genes for arbuscule development and functioning.

Although validation of interactions remains to be proven in the relevant rice root tissues the data make a strong case for the central role of OsIDD7 (and possibly additional IDDs). This work is the first to show the involvement of IDDs in the symbiosis and is expected to inspire a further unraveling of their role in other plant species as well.

In my view the authors have sufficiently addressed the concerns raised by the reviewers.

<u>Our response:</u>

We sincerely thank the reviewer for the positive evaluation of our work and for the constructive comments throughout the review process. We greatly appreciate the reviewer's recognition of the significance of our findings and are pleased that the revised manuscript has satisfactorily addressed the previous concerns.
